# The Recombinant Fragment of Human κ-Casein Induces Cell Death by Targeting the Proteins of Mitochondrial Import in Breast Cancer Cells

**DOI:** 10.3390/cancers12061427

**Published:** 2020-05-31

**Authors:** Max Richter, Fabian Wohlfromm, Thilo Kähne, Hannes Bongartz, Kamil Seyrek, Yuriy Kit, Olga Chinak, Vladimir A. Richter, Olga A. Koval, Inna N. Lavrik

**Affiliations:** 1 Translational Inflammation Research, Medical Faculty, Center of Dynamic Systems (CDS), Otto von Guericke University, 39120 Magdeburg, Germany; max.richter@med.ovgu.de (M.R.); fabian.wohlfromm@med.ovgu.de (F.W.); kamil.seyrek@med.ovgu.de (K.S.); 2 Institute of Experimental Internal Medicine, Medical Faculty, Otto von Guericke University, 39120 Magdeburg, Germany; kaehne@med.ovgu.de; 3 Institute of Biology, Systems Biology, Faculty of Natural Sciences, Otto von Guericke University, 39106 Magdeburg, Germany; bongartz@ovgu.de; 4 Department of Regulation of Cell Proliferation, Institute of Cell Biology, 79005 L’viv, Ukraine; kit@cellbiol.lviv.ua; 5 Department of Biotechnology, Institute of Chemical Biology and Fundamental Medicine, SB RAS, Novosibirsk 630090, Russia; chinakolga@gmail.com (O.C.); richter@niboch.nsc.ru (V.A.R.); o_koval@ngs.ru (O.A.K.)

**Keywords:** breast cancer, mitochondria, κ-Casein, lactaptin, RL2, TOM70, cell death

## Abstract

Breast cancer is still one of the most common cancers for women. Specified therapeutics are indispensable for optimal treatment. In previous studies, it has been shown that RL2, the recombinant fragment of human κ-Casein, induces cell death in breast cancer cells. However, the molecular mechanisms of RL2-induced cell death remain largely unknown. In this study, mechanisms of RL2-induced cell death in breast cancer cells were systematically investigated. In particular, we demonstrate that RL2 induces loss of mitochondrial membrane potential and cellular ATP loss followed by cell death in breast cancer cells. The mass spectrometry-based screen for RL2 interaction partners identified mitochondrial import protein TOM70 as a target of RL2, which was subsequently validated. Further to this, we show that RL2 is targeted to mitochondria after internalization into the cells, where it can also be found in the dimeric form. The importance of TOM70 and RL2 interaction in RL2-induced reduction in ATP levels was validated by siRNA-induced downregulation of TOM70, resulting in the partial rescue of ATP production. Taken together, this study demonstrates that RL2–TOM70 interaction plays a key role in RL2-mediated cell death and targeting this pathway may provide new therapeutic options for treating breast cancer.

## 1. Introduction

Breast cancer is still one of the most common tumorigenic diseases for women. The most common factors associated with breast cancer occurrence are obesity, hormone-associated reproductive factors and hyperplasia of the mammary gland [1]. Surgery, radiation- and chemotherapy remain, until today, the most common approaches for breast cancer treatment. For a more efficient treatment of breast cancer, it is highly necessary to discover novel drugs. To date, various naturally occurring proteins or chemical compounds resulting from pharmacodynamical studies have been used to develop specific drugs for breast cancer [2,3]. Particularly, human milk was found to be a source of multiple bioactive peptides for the treatment of breast cancer. Some of these peptides were recently been utilized to engineer new antitumor drugs such as HAMLET [4], Lactoferrin [5] or Lactaptin [6].

**Figure 1:**
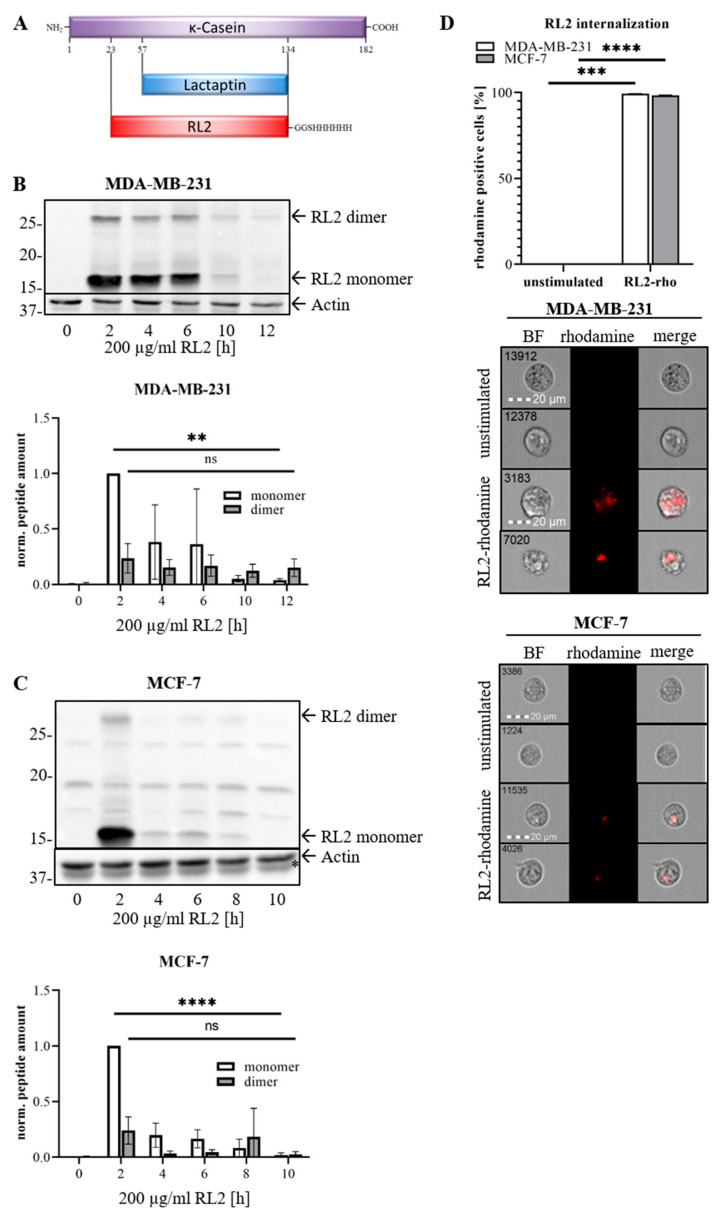
MDA-MB-231 and MCF-7 cells internalize RL2 and degrade it in a time dependent manner. (**A**) Scheme of RL2, Lactaptin and κ-Casein proteins. (**B**) MDA-MB-231 and (**C**) MCF-7 cells were treated with 200 µg/mL RL2 for indicated time intervals. Extracellular RL2 was removed by washing during cell harvest. RL2 was detected in the cellular lysates by Western Blot using anti-κ-Casein antibody. One representative Western Blot out of three independent experiments is shown. Quantification was done for three independent experiments. The statistical analysis was performed by one-way ANOVA test. (**D**) MDA-MB-231 and MCF-7 cells were treated with 50 µg/mL Rhodamine-labelled RL2 for four hours and analyzed with FlowSight^®^ for RL2 internalization. Bottom: At the left column single cells are shown in bright field (BF) channel, at the right column merging of Rhodamine signal and bright field image is performed for representative single cells. Top: The amount of Rhodamine-RL2 positive cells are presented from three independent experiments. The statistical analysis was performed by paired student *t*-test. Relative Western Blot quantifications of Figure 1B,C are shown in Figure S1. ns (not significant; *p* > 0.05), ** (significant; *p* < 0.01), *** (significant; *p* < 0.005), **** (significant; *p* < 0.001).

Lactaptin is a proteolytic fragment of the human milk protein κ-Casein and comprises its amino acids 57 to 134 [6]. The recombinant analogue of Lactaptin RL2 (recombinant Lactaptin 2) is comprised of the amino acids 23–134 of human κ-Casein (Figure 1A). RL2 was shown to induce cell death in MDA-MB-231 and MCF-7 breast cancer cells [6]. Further to this, RL2 suppresses tumor growth and metastasis in mice [7,8,9]. Additionally, it has been reported that RL2 influences the expression of apoptotic proteins and induces autophagy in MDA-MB-231 cells [8]. Besides its tumor-suppressive actions in breast cancer, it was found that RL2 also acts on other cancer cell types such as endometrial cancer, lung cancer and hepatoma cells [6,10]. Importantly, RL2 was shown to spare normal tissue, such as non-malignant mesenchymal stem cells (MSCs) [6]. RL2 has been reported to exist as a mixture of both monomer and dimer forms, the latter are formed via formation of disulfide bonds [11,12]. However, the detailed molecular mechanisms of RL2 interference with cell death machinery are still not known.

There are two ways of apoptosis induction: extrinsic and intrinsic. The extrinsic apoptotic signaling is triggered by ligand binding to the death receptors (DRs), e.g., CD95 (APO-1/Fas) [13,14] or TRAIL-R1/2 [15]. The specific ligand binding to the receptor results in formation of the death inducing signaling complex (DISC) and subsequent activation of the caspase cascade. [15,16,17]. The intrinsic apoptosis pathway is mediated via mitochondria. In particular, mitochondrial outer membrane permeabilization (MOMP) [18] leads to a release of cell death mediators [19,20], activation of effector caspases and apoptosis [21]. The release of other death-inducing factors from mitochondria such as endonuclease G (EndoG) and apoptosis-inducing factor (AIF) might lead to caspase-independent DNA fragmentation and apoptosis.

Another important protein complex for mitochondrial signaling is the translocase of outer membrane (TOM) complex [22]. This complex is closely associated with the translocase of inner membrane (TIM) complex and enables mitochondrial import of proteins [23]. The TOM complex consists of multiple proteins such as TOM20, TOM22, TOM40 and TOM70, playing distinct functions [24,25,26]. TOM20 and TOM22 are receptors recognizing their substrates that are transported via a channel formed by TOM40. The TOM70 receptor recognizes a similar set of substrates as TOM20/TOM22, but is also suggested to have distinct functions [27]. 

In this study, we demonstrate that RL2 induces mitochondrial membrane potential loss, cellular ATP loss and cell death in breast cancer cells. The necrotic morphology of dying cells was observed. Furthermore, we uncovered dimerization processes of RL2 and localized RL2 dimers at mitochondria. The mass spectrometry analysis has further underlined the key role of mitochondria in RL2-induced signaling by identification of potential RL2-targets for cell death mediation including the mitochondrial import protein TOM70. The interaction with TOM70 provides further insights into the connection between RL2 and cell death.

## 2. Results

### 2.1. RL2 Induces ATP Loss and Cell Death in Breast Cancer Cells 

RL2 has been reported to induce cell death in breast cancer cells. To uncover the mechanisms of RL2-induced cell death, RL2-mediated signaling in breast cancer cells was systematically investigated. At the first step, it was analyzed whether RL2 is uptaken by cells over time. Breast carcinoma MDA-MB-231 and MCF-7 cells were treated in a time-dependent manner with 200 µg/mL of RL2. RL2 was detected in the cells shortly after stimulation (Figure 1B,C). Notably, an efficient dimerization of RL2 was observed in MDA-MB-231 cells as well as its time-dependent degradation (Figure 1B; Figure S1). The substantial degradation of RL2 was also observed in MCF-7 cells and was already detected after 4 h (Figure 1C; Figure S1). The dimers assemble via formation of disulfide bridges, and therefore, should mostly diminish after SDS-PAGE under reducing conditions [11]. This is in contrast to the analysis of RL2 via SDS-PAGE under non-reducing conditions, in which the formation of the homodimers can be efficiently detected [6]. Hence, apparently, we observe only a residual amount of RL2 dimers in our experiments. The intracellular localization of RL2 was also observed in single cells using Rhodamine-labeled RL2 [8] and Imaging Flow Cytometry in MDA-MB-231 and MCF-7 cell lines (Figure 1D). Taken together, it was shown that RL2 is internalized into the cells shortly after RL2 administration.

**Figure 2:**
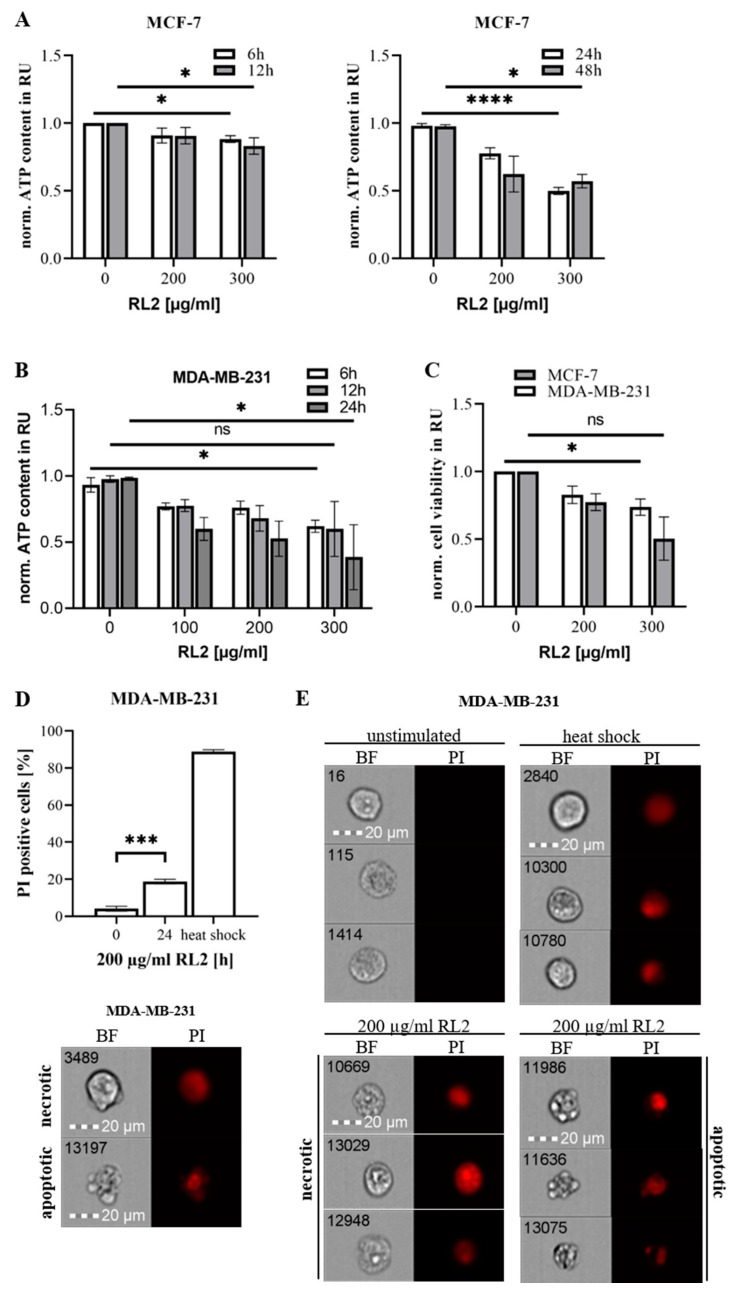
Treatment of RL2 promotes loss of ATP levels and cell death in breast cancer cells. (**A**) MCF-7 and (**B**) MDA-MB-231 cells were treated with indicated concentrations of RL2 for indicated time intervals. Cellular ATP levels were measured using the Cell Titer-Glo^®^-Luminescent Cell Viability Assay. ATP levels are presented in relative units (RU) and normalized to the non-treated cells (norm.). (**C**) MCF-7 and MDA-MB-231 cells were treated with indicated concentrations of RL2 for 24 h. Cell viability was measured using the metabolic RealTime-Glo™ MT Cell Viability Assay. (**A**–**C**) Means and standard deviations are shown for three independent experiments. The statistical analysis was performed by one-way ANOVA test. (**D,E**) MDA-MB-231 cells were treated with 200 µg/mL RL2 for 24 h. Cells were stained with PI and analyzed with FlowSight^®^. (**D**) Top: The amount of PI positive cells from three independent experiments are shown in percentage. The statistical analysis was performed by paired student t-test. Bottom: The morphological features of apoptotic and necrotic cell death are shown exemplarily. Apoptotic cell can be characterized by membrane blebbing, cell shrinkage and nucleus fragmentation, whereas necrotic cell shows enlarged cell body and swollen nucleus. (**E**) Representative pictures of cells are shown. Heat shock treatment serves as positive necrosis control. ns (not significant; *p* > 0.05), * (significant; *p* < 0.05), *** (significant; *p* < 0.005), **** (significant; *p* < 0.001).

To investigate whether RL2 treatment of MDA-MB-231 and MCF-7 cells results in a loss of cell viability, these cells were stimulated in a time- and dose-dependent manner with RL2 followed by measuring total cellular ATP amount (Figure 2A,B). MCF-7 cells showed a marginal reduction in ATP levels at 6 and 12 h after RL2 treatment, but a strong reduction after 24–48 h (Figure 2A). Incubation for 6 and 12 h led to the loss of cellular ATP in MDA-MB-231 cells, which was even more prominent 24 h after RL2 treatment (Figure 2B). Interestingly, MDA-MB-231 cells were more sensitive to RL2-induced loss of ATP compared to MCF-7 cells. Consistent with the drop of ATP levels, the cell viabilities of MCF-7 and MDA-MB-231 cells were reduced after RL2 treatment (Figure 2C). 

These results were in line with the cell death measurements on MDA-MB-231 cells, which were carried out using Propidium Iodide staining and Imaging Flow Cytometry (Figure 2D,E). MDA-MB-231 cells treated for 24 h with RL2 undergo cell death. RL2-treated cells showed a variety of morphologies which could not be correlated to one specific type of cell death (Figure 2E). The dying cells showed features of apoptotic as well as of necrotic cell death. Both, the typical blebbing of apoptotic cells as well as swollen necrotic cells were observed by bright field imaging. The latter had similar morphological features as control cells after heat shock administration. Taken together, it was shown that RL2 is internalized by breast cancer cells and triggers ATP loss and cell death in MCF-7 as well as MDA-MB-231 cells.

### 2.2. RL2 Induces Caspase Activity in Breast Cancer Cells

**Figure 3:**
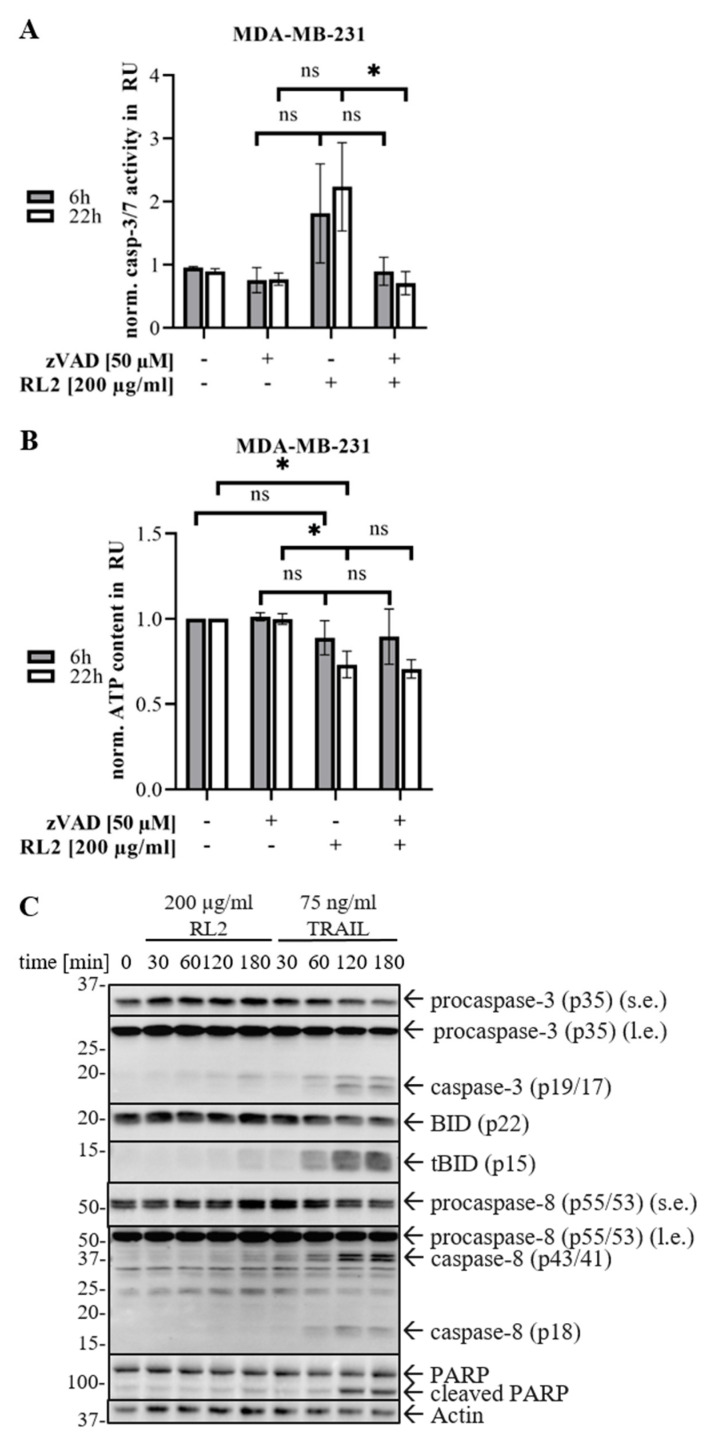
RL2 induces marginal caspase-3 activity and BID processing. (**A**,**B**) MDA-MB-231 cells were pre-treated for one hour with 50 µM pan-caspase inhibitor zVAD-fmk and stimulated with 200 µg/mL RL2 as indicated. (**A**) Caspase-3/-7-activity was determined by Caspase-Glo3/7^®^ Assay. Means and standard deviations of caspase activity are shown in relative units (RU) for three independent experiments and statistically analyzed by paired student t-test. (**B**) ATP levels were measured using the Cell Titer-Glo^®^-Luminescent Cell Viability Assay. Mean and standard deviation are shown for three independent experiments. The statistical analysis was carried out by paired student *t*-test. (**C**) MDA-MB-231 cells were stimulated with 200 µg/mL RL2 or 75 ng/mL TRAIL (positive control) for indicated periods of time, and subjected to Western Blot analysis with the indicated antibodies. One representative Western Blot out of three independent experiments along with its quantification is shown in Figure S2. ns (not significant; *p* > 0.05), * (significant; *p* < 0.05).

To identify whether the cell death induced by RL2 indeed has apoptotic features, caspase-3/7 activity assays were carried out. RL2 treatment induces minor caspase-3/7 activity in MDA-MB-231 cells, which was completely blocked by pan-caspase inhibitor zVAD-fmk (Figure 3A). Interestingly, the strength of caspase-3/7 activation did not change over time and remained nearly constant from six to 22 h after RL2 treatment (Figure 3A). A dose-dependent analysis of caspase activity after RL2 stimulation also did not reveal a stronger increase in the amount of caspase-3/7 activity upon the increase in RL2 concentration. Interestingly, the treatment with pan-caspase inhibitor zVAD-fmk could not rescue the ATP loss after RL2 stimulation, indicating that this ATP drop induced by RL2 treatment was not dependent on caspase-3/7 activity (Figure 3B).

The results of Western Blot analysis of caspase cleavage in MDA-MB-231 cells were consistent with the results of the caspase-3/7 activity assays. Even though the first cleavage of caspase-3 was monitored already one hour after RL2 stimulation, the amount of caspase-3 cleavage products did not strongly increase over time (Figure 3C; Figure S2). This was also in accordance with the weak increase in caspase-3/7 activity. Only a slight increase in the amount of p19/p17 was observed three hours after stimulation. Accordingly, a rather weak cleavage of caspase-8 was detected with induced processing to p43/p41, but not p18, after three hours of treatment. The processing of Bid was also detected three hours after RL2 stimulation. To compare the strength of caspase activation upon RL2 treatment with a well-established apoptosis induction, the stimulation of MDA-MB-231 cells with 75 ng/mL TRAIL was used (Figure 3C; Figure S2). Interestingly, in TRAIL-stimulated cells, a much more prominent level of caspase-3 processing was observed at earlier time points compared to RL2-stimulated cells. Accordingly, a stronger cleavage of caspase substrates Bid and PARP was detected after TRAIL as compared to RL2-stimulated cells. Moreover, the amount of TRAIL-induced caspase cleavage products increased over time.

Taken together, the analysis of caspase activity shows that RL2 induces caspase-3/7 activation in breast cancer cells. However, the loss of ATP levels observed in Figure 3B is not dependent on caspase-3/7 activity. Additionally, in comparison to the induction of the extrinsic apoptotic pathway by TRAIL, the activation strength of effector caspases upon RL2 treatment seems to be relatively weaker.

### 2.3. RL2 Interacts with the Mitochondrial Import Protein TOM70

**Figure 4:**
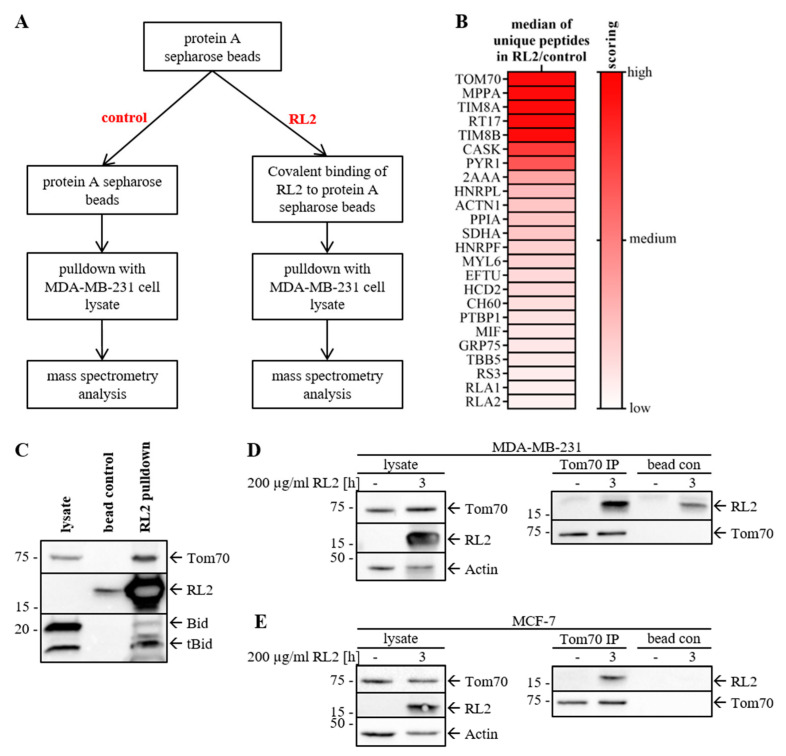
RL2 interacts with mitochondrial membrane protein TOM70. (**A**) Workflow for RL2-based mass spectrometry analysis. MDA-MB-231 cell lysates were incubated with RL2-coupled Protein A Sepharose beads (RL2-pulldown) or Protein A Sepharose beads (control). Precipitated proteins were identified by nanoLC-tandem mass spectrometry. (**B**) Mass spectrometry identified unique peptides for each protein. The median of the unique peptides from three independent experiments is shown for each protein and compared for RL2- and control pulldown. The scoring indicates the increase in unique peptides identified. (**C**) RL2-pulldown precipitates were analyzed by Western Blot and probed for indicated proteins. (**D**) MDA-MB-231 and (**E**) MCF-7 cells were treated with 200 µg/mL RL2 as indicated. TOM70 immunoprecipitation (TOM70 IP) was performed with TOMM70A antibody or bead-only control and probed for indicated proteins by Western Blot. One representative experiment out of three is shown for all Western Blots and immunoprecipitations are shown in Figure S3.

To identify the mediators of RL2-induced cell death, mass spectrometry analysis of the RL2 interactome was carried out in MDA-MB-231 cells. This was performed by immobilization of RL2 on protein A conjugated Sepharose beads followed by RL2-pulldown from MDA-MB-231 cell lysates and subsequent mass spectrometry analysis (‘RL2′-axis (right), Figure 4A). To control the specificity of RL2-pull-down, lysates from MDA-MB-231 cells were incubated with protein A Sepharose beads without prior RL2 immobilization and subsequently analyzed by mass spectrometry (‘control’, Figure 4A). Thereby, we could exclude unspecific off-target hits and search for the most prominent interactors of RL2 in vitro.

The mass spectrometry screen with RL2-pulldown reproducibly revealed the mitochondrial import protein TOM70 as one of the most prominent hits (Figure 4B, Table S1). It was detected in RL2-pulldown with high scoring in three independent experiments. Furthermore, it was not found to be associated with the protein A Sepharose beads in the control pulldown in three independent experiments. Tubulin (TBB5, Figure 4B) and actin (ACTN1, Figure 4B), which have already been identified as interaction partners of RL2 in the previous studies [8], were also detected in this analysis with a relatively high score, indicating the correct performance of the entire pull-down assay. Other hits were also present both in the RL2 and control pulldowns, but in a lower abundance concluded by a lower number of identified unique peptides. Hence, we have concluded that TOM70 presents a promising hit in the mass spectrometry screening assay. Furthermore, an increased number of mitochondrial proteins were identified in the RL2-pulldown (Table S2). This list comprises other proteins of mitochondrial import/export such as TOM22, TIM8A and TIM8B detected by mass spectrometry, albeit with a similar or lower scoring than TOM70 (Figure 4B, Tables S1 and S2).

The proteomics screening was followed by validation of the interaction of TOM70 with RL2 via pulldown assays in combination with Western Blot analysis. The interaction of RL2 with TOM70 was confirmed in MDA-MB-231 cells (Figure 4C; Figure S3A). Furthermore, Bcl-2 family protein members present at the mitochondria such as tBid were detected in RL2-pulldown (Figure 4C). Finally, the RL2–TOM70 interaction was validated by the so-called reverse approach of TOM70 immunoprecipitation from RL2 treated MDA-MB-231 or MCF-7 cells (Figure 4D,E; Figure S3B,C). These results provide strong evidence on RL2–TOM70 interaction and indicate that RL2 is targeted to the mitochondria after penetration into the cell.

### 2.4. RL2 Is Targeted to the Mitochondria and Induces Loss of Mitochondrial Membrane Potential

**Figure 5:**
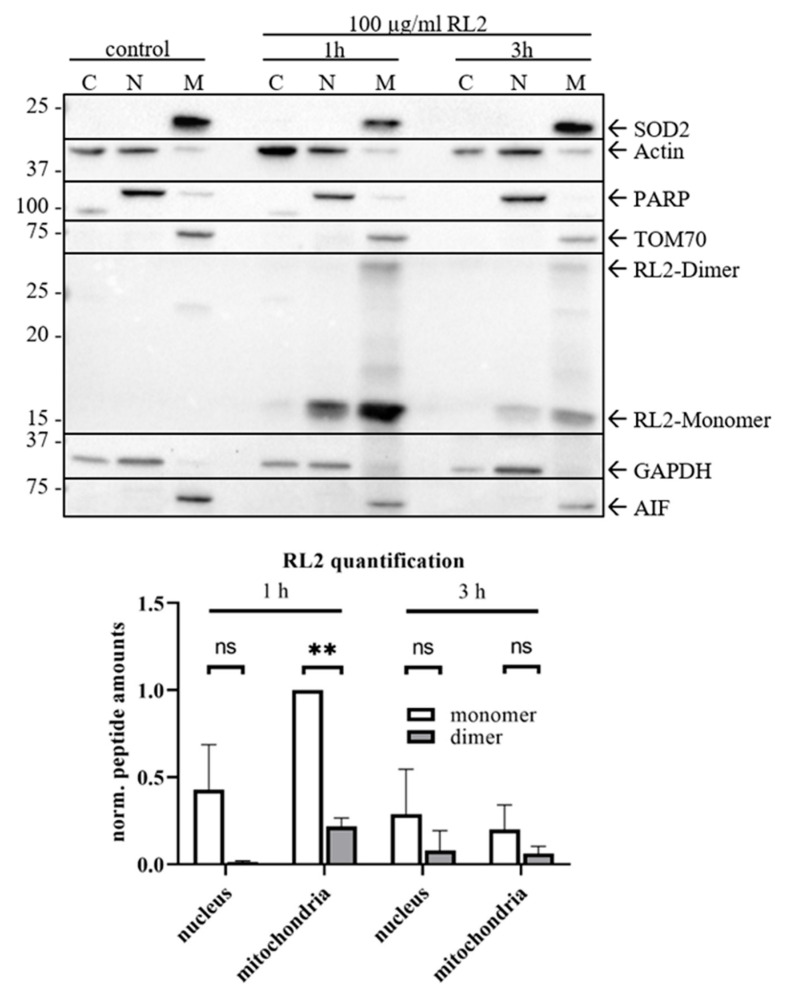
Internalized RL2 is localized at mitochondria. MDA-MB-231 cells were stimulated with 100 µg/mL RL2 for indicated time points. Cellular fractionation for cytoplasm (‘C’), nucleus (‘N’) and mitochondria (‘M’) was performed. The fractions were subjected to Western Blot analysis with indicated antibodies. SOD2, Actin, GAPDH and PARP were used as fraction control. The bands corresponding to RL2-Monomer and -Dimer were quantified by ImageLab 5.1 beta (Bio-Rad) from three independent experiments. The statistical analysis was performed by paired student *t*-test (bottom panel). One representative Western Blot out of three is shown in Figure S4. ns (not significant; *p* > 0.05), ** (significant; *p* < 0.01).

To consider possible RL2 localization within the cell, we have analyzed its properties and identified RL2 as a positively charged peptide. It has a pI value of 9.85 and a hydrophobicity of −0.9, which was calculated with the ExPASy ProtParam tool. Hence, it was suggested that it might be targeted to the mitochondrial membrane, and thereby, causes events leading to alterations in ATP production and cell death. To check this hypothesis, a cellular fractionation of RL2-treated MDA-MB-231 cells was carried out. RL2 was prominently detected in the mitochondrial fraction along with the mitochondrial marker SOD2 as well as TOM70 (Figure 5; Figure S4). Remarkably, the dimers of RL2 in the mitochondrial fraction were also observed. Interestingly, RL2-mediated release of caspase-independent mediators of cell death from mitochondria like AIF was not observed upon RL2 treatment (Figure 5; Figure S4). This indicates that these factors might not be involved in RL2-mediated cell death.

We have demonstrated that RL2 treatment causes ATP-loss followed by cell death (Figure 1). Because mitochondria are the main source of ATP production, it was suggested that RL2 translocation to mitochondria alters the mitochondrial membrane potential, and thereby, the function of the respiratory chain followed by the loss of ATP production and cell death.

**Figure 6:**
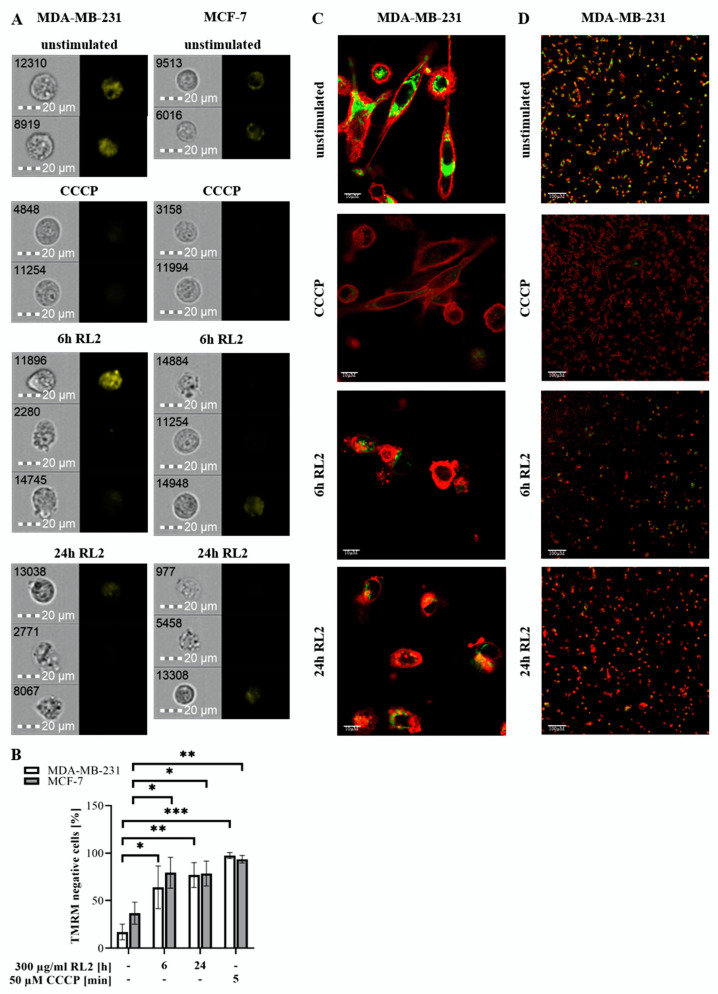
RL2 induces loss of mitochondrial membrane potential. (**A**–**D**) MDA-MB-231 and MCF-7 cells were stimulated with 300 µg/mL RL2 for 6 and 24 h. Cells were subsequently stained with 20 µM TMRM. The reduced TMRM signal indicated the loss of mitochondrial membrane potential. CCCP treatment served as positive control for TMRM reduction. (**A**) Cells were analyzed with FlowSight^®^ for TMRM-signal. Representative cells from three independent experiments are shown. (**B**) Mean and standard deviations for the amounts of TMRM negative cells (in %) from three independent experiments are shown. The statistical analysis was performed by paired student t-tests. (**C**,**D**) RL2-treated MDA-MB-231 cells were stained with 5 mg/mL cell membrane stain (CellMask™ Deep Red Plasma membrane Stain, C10046, Thermo Fisher, Walham, MA, USA) and introduced to confocal laser scanning microscopy. Membrane- and TMRM-stained cells are shown in merge for single cells (**C**, scale: 10 µM) and cell populations (**D**, scale: 100 µM). The red color corresponds to the plasma membrane staining. The green (**C**) and yellow (**A**,**B**) colors correspond to TMRM staining. * (significant; *p* < 0.05), ** (significant; *p* < 0.01), *** (significant; *p* < 0.005).

Hence, mitochondrial membrane potential was analyzed by quantitative single cell analysis of MDA-MB-231 and MCF-7 cells using our FlowSight^TM^ imaging flow cytometer (Figure 6A,B). Both cell lines were treated for 6 or 24 h with RL2 and then subjected to mitochondrial TMRM staining. Reduced TMRM signals, observed in MDA-MB-231 and MCF-7 cells treated with RL2, correlate with loss of mitochondrial membrane potential in these cells upon RL2 treatment. CCCP treatment was used as a positive control for the loss of mitochondrial potential (Figure 6A,B). Additionally, MDA-MB-231 cells were analyzed by confocal laser scanning microscopy using CellMask^TM^ Deep red plasma membrane staining dye and mitochondrial TMRM staining. Cells positive for TMRM and plasma membrane staining were analyzed for correlation of both fluorescence signals. Here, loss of TMRM intensity was also observed upon 6 or 24 h of RL2 treatment (Figure 6C,D; Figure S5A–E), confirming the results observed with imaging Flow Cytometry assay in Figure 6A. Taken together, these results demonstrate that RL2 treatment induces loss of mitochondrial membrane potential in MDA-MB-231 and MCF-7 cells (Figure 6A–D; Figure S5A–E).

### 2.5. TOM70 Downregulation Partially Rescues RL2-Induced ATP Loss

**Figure 7:**
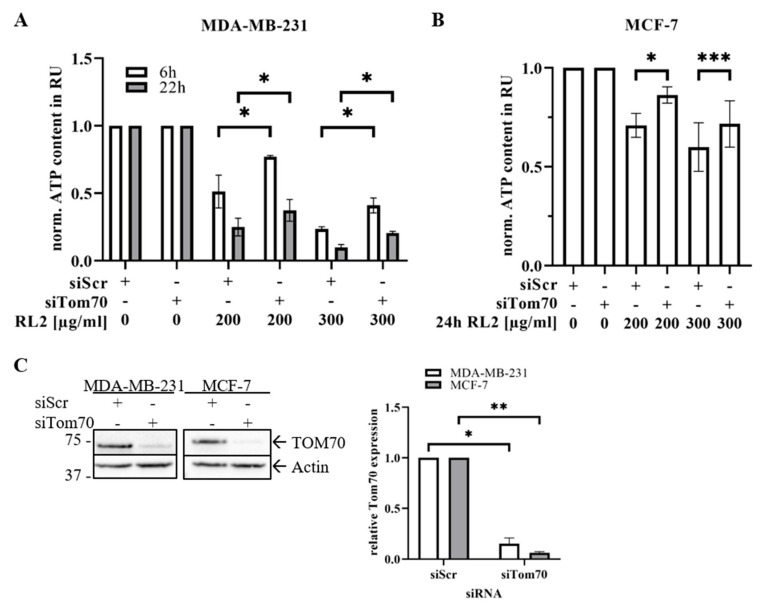
TOM70 knock-down rescues RL2-induced drop of ATP levels in MDA-MB-231 and MCF-7 cells. (**A**) MDA-MB-231 and (**B**) MCF-7 cells were transfected with TOM70 siRNA (#AM16708, Thermo Fisher) or scrambled control siRNA. Subsequently, cells were stimulated for indicated times and concentrations of RL2. ATP levels were measured using the Cell Titer-Glo^®^-Luminescent Cell Viability Assay. Means and standard deviations are shown for three independent experiments. The statistical analysis was performed using paired student *t*-test. (**C**) The transfection efficiency for TOM70 downregulation was controlled by Western Blot analysis (left). Protein quantification was performed with ImageLab 5.1 beta (Bio-Rad) for three independent experiments. Actin was used as a loading control. The statistical analysis was performed by paired student *t*-test (right). Relative Western Blot quantifications of Figure 7C are shown in Figure S6. * (significant; *p* < 0.05), ** (significant; *p* < 0.01), *** (significant; *p* < 0.005).

Translocation of RL2 to mitochondria and the impact on the mitochondrial membrane potential supported the role of mitochondria in RL2-induced cell death and the interaction with TOM70 at the mitochondrial membrane. Moreover, the TOM complex has been reported to be crucially involved in ATP production and the function of the respiratory chain as a main macromolecular transport complex at the mitochondrial membrane. To delineate the role of TOM70 in RL2-mediated ATP loss, TOM70 was downregulated in MDA-MB-231 and MCF-7 cells using siRNA (Figure 7C; Figure S6). This was followed by measuring the ATP levels of RL2-treated cells. Importantly, the downregulation of TOM70 partially rescued RL2-induced ATP loss, underlining the importance of TOM70 in RL2-mediated cell death (Figure 7A,B). Taken together, these data indicate that RL2 targets TOM70 at the mitochondria, which might block the function of the TOM complex, leading to the impairment of ATP production that is followed by the demolition of the cell.

## 3. Discussion

Contemporary anticancer research strongly requires the establishment of antitumor therapies that are specific for a particular cancer type. In this regard, bioactive peptides, obtained from human milk, are promising antitumor therapeutics for the treatment of breast cancer. One of these peptides is Lactaptin, which is a fragment of proteolytically cleaved κ-Casein [6]. Previous studies have shown that Lactaptin and its recombinant analogue RL2 have strong antitumor effects in breast cancer, endometrial cancer, lung cancer and hepatoma cells [6,10]. Interestingly, there is strong evidence that RL2 specifically affects cancer cells and does not exert any suppressive action on normal cells or non-malignant mesenchymal stem cells [6]. Besides the clinical implications of RL2 in cancer treatment, the molecular mechanisms of RL2-induced antitumor effects remain elusive. Here, further insights into RL2-mediated cell death signaling were delineated, which plays an indispensable role for the follow-up clinical development.

Efficient internalization of RL2 by cancer cells provides the essential prerequisite for its action [28]. The positively charged RL2 has a pI value of 9.85 and a hydrophobicity of −0.9, enabling RL2 to pass the plasma membrane of the cells [11,12]. Due to these properties, there seems to be no need for a specific RL2 receptor or an active transport through the cell membrane. Another key feature for the efficient action of the anticancer drug is its bioavailability. Interestingly, it was observed that the degradation of RL2 occurs four to eight hours following the RL2 penetration into the cells. The potential of cell death induction by RL2 could be limited to its relatively short half-life (Figure 1). This is corroborated by the observation that RL2 degradation in MCF-7 cells occurs faster than in MDA-MB-231 cells. Faster RL2 degradation in MCF-7 cells correlates with less ATP downmodulation compared to MDA-MB-231 cells. Thus, reduced bioavailability of RL2 might be the explanation for reduced loss of ATP and cell death of MCF-7 cells. Hence, increasing the bioavailability of RL2 in vivo is an approach which could further enhance the RL2-induced cell death of cancer cells [29,30].

In accordance with recent reports, we have observed the dimerization of RL2 in breast cancer cells [11]. In addition, we have found out that RL2 co-localizes with mitochondria in breast cancer cells. The detection of the RL2 dimers at the mitochondria allows us to suggest that dimers of RL2 might have a higher efficiency for binding to TOM70. Subsequently, one can suggest that the dimerization of RL2 might play a crucial role in its binding to TOM70 and cell death induction.

**Figure 8:**
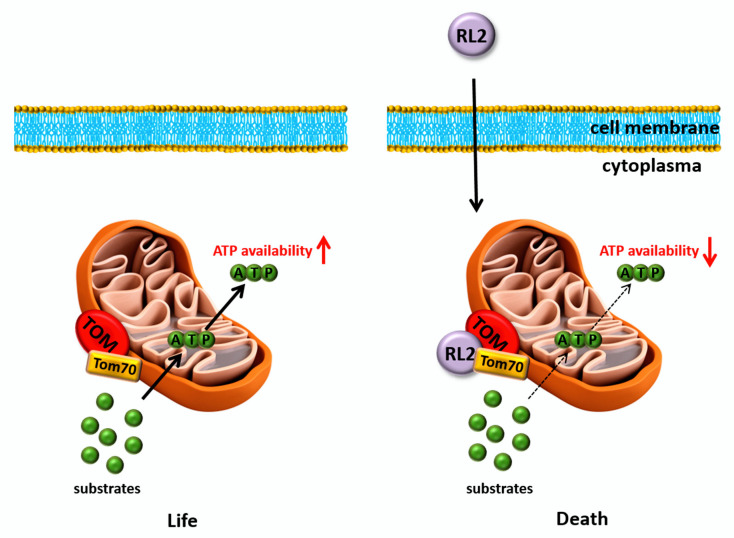
RL2-mediated cell death pathways. RL2 translocates into the cytosol and localizes at the mitochondrial membrane by interaction with TOM70. This interaction leads to a drop of mitochondrial membrane potential and reduces ATP production. The latter might be due to the impairment of the TOM complex function, which is required for the transport of the substrates for ATP synthesis or conformational changes at the outer mitochondrial membrane caused by RL2–TOM70 interaction. The reduced ATP levels lead to cell death.

According to our results, RL2 is directly targeted to mitochondria. We uncovered that RL2 is localized to the mitochondria by cellular fractionation of MDA-MB-231 cells and further identified mitochondrial outer membrane protein TOM70 as a major target of RL2 (Figure 8). TOM70 was first identified as a receptor and a regulator of transmembrane transit for the precursor of the ADP/ATP carrier (AAC) [22,31,32]. Moreover, we have shown that RL2 treatment induces the loss of mitochondrial membrane potential and thereby might lead to the decreased ATP production, reduced cell viability and increased cell death (Figure 8). The mitochondrial localization of RL2 is consistent with its biochemical properties and previous reports on RL2-mediated MOMP induction in HA-1 hepatoma cells [7]. Additionally, it has been reported that TOM70 serves as a link for Ca2+ transfer from ER to the mitochondria by recruiting IP3 receptors to mitochondria [33]. This leads to inhibition of mitochondrial respiration, induction of autophagy, and inhibition of cell proliferation. Therefore, it might be suggested that RL2 binding to TOM70 impairs Ca2+ transfer between ER and mitochondria, that contributes to cell death [33]. The downregulation of TOM70 rescued the RL2-induced reduction in ATP levels, indicating that TOM70 is one of the potential key players in RL2-induced ATP loss. The ATP levels of unstimulated cells after TOM70 downregulation were not affected (Figure 7). This could be explained by the fact, that siRNA based TOM70 downregulation does not completely block protein expression, and therefore, minor levels of TOM70 remain. TOM complex could potentially function normally without TOM70, which is not a part of the TOM complex core [34]. This and the functional analysis of the RL2–TOM-complex interaction remain unclear and could be further addressed in future studies.

Our results provide evidence for and against the involvement of intrinsic apoptosis in RL2-mediated cell death [18,19]. TMRM loss cannot be considered as a direct readout of MOMP. Moreover, we observe rather weak caspase-3/7 activity after RL2 treatment as well as procaspase-3 processing, mostly to caspase-3 cleavage product p19. The latter is a precursor of the active p17 subunit, which indicates the full maturation of caspase-3 and serves as a marker of caspase-3 activation in apoptosis [21]. Moreover, the substrates of caspase-3, PARP1 and BID, were only marginally processed upon RL2 treatment, which differs from the signaling pattern observed upon intrinsic apoptosis induction [21]. Taken together, these results indicate very little contribution of caspase activity to RL2-induced cell death. Moreover, the experiments with zVAD-fmk indicate that RL2 can act in a caspase-independent manner. Considering that RL2 treatment might lead to cell death independent of caspase activity, which is accompanied by the loss of MMP and ATP levels, it might be suggested that RL2 induces one of the programs of regulated necrosis, such as MPT-induced or energy crisis-induced necrosis [35]. Interestingly, imaging flow cytometry has revealed the evidence for both types of cell death: apoptosis and necrosis. In particular, a high number of cells with necrotic morphologies as well as the presence of apoptotic cells after RL2 treatment were observed (Figure 2; Figure 6). Thus, the mechanisms of caspase activity induction, as well as the crosstalk of apoptotic and necrotic cell death, upon RL2 administration, have to be addressed in future studies.

Our findings indicate that RL2 treatment downmodulates the intracellular ATP production, possibly leading to the metabolic reprogramming of the cells. Tumor cells have different metabolic properties [35,36] and, accordingly, this might explain the different sensitivity of cancer cells to RL2. Likewise, in this study, we used two breast cancer cell lines: a highly metastatic MDA-MB-231 and a non-metastatic MCF7. The MDA-MB-231 were more sensitive to RL2 treatment compared to MCF-7, which might be explained by their different metabolic status. In this regard, RL2 might be used as a sensitive tool to uncover the metabolic status of a particular cancer cell line in future studies.

Furthermore, it would be very interesting to investigate whether a combinatorial treatment based on RL2 in combination with other chemotherapeutics would have a clinical implication and has advantages over conventional breast cancer therapeutics. Moreover, as we have shown that RL2 leads to the metabolic reprogramming of the cancer cells via reducing the ATP production, its applications in low concentrations in combinatorial treatments might provide further therapeutic benefits [35,36].

Taken together, we have identified TOM70 as one major target of RL2 at the mitochondrial membrane and suggested that RL2 specifically acts at the mitochondria, leading to the drop of ATP production and cell death (Figure 8). Our results provide new perspectives in targeting mitochondria in breast cancer development and progression via a new RL2-based anticancer treatment.

## 4. Material and Methods 

### 4.1. Antibodies and Reagents

All chemicals were of analytical grade and purchased from AppliChem (Darmstadt, Germany), Carl Roth (Karlsruhe, Germany), Merck (Darmstadt, Germany) or Sigma-Aldrich (Taufkirchen, Germany). RL2 was purified as described previously [6] and applied to cells in 137 mM NaCl. Z-VAD-FMK (N-1510, Bachem, Bubendorf, Switzerland) and recombinant TRAIL (KillerTRAIL^™^, Enzo Life Sciences, Farmingdale, NY, USA) were applied to cells in indicated concentrations. The following antibodies were used for Western Blot analysis: polyclonal anti-AIF antibody (#5318), polyclonal anti-BID antibody (#2002), polyclonal anti-caspase-3 antibody (#9662), polyclonal anti-PARP antibody (#9542), and polyclonal anti-SOD2 antibody (#13194) from Cell Signaling Technology, Danvers, MA, USA); polyclonal anti-actin antibody (A2103, Sigma-Aldrich, St Louis, MO, USA); polyclonal anti-κ-Casein antibody (#ab111406, Abcam, Cambridge, United Kingdom); monoclonal anti-TOMM70A antibody (sc-390545n Santa Cruz Biotechnology, Dallas, TX, USA); and monoclonal anti-caspase-8 antibody (kindly provided by Prof. P. H. Krammer, DKFZ, Heidelberg, Germany). Horseradish peroxidase-conjugated goat anti-mouse IgG1, IgG2b, goat anti-rabbit and rabbit anti-goat were from Santa Cruz (CA, USA).

### 4.2. Cell Culture

Human adenocarcinoma cells MDA-MB-231 (purchased: #ACC 732, DSMZ, Braunschweig, Germany) were maintained in Leibovitz L15 media (Gibco^TM^), supplemented with 10% heat-inactivated fetal calf serum and 1% Penicillin-Streptomycin. Human adenocarcinoma cells MCF-7 (purchased: #ACC 115, DSMZ, Braunschweig, Germany) were maintained in RPMI 1640 Phenol Red (Thermo Fisher Scientific Inc., Waltham, MA, USA), supplemented with 10% heat-inactivated fetal calf serum, 1% Penicillin-Streptomycin, 1 mM sodium pyruvate and 1 x MEM non-essential amino acids in 5% CO_2_.

### 4.3. ATP Measurement Assay 

1.2 × 10^4^ (MDA-MB-231) or 2 × 10^4^ (MCF-7) cells were seeded in 96-well plates. Cells were stimulated in a volume of 50 µL. Measurements were performed according to the manufacturer’s instructions (CellTiter-Glo^®^ Luminescent Cell Viability Assay, Promega, Germany) with the addition of 50 µL CellTiter-Glo^®^ solution to each well. The luminescence intensity was analyzed in duplicate using the microplate reader Infinite M200pro (Tecan, Männedorf, Switzerland). The values were normalized against the viability of untreated cells, which was set as one relative unit (RU).

### 4.4. Cell Viability Measurements by Metabolic (MTT) Assay

In total, 1.2 × 10^4^ (MDA-MB-231) or 2 × 10^4^ (MCF-7) cells were seeded in 96-well plates. Cells were stimulated in a volume of 100 µL mastermix including 1× concentrated ‘MT Cell Viability Substrate’ and 1× concentrated NanoLuc^®^ luciferase. Measurements were performed according to the manufacturer’s instructions (RealTime-Glo™ MT Cell Viability Assay, Promega, Germany). The luminescence intensity was analyzed in duplicate using the microplate reader Infinite M200pro (Tecan, Männedorf, Switzerland). The values were normalized against the viability of untreated cells that was set as one relative unit (RU).

### 4.5. Caspase-3/7 Activity Assay

In total, 1.2 × 10^4^ MDA-MB-231 cells were seeded in 96-well plates. Cells were stimulated in a volume of 50 µL. Measurements were performed according to the manufacturer’s instructions (Caspase-Glo^®^ 3/7 Assay, PromegaMadison, WI, USA) with addition of 50 µL of the Caspase-Glo^®^ 3/7 solution to each well. The luminescence intensity was analyzed in duplicate by the microplate reader Infinite M200pro (Tecan, Männedorf, Switzerland). The values were normalized against caspase activity of non-treated cells and set as one relative unit (RU).

### 4.6. Cell Death and Internalization Measurements by Imaging Flow Cytometry

Analysis of RL2 internalization and cell death induction was performed with FlowSight^®^ Imaging Flow Cytometer (Amnis/Merck Millipore, Darmstadt, Germany). MDA-MB-231 or MCF-7 cells were treated with RL2 or Rhodamine-labelled RL2 [8] for measuring cell death or internalization, respectively. Samples for measuring cell death were additionally stained with propidium iodide (PI). The samples were excited with a 488 nm laser. Emission was detected in channel 4 and bright field images were acquired in channels 1 and 9. For every sample, 10,000 events were recorded. Data were analyzed with IDEAS software version 6.2 (Amnis/Merck Millipore, Darmstadt, Germany). For internalization, Rhodamine-positive cells were taken as RL2-positive cells. RL2-induced cell death was calculated via the percentage of PI positive cells.

### 4.7. Western Blot Analysis and Immunoprecipitation

In total, 1.25 to 2.5 × 10^5^ cells were seeded in 6-well plates. Cells were harvested, washed with PBS and lysed for 30 min on ice in lysis buffer (20 mM Tris HCl, Ph 7.4, 137 mM NaCl, 2 mM EDTA, 10% glycerine, 1% Triton X-100, Protease Inhibitor mix (Roche, Mannheim, Germany)) and subjected to Western blot analysis. SDS-PAGE was performed with 12% SDS gels. The TransBlot Turbo system (Biorad, Hercules, CA, USA) was used to blot the gels to nitrocellulose membranes. Blots were blocked in 5% non-fat dried milk (SantaCruz, Dallas, TX, USA) in PBS with 0.05% Tween 20 for one hour. Washing steps were performed with PBS-Tween threefold for 5 min. Incubation with primary antibodies was performed overnight at 4 °C in PBS-T. HRP-coupled isotype-specific secondary antibodies were incubated for 1 h at room temperature in 5% non-fat dried milk. Chemiluminescence signal was produced with Luminata Forte (Merck Millipore, Darmstadt, Germany) and detected with a ChemiDoc imaging system (Biorad, Hercules, CA, USA). TOM70 immunoprecipitation (IP) was performed with MDA-MB-231 and MCF-7 cells after RL2 stimulation. Stimulation was stopped by adding 10 mL cold PBS and scrubbing of the cells. Cells were centrifuged for 5 min at 500× *g* and washed once with cold PBS. Cells were lysed in 500 µL lysis buffer for 30 min on ice and subsequently centrifuged for 15 min at 14,600× *g*. An amount of 50 µL supernatant was used as input control. The remaining supernatant of all samples was adjusted to the same protein concentration and used for immunoprecipitation. TOM70 was immunoprecipitated by mixing 10 µL protein A Sepharose (GE Healthcare, Chicago, IL, USA) and anti-TOM70 antibody (Abcam #ab89624). TOM70 IP samples were rotated overnight at 4 °C, washed three times with lysis buffer and once with PBS. Samples were subjected to Western Blot analysis.

### 4.8. Cellular Fractionation

In total, 2.5 × 10^6^ MDA-MB-231 cells were stimulated with RL2 or left untreated. Subsequently, cells were trypsinated and washed in PBS. All centrifugation steps in the following fractionation were performed at 17,000× *g*. A swelling step was performed in swelling buffer (10 mM HEPES Ph 7.6, 10 mM KCl, 2mM MgCl_2_, 0.1 mM EDTA, Protease Inhibitor mix) for 5 min followed by addition of 0.3% NP-40 (Thermo Fisher Scientific Inc., Waltham, MA, USA) for 1 min. Centrifugation for 1 min resulted in a separation of the cytoplasmic fraction. The remaining pellet was resuspended in 500 µL swelling buffer and centrifuged for 15 s. The pellet was incubated for 30 min with 40 µL ‘nucleus buffer’ (50 mM HEPES pH 7.8, 50 mM KCl, 300 mM NaCl, 0.1 mM EDTA, 10% Glycerol, Protease Inhibitor mix), resuspended every 10 min and centrifuged for 5 min. The nuclei-containing supernatant and mitochondria-containing pellet were separated, and the pellet was washed twice with PBS. The samples were subjected to Western blot analysis.

### 4.9. Protein Knock-Down

Silencing of TOM70 was performed with TOM70 siRNA (#AM16708, Thermo Fisher Scientific Inc., Waltham, MA, USA) and AllStars Negative Control siRNA (SI03650318, Qiagen, Hilden, Germany). A total of 1.25 × 10^5^ cells was seeded in antibiotic-free medium in 6-well plates and transfected with DharmaFECT1 transfection reagent (T-2001, Dharmacon, Lafayette, CO, USA). Medium was changed after 24 h. Then, 48 h after, the transfection cells were used for Western Blot or cell viability assay analysis.

### 4.10. Protein Pull-Down and Mass Spectrometry Analysis

Analysis of RL2-binding partners was performed by protein pull-down. RL2 was covalently coupled to sepharose beads using Pierce™ Co-Immunoprecipitation Kit according to the manufacturer’s instructions (Thermo Fisher Scientific Inc., Waltham, MA, USA) and incubated with total cell lysates of MDA-MB-231 cells. The precipitates were analyzed by nanoLC- tandem mass spectrometry and Western Blot. 

Sample preparation for mass spectrometry was performed via on-beads digestion. In brief, beads were rehydrated in 50 mM NH_4_HCO_3_, pH 8.0, and subsequently incubated with 1 mM DTT at 56 °C for 45 min. Afterwards, reduced cysteine residues were β-methylthiolated via the addition of 5 mM methyl methanethiosulfonate at room temperature for 30 min. Proteins were digested by adding 0.5 μg trypsin (TrypsinGold, Promega, Madison, WI, USA) and incubated at 37 °C overnight. The generated tryptic peptides were eluted from the beads with two washing steps using 50 μL of 25 mM NH_4_HCO_3_ for each wash. The washes corresponding to one sample were pooled together and dried in a vacuum centrifuge. The peptides were dissolved in 5 μL 0.1% trifluoroacetic acid (TFA) and purified on ZIP-TIP, C18-nanocolumns (Millipore, Billerica, MA, USA). The peptides were eluted in 7 μL of 70% (*v*/*v*) acetonitrile (ACN) and subsequently dried in a vacuum centrifuge. LC-MS/MS was performed on a hybrid dual pressure linear ion trap/orbitrap mass spectrometer (LTQ Orbitrap Velos Pro, Thermo Scientific, San Jose, CA, USA) equipped with an EASY-nLC Ultra HPLC (Thermo Scientific, San Jose, CA, USA). The peptide samples were dissolved in 10 μL of 2% ACN/0.1% TFA and fractionated on a 75 μm I.D., 25 cm PepMap C18-column, packed with 2 μm resin (Dionex, Germany). Separation was achieved by applying a gradient from 2% ACN to 35% ACN in 0.1% formic acid (FA) over a 150 min gradient at a flow rate of 300 nL/min. The LTQ Orbitrap Velos Pro MS exclusively used CID-fragmentation when acquiring MS/MS spectra, consisting of an orbitrap full MS scan followed by up to 15 LTQ MS/MS experiments (TOP15) on the most abundant ions detected in the full MS scan. The essential MS settings were as follows: full MS (FTMS; resolution 60,000; m/z range 400–2000); MS/MS (Linear Trap; minimum signal threshold 500; isolation width 2 Da; dynamic exclusion time setting 30 s; singly charged ions were excluded from selection). Normalized collision energy was set to 35%, and the activation time was set to 10 ms. Raw data processing and protein identification of the high resolution orbitrap datasets were performed with de novo sequencing algorithms of PEAKS Studio 8.0 (Bioinformatics Solutions Inc.,Waterloo, ON, Canada). The false discovery rate was set to <1%.

### 4.11. Mitochondrial Membrane Potential Analysis by Confocal Laser Scanning Microscopy and Imaging Flow Cytometry

A total of 1 × 10^6^ MDA-MB-231 cells was seeded on poly-L-lysine-coated glass cover slips and cultivated for 24 h. Subsequently, cells were stimulated with 300 µg/mL RL2 for 6 or 24 h. As the positive control for TMRM indicated mitochondrial membrane potential loss, the cells were stimulated with CCCP (Carbonyl cyanide-*m*-chlorophenyl hydrazone) for 5 min. Sequentially, cells were stained with 20 µM TMRM (Tetramethylrhodamine-methyl ester) (MitoProbe™ TMRM Kit for Flow Cytometry, M20036, Invitrogen, Carlsbad, CA, USA) and 5 mg/mL cell membrane stain (CellMask™ Deep Red Plasma membrane Stain, C10046, Thermo Fisher). Afterwards, cells were placed into the pre-heated incubation chamber of the laser scanning microscope and left for 30 min. Imaging was performed with a confocal laser scanning microscope (LSM700, Zeiss, Jena, Germany). The temperature of the incubation chamber (Pecon, Erbach, Germany) and of the microscope’s objective was adjusted to 37 °C. Images of TMRM fluorescence were obtained using a 63× objective lens with an excitation at 555 nm and an emission of 585–620 nm. Cell Mask Membrane stain fluorescence was excited with laser light of λ = 639 nm and emission was detected at 685–800 nm. Obtained cell images were quantitatively analyzed for changes in mitochondrial membrane potential by correlation of red and green fluorescence signals in each cell. Analysis and calculation of several correlation factors (RWC [37]) was obtained with cell image analysis software (CellProfiler, Stable 3.1.8)

For imaging flow cytometry analysis, 6.5 × 10^5^ MDA-MB-231 or MCF-7 cells were treated with RL2 or CCCP (positive control) for indicated time points. After harvesting the cells by trypsination, the cells were stained with 20 µM TMRM (MitoProbe™ TMRM Kit for Flow Cytometry, M20036, Invitrogen). The samples were loaded to the FlowSight^®^ Imaging Flow Cytometer (Amnis/Merck Millipore, Darmstadt, Germany) and excited with a 581 nm laser. Emission was detected in channel 3 and bright field images were acquired in channel 1. For every sample, 10,000 events were recorded. Data were analyzed with IDEAS software version 6.2 (Amnis/Merck Millipore, Darmstadt, Germany). RL2-induced loss of mitochondrial membrane potential was calculated via the percentage of TMRM negative cells.

### 4.12. Statistics

The shown statistical analysis was performed with GraphPad Prism (Version 8.3.0 [38]). The paired Student *t*-test and one-way ANOVA were performed as indicated. The shown *p*-values indicate the calculated significance: ns (not significant; *p* > 0.05), * (significant; *p* < 0.05), ** (significant; *p* < 0.01), *** (significant; *p* < 0.005), **** (significant; *p* < 0.001).

## 5. Conclusions

Contemporary anticancer research strongly requires the establishment of antitumor therapies that are specific for a particular cancer type. Previous studies have shown that Lactaptin and especially its recombinant analogue RL2 have strong antitumor effects. In this study, we have identified the cell death network of RL2. In particular, we have identified the mitochondrial protein TOM70 crucial for RL2-induced cell death. Thus, TOM70 is a promising target for combinatorial therapeutic strategies based on RL2-derived peptides obtained in future clinical development.

## Supplementary Materials

The following are available online at https://www.mdpi.com/2072-6694/12/6/1427/s1, Figure S1: Relative Western Blot quantifications of Figure 1B,C, Figure S2: Relative Western Blot quantifications of Figure 3, Figure S3: Relative Western Blot quantifications of Figure 4A,B,C, Figure S4: Relative Western Blot quantifications of Figure 5, Figure S5: TMRM-indicated mitochondrial membrane potential loss of MDA-MB-231 cells, Figure S6: Relative Western Blot quantifications of Figure 7, Table S1: Absolute values of unique peptides and coverages of potential RL2-interacting proteins identified by mass spectrometry. The analysis of potential RL2-interacting proteins in Figure 4B, are based on the absolute values shown here. The abbreviations n1, n2 and n3 indicate absolute values for every experimental replicate, Table S2: Absolute values of unique peptides and coverages of all mitochondrial proteins identified by mass spectrometry. Abbreviations n1, n2 and n3 indicate absolute values for every experimental replicate.

## Abbreviations

ACN	Acetonitrile
ACTN1	Actin
AIF	apoptosis inducing factor
CCCP	Carbonylcyanid-m-chlorphenylhydrazon
Cyt c	Cytochrome c
DISC	death inducing signaling complex
DR	death receptor
EndoG	Endonuclease G
FA	Formic acid
IP	Immunoprecipitation
MOMP	mitochondrial outer membrane permeabilization
OMM	outer mitochondrial membrane
RL2	recombinant Lactaptin 2
RU	relative units
TFA	Trifluoroacetic acid
TIM/TOM	translocase of the inner/outer membrane
TMRM	Tetramethylrhodamin-methylester
TBB5	Tubulin
