# Supplementary material for: The Recombinant Fragment of Human κ-Casein Induces Cell Death by Targeting the Proteins of Mitochondrial Import in Breast Cancer Cells
Source: Cancers (Basel). 2020 May 31;12(6):1427. doi: 10.3390/cancers12061427 (PMC7352597; doi:10.3390/cancers12061427)

Figure 1B – RL2 blot

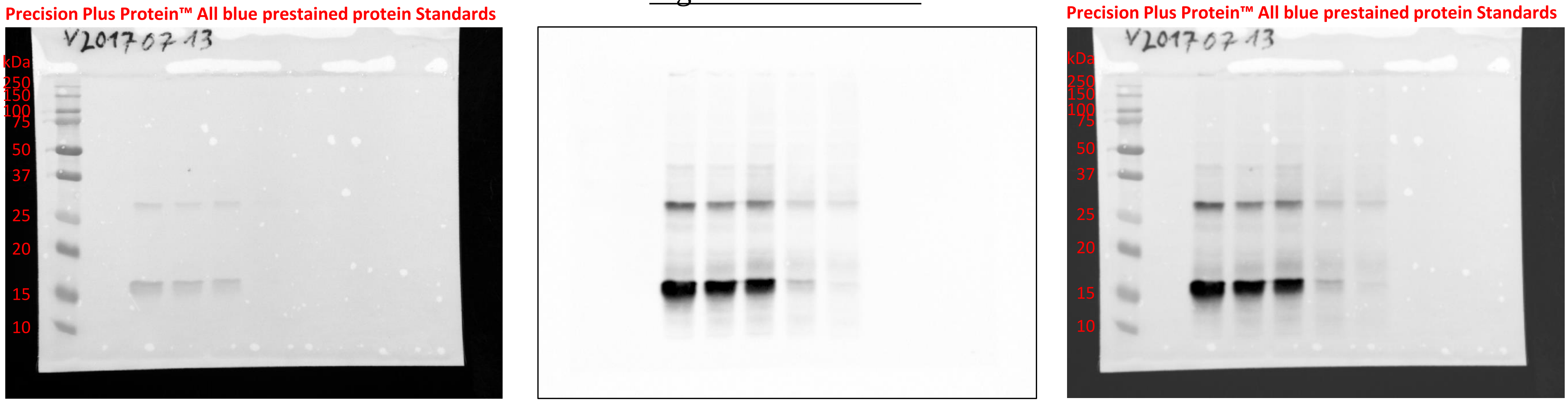

Figure 1B – Actin blot

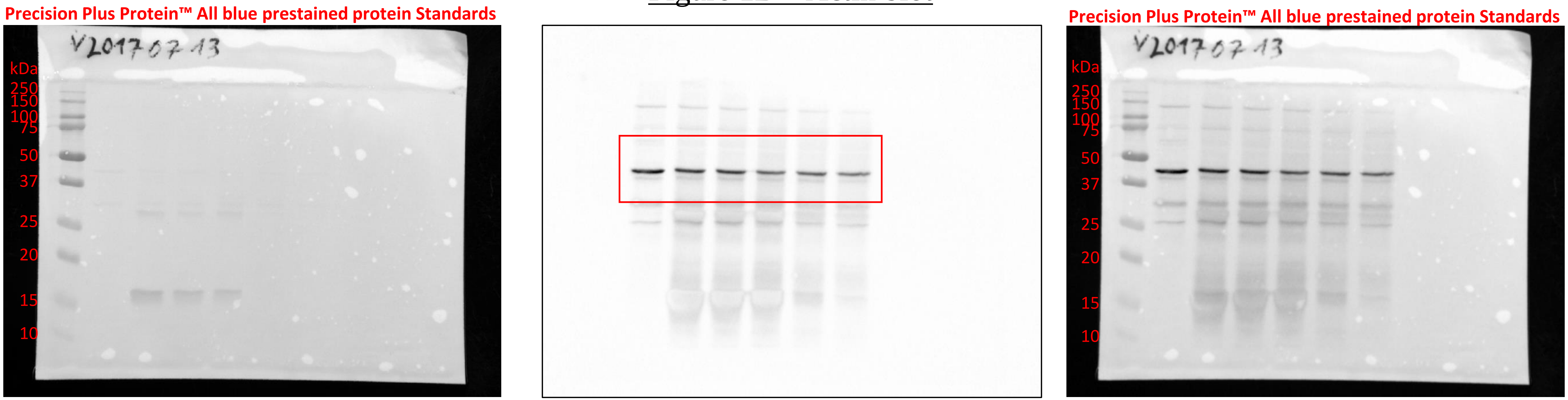

Figure 1C – RL2 blot

Precision Plus Protein™ All blue prestained protein Standards

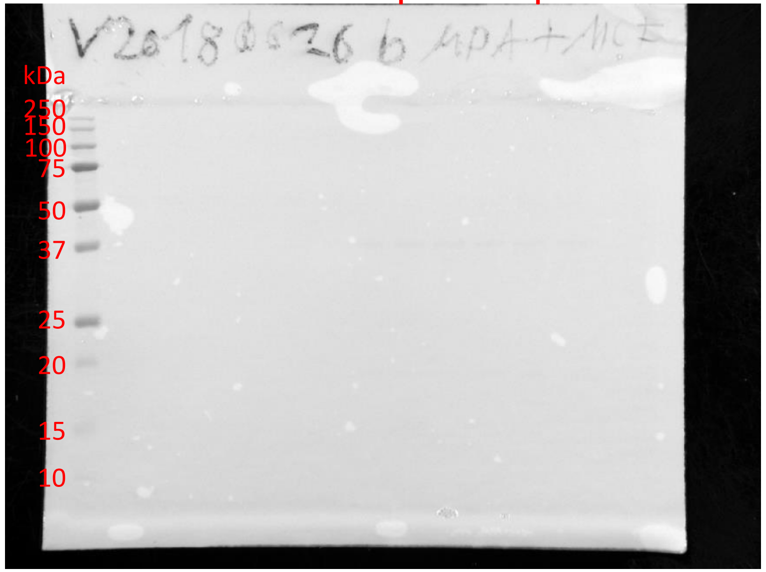

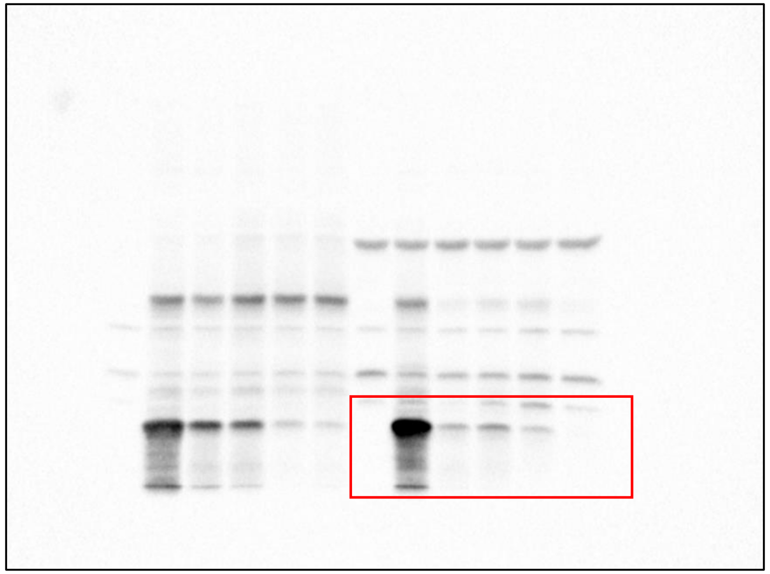

Precision Plus Protein™ All blue prestained protein Standards

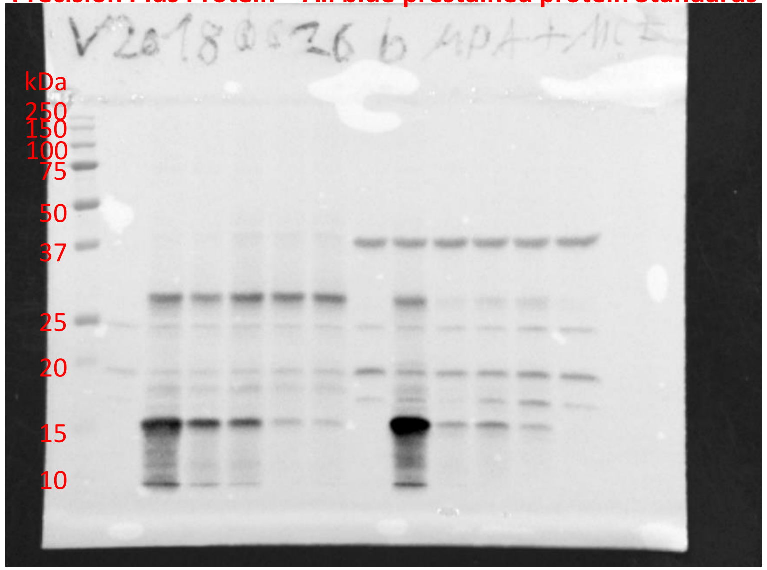

Figure 1C – Actin blot

Precision Plus Protein™ All blue prestained protein Standards

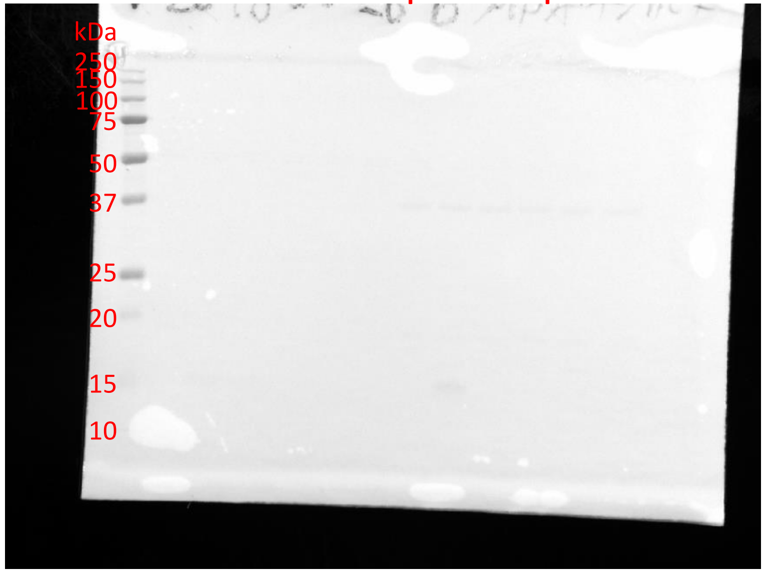

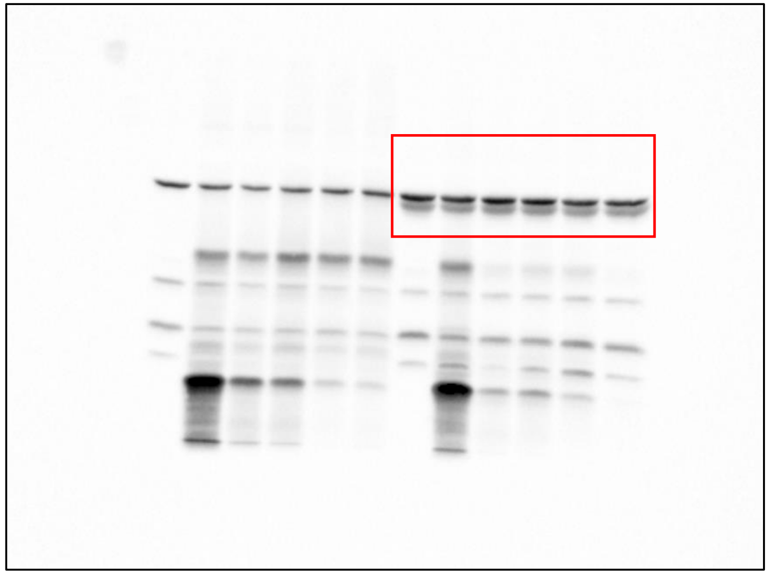

Precision Plus Protein™ All blue prestained protein Standards

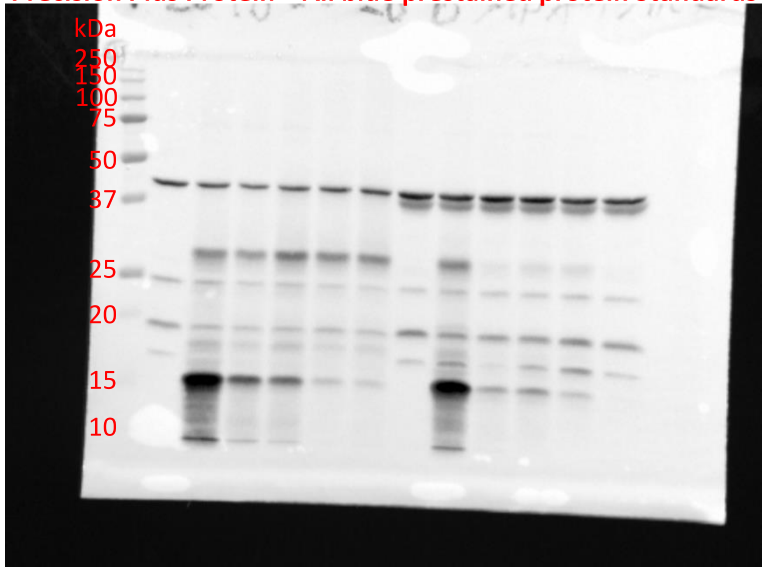

Figure 3C – Procaspase-3 short exposure blot

Precision Plus Protein™ All blue prestained protein Standards

Precision Plus Protein™ All blue prestained protein Standards

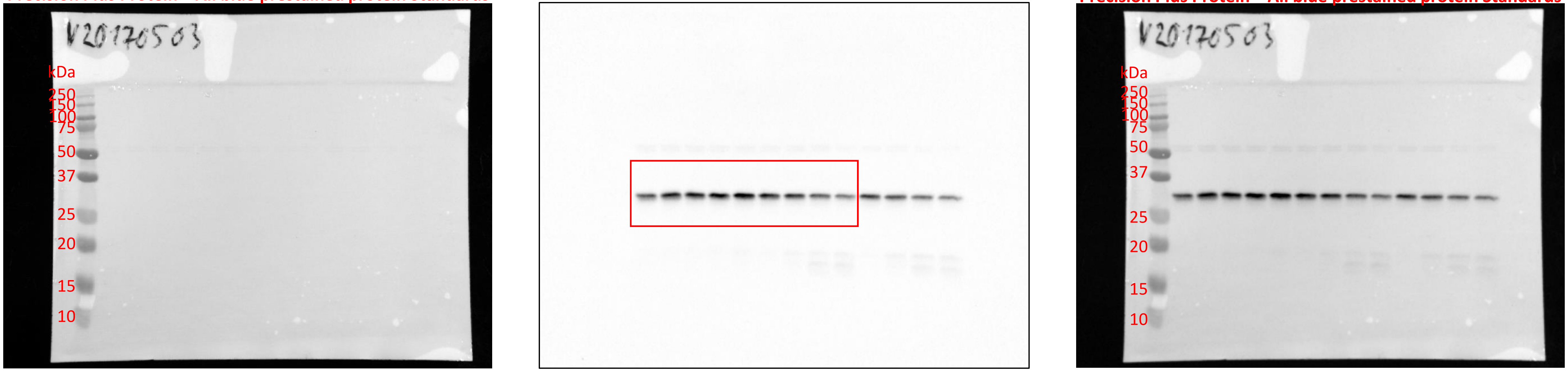

Figure 3C – Procaspase-3 long exposure blot

Precision Plus Protein™ All blue prestained protein Standards

Precision Plus Protein™ All blue prestained protein Standards

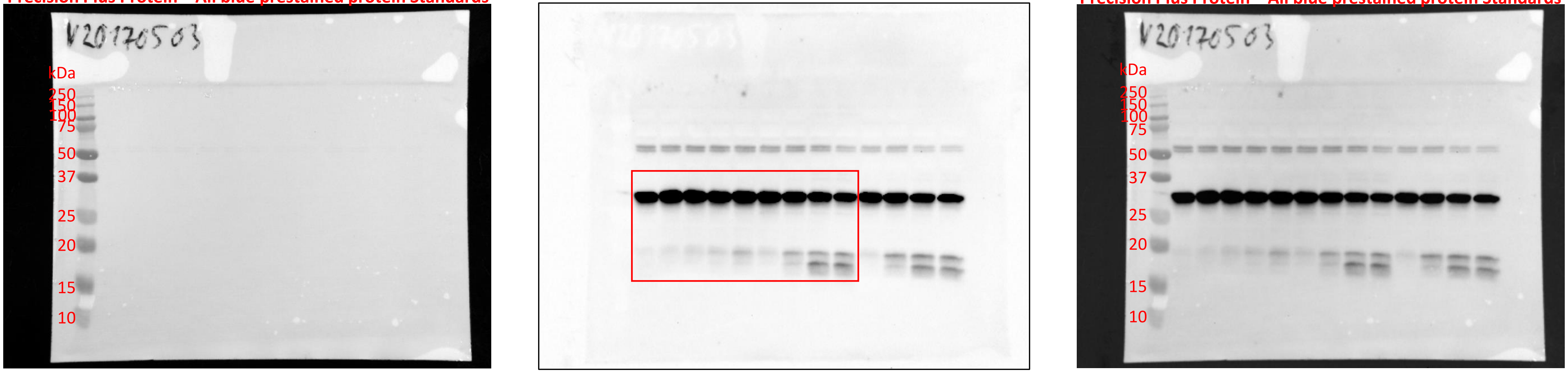

Figure 3C – BID blot

Precision Plus Protein™ All blue prestained protein Standards

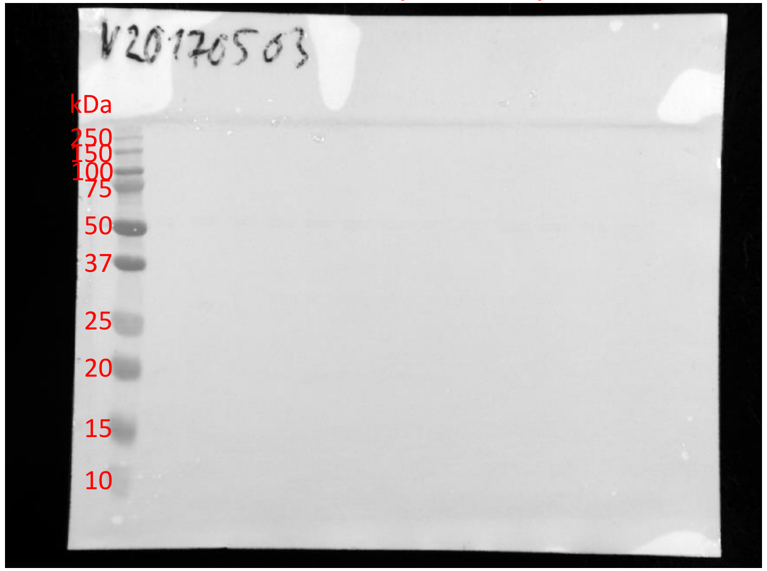

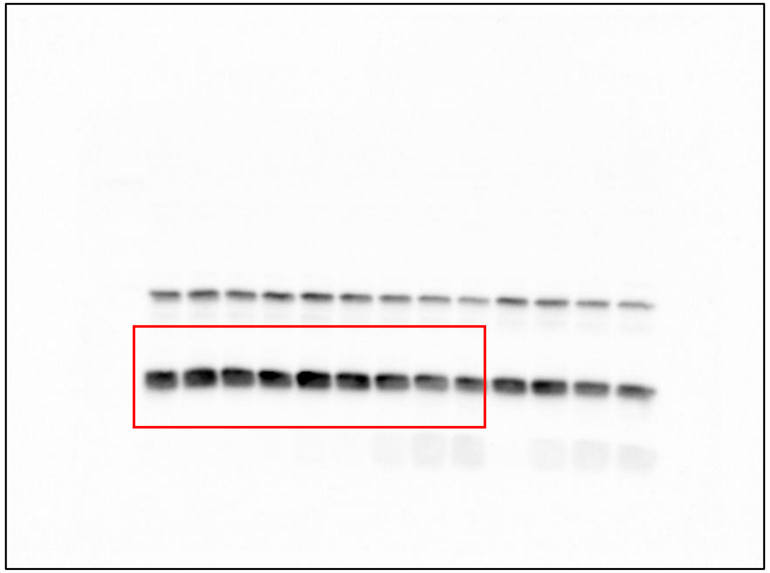

Precision Plus Protein™ All blue prestained protein Standards

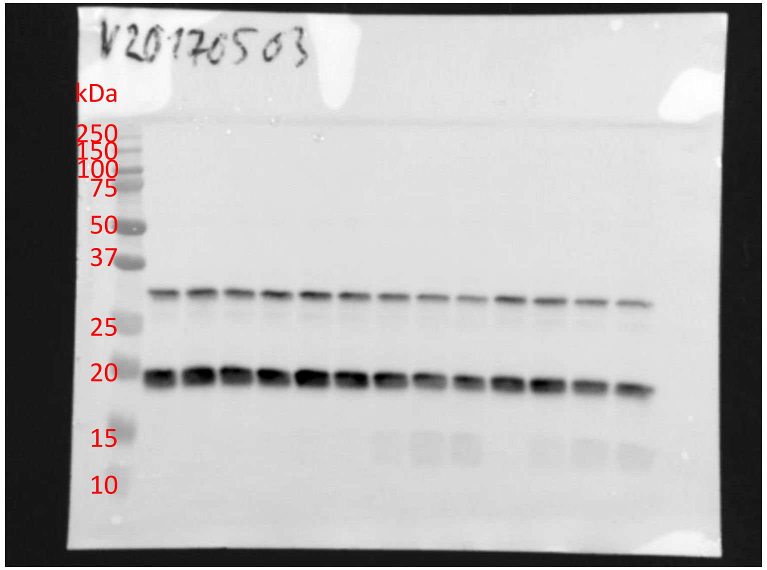

Figure 3C – tBID blot

Precision Plus Protein™ All blue prestained protein Standards

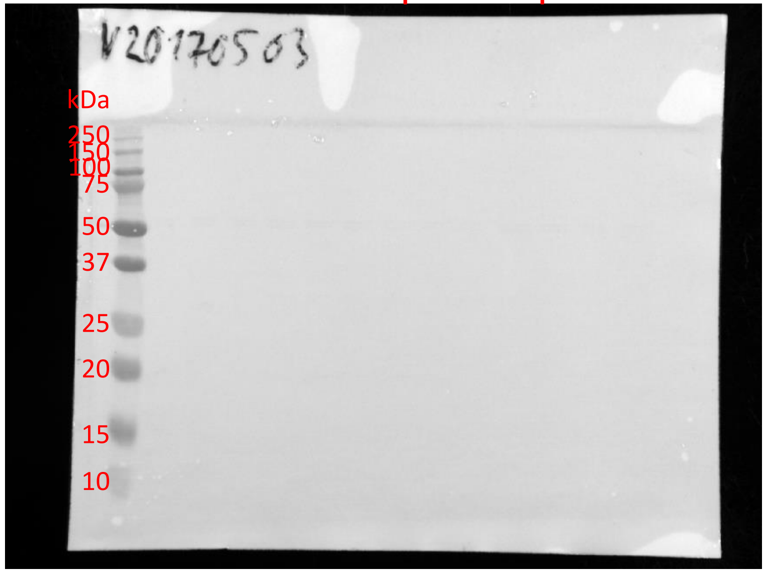

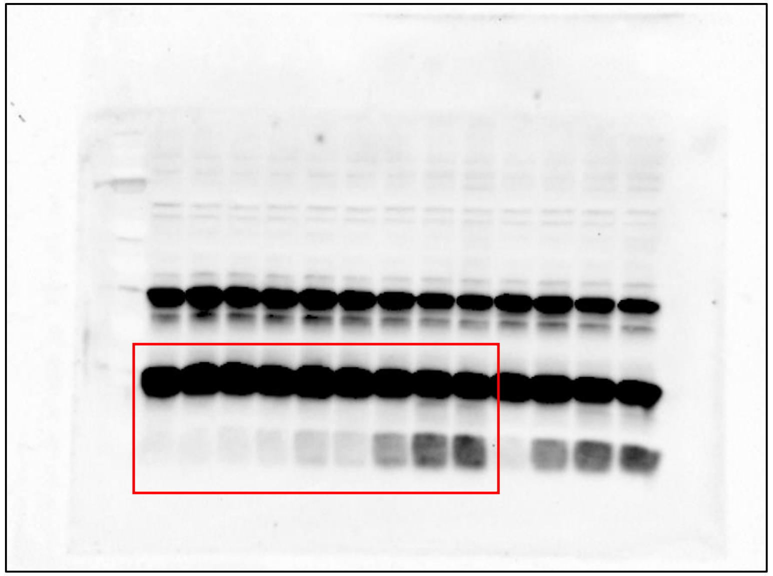

Precision Plus Protein™ All blue prestained protein Standards

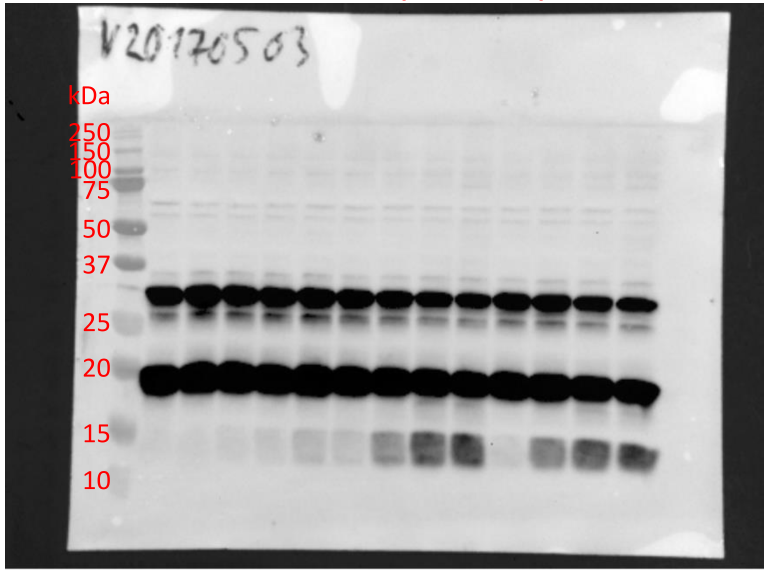

Figure 3C – Procaspase-8 short exposure blot

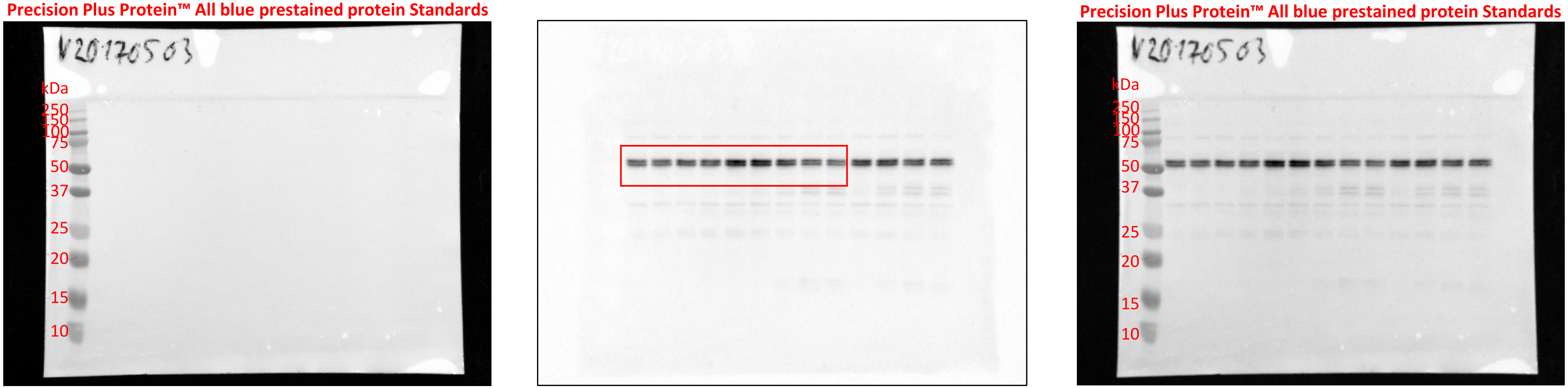

Figure 3C – Procaspase-8 long exposure blot

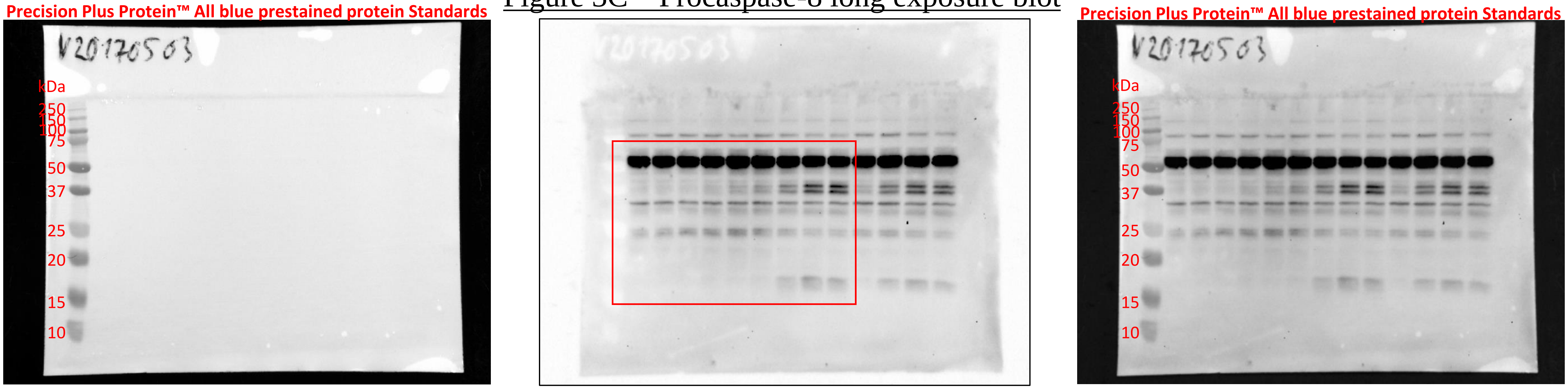

Precision Plus Protein™ All blue prestained protein Standards

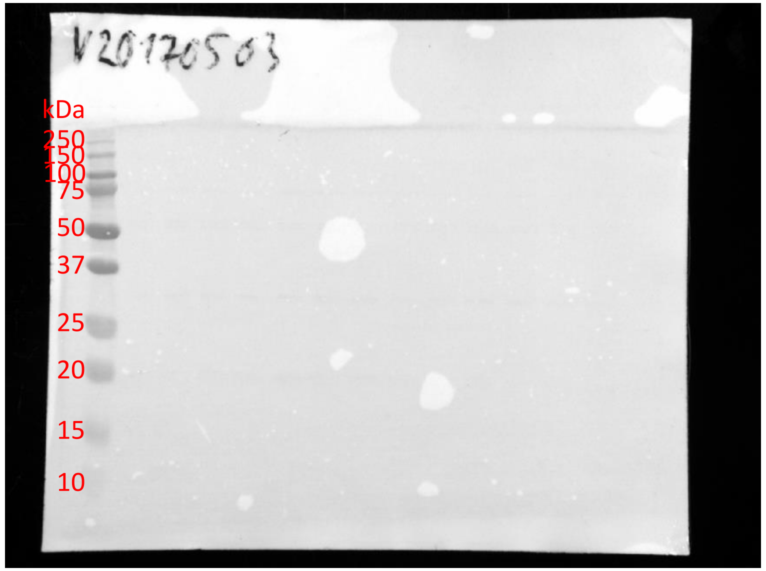

Figure 3C – PARP blot

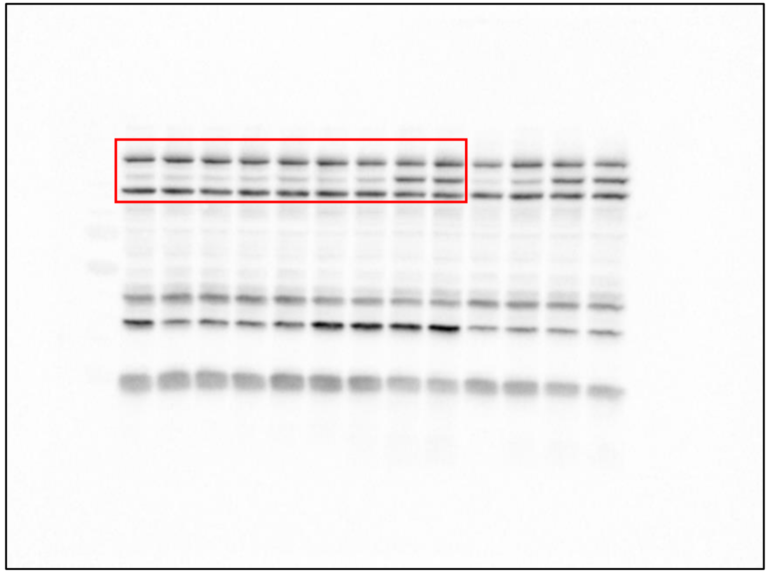

Precision Plus Protein™ All blue prestained protein Standards

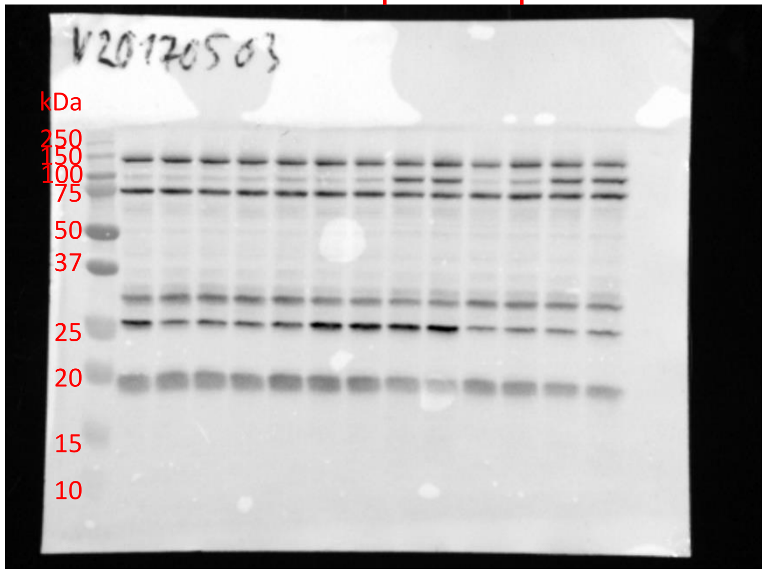

Precision Plus Protein™ All blue prestained protein Standards

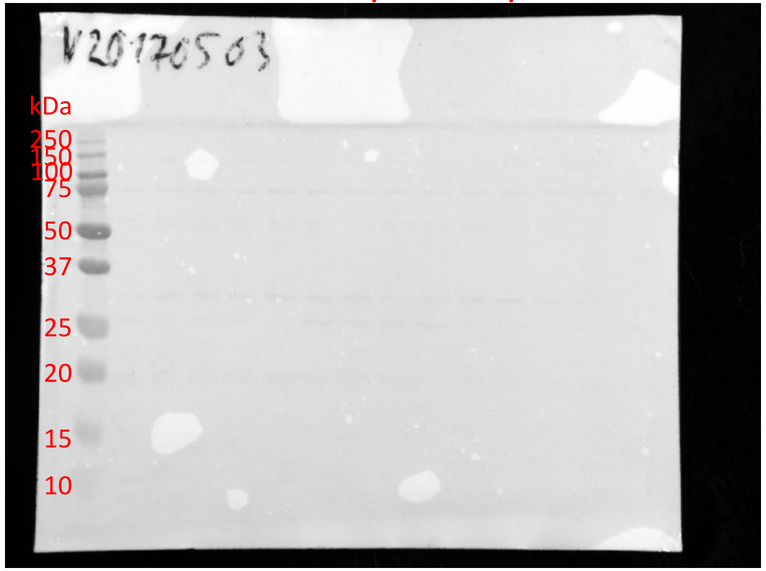

Figure 3C – Actin blot

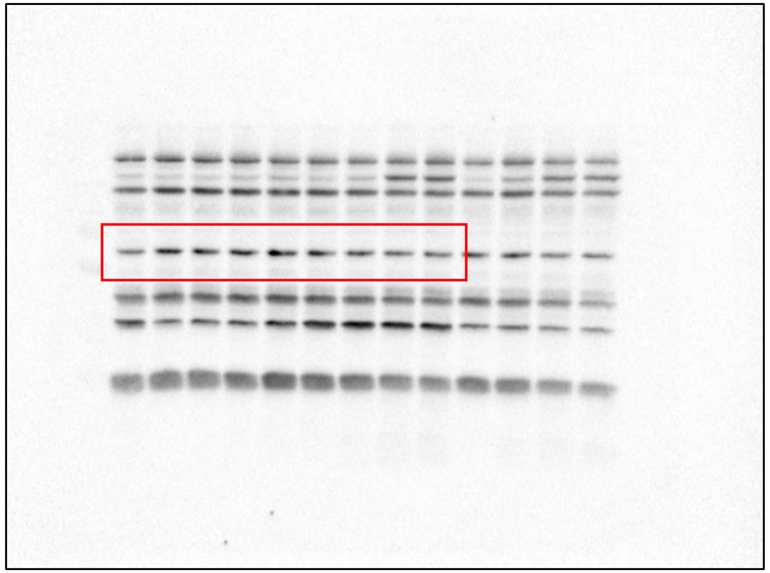

Precision Plus Protein™ All blue prestained protein Standards

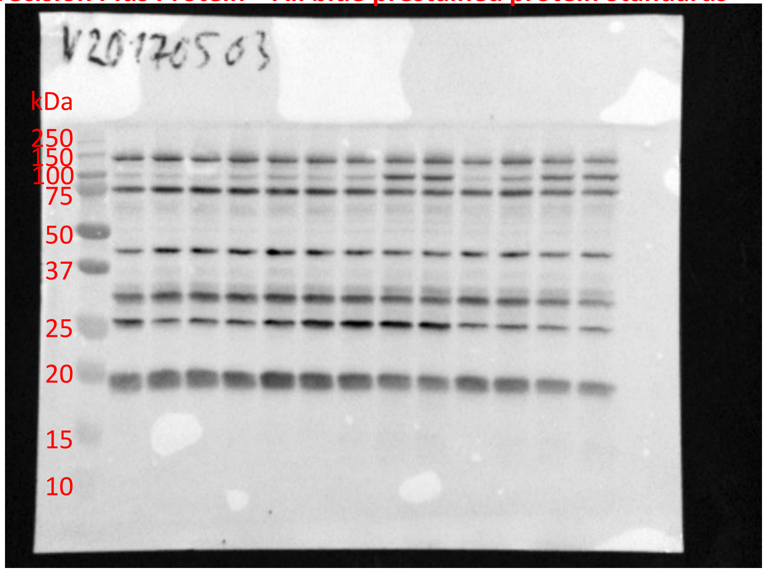

Figure 4C – Tom70 blot

Precision Plus Protein™ All blue prestained protein Standards

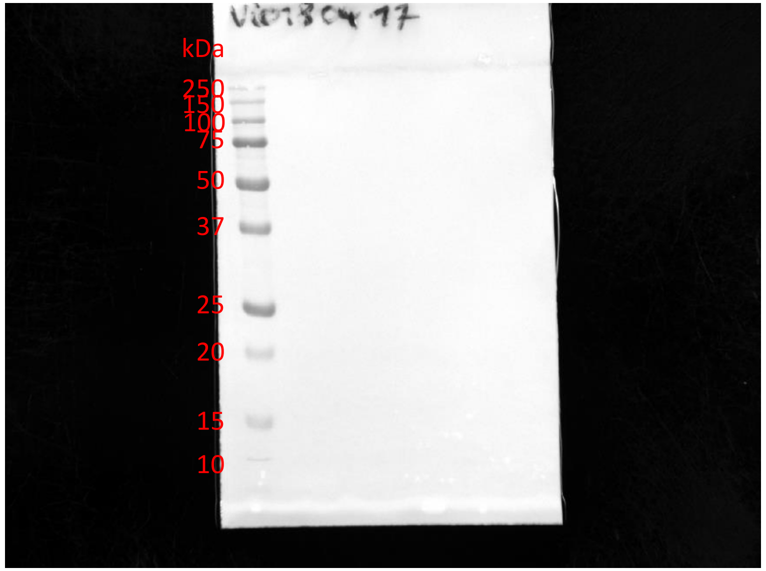

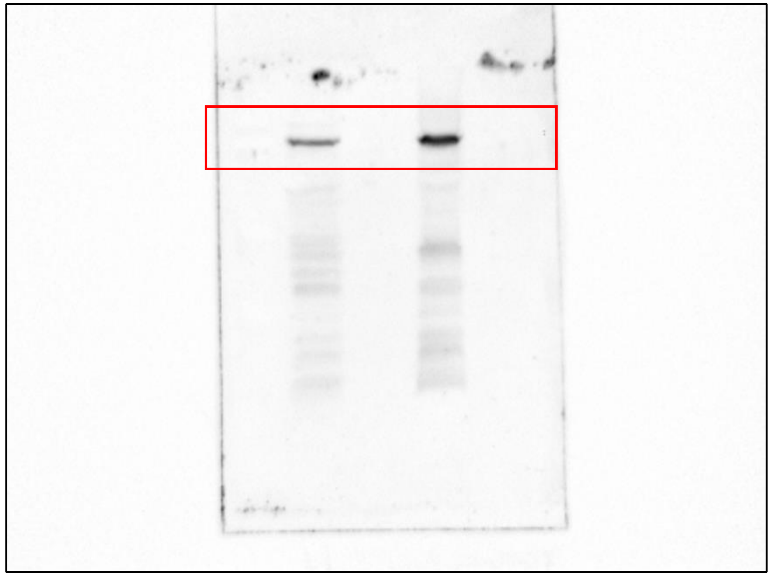

Precision Plus Protein™ All blue prestained protein Standards

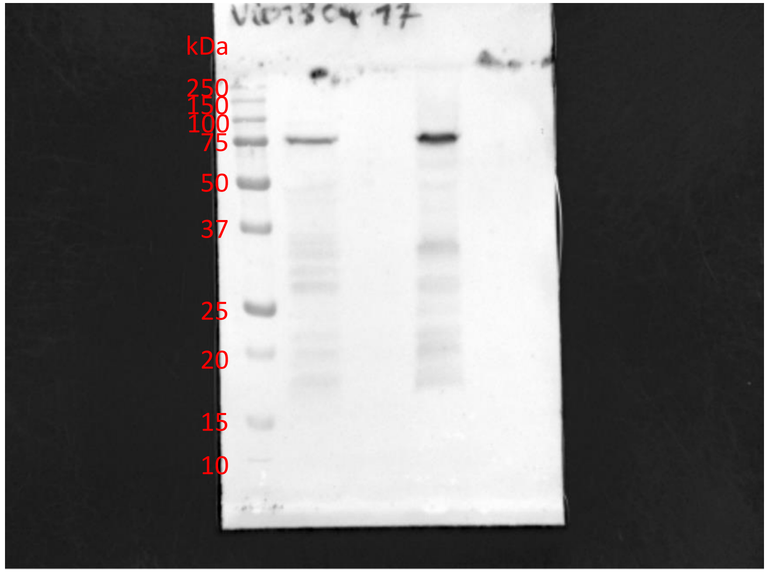

Figure 4C – RL2 blot

Precision Plus Protein™ All blue prestained protein Standards

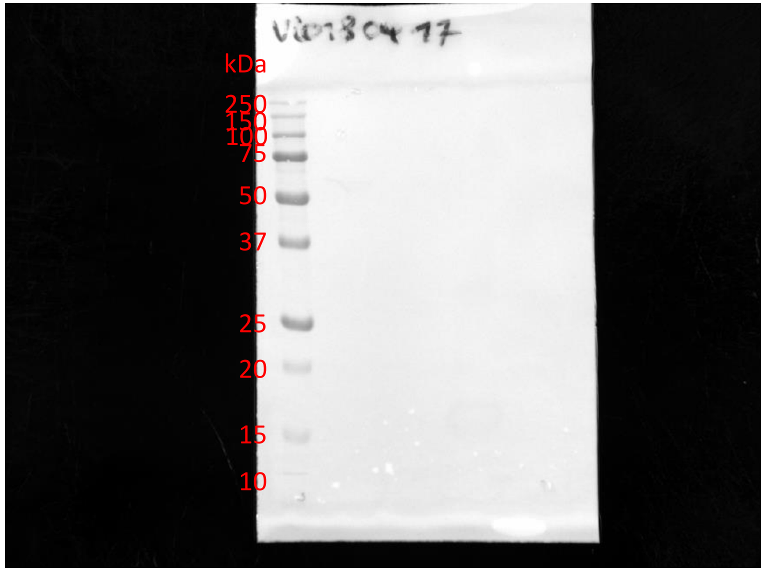

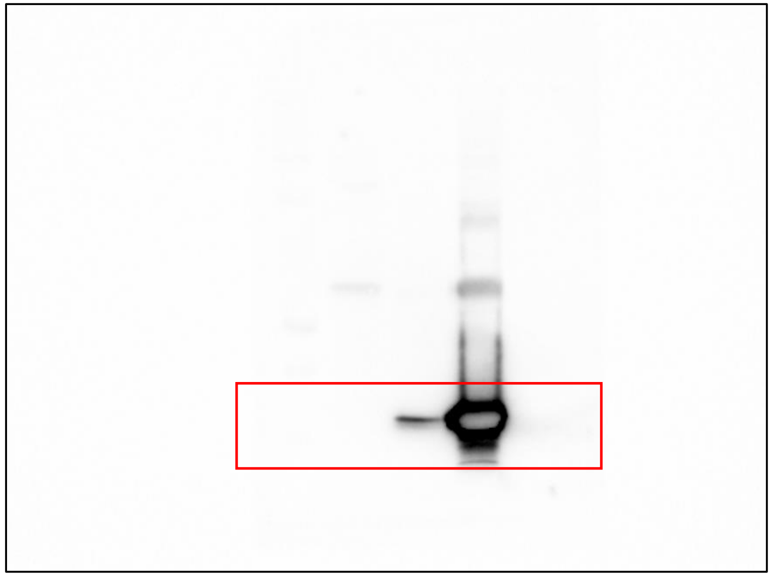

Precision Plus Protein™ All blue prestained protein Standards

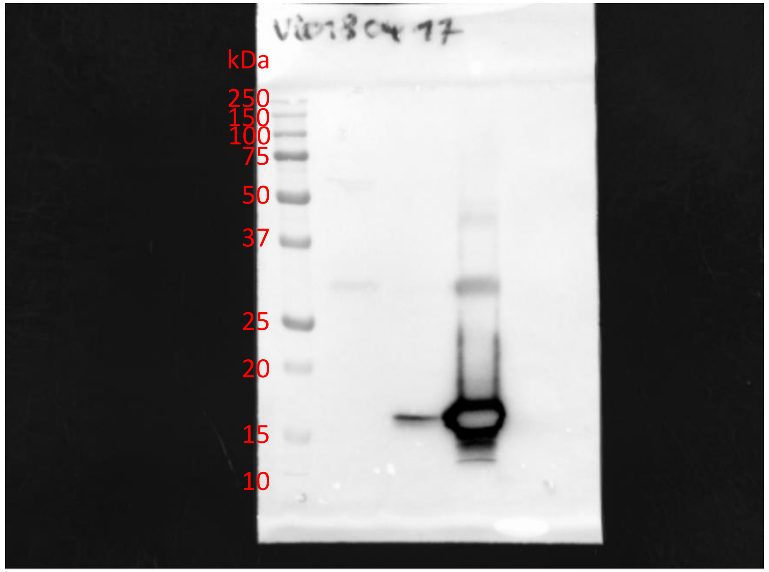

Figure 4C – BID blot

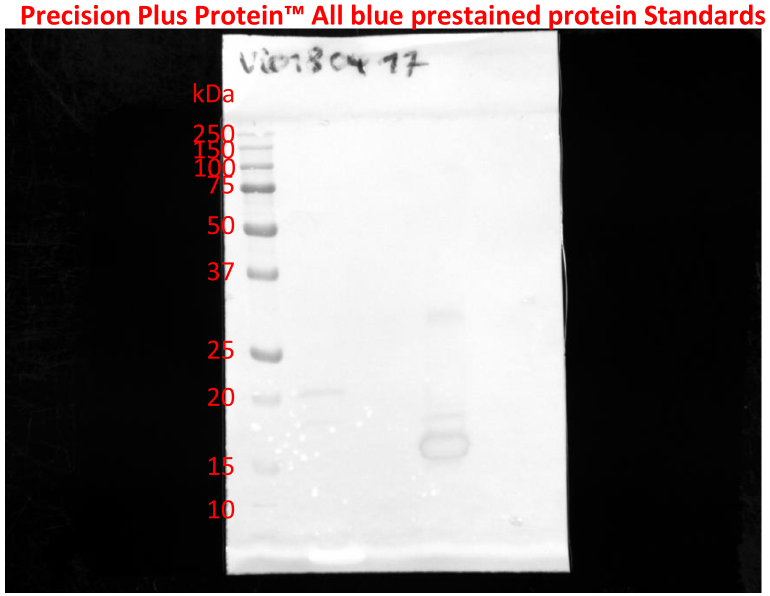

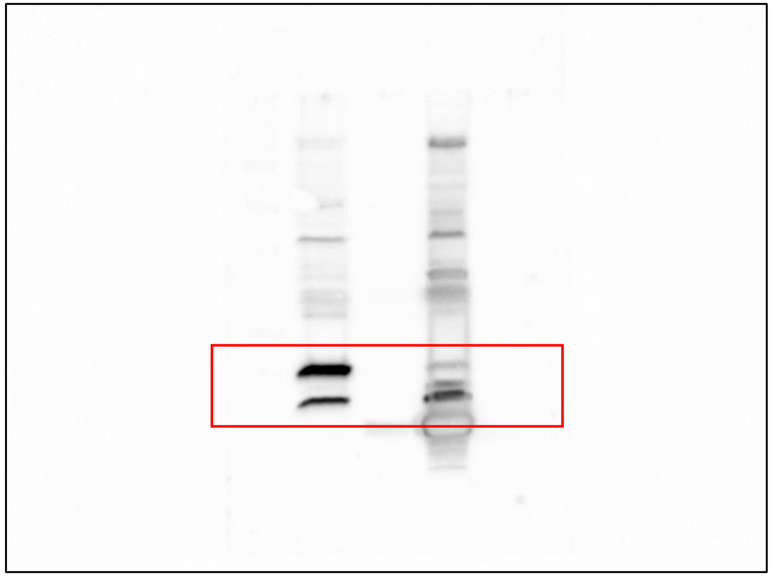

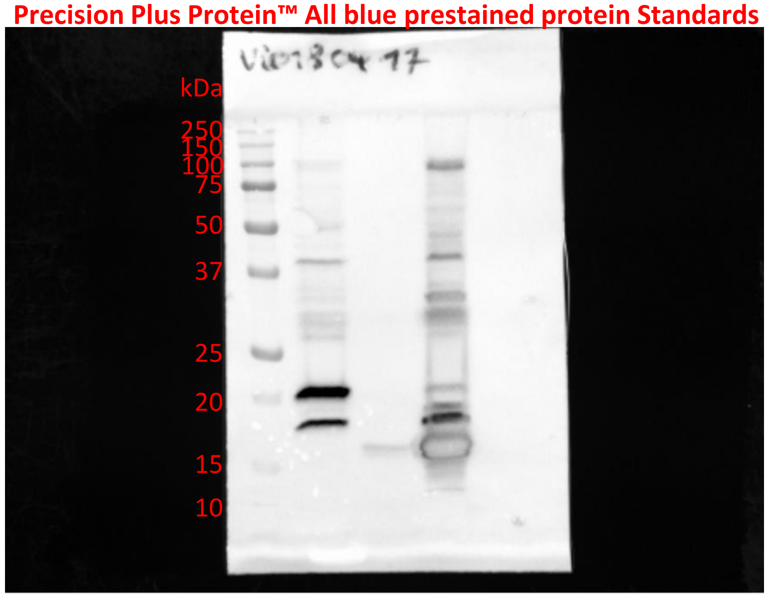

Figure 4D lysate – Tom70 blot

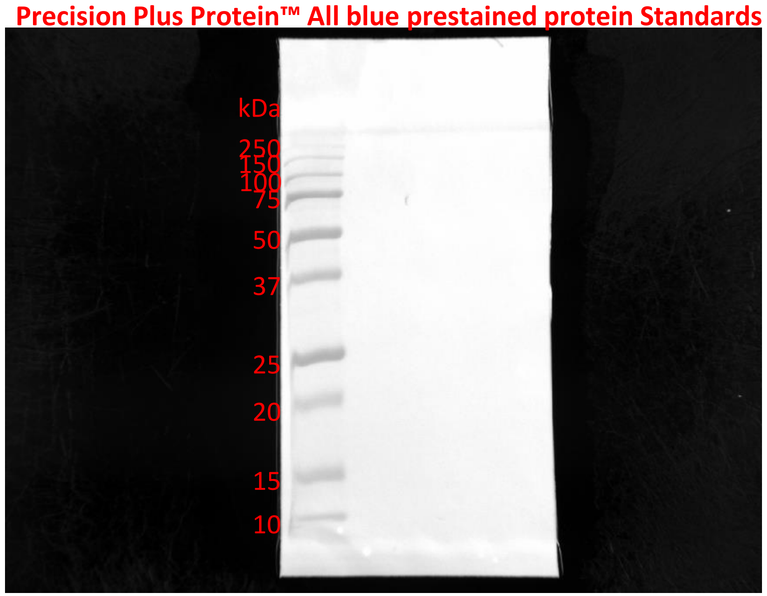

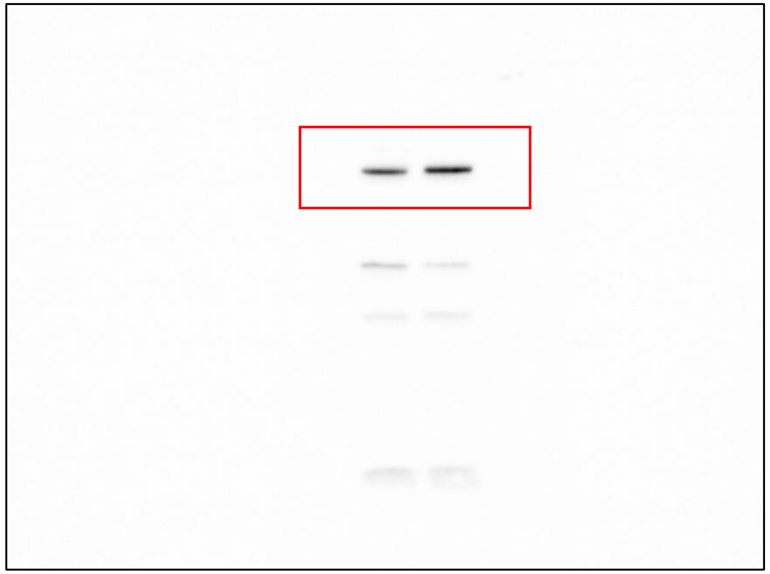

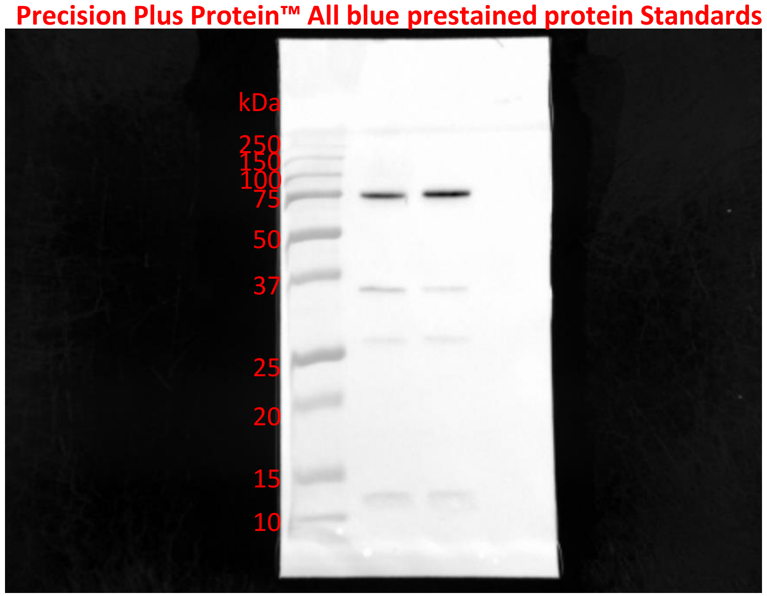

Precision Plus Protein™ All blue prestained protein Standards

Figure 4D lysate – RL2 blot

Precision Plus Protein™ All blue prestained protein Standards

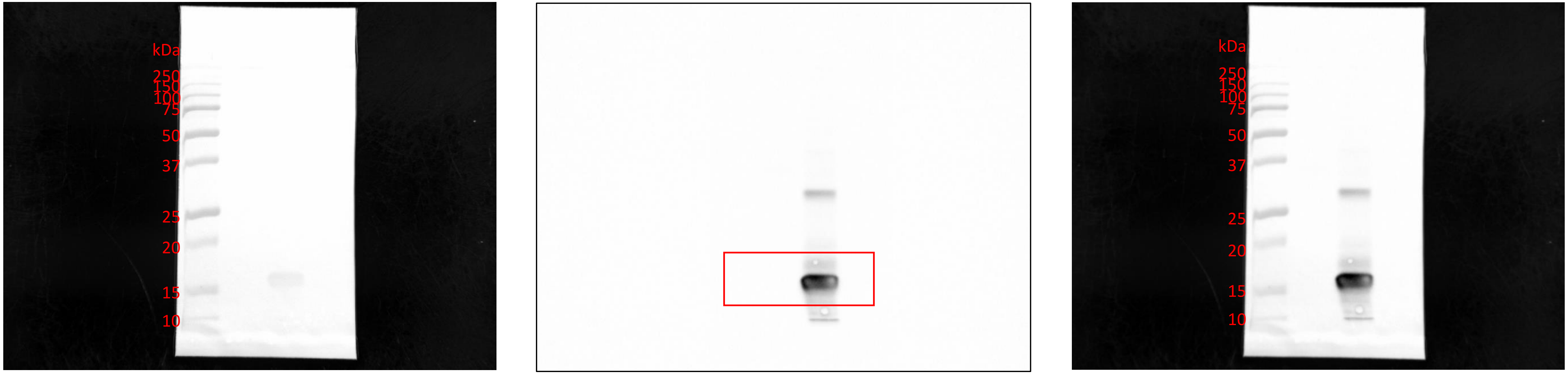

Precision Plus Protein™ All blue prestained protein Standards

Figure 4D lysate – Actin blot

Precision Plus Protein™ All blue prestained protein Standards

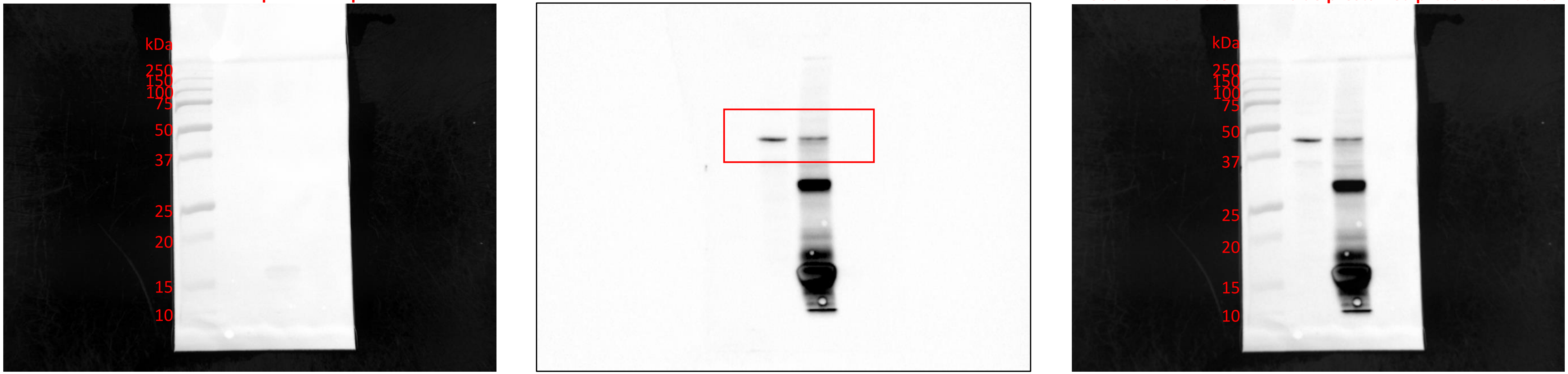

Figure 4D IP – RL2 blot

Precision Plus Protein™ All blue prestained protein Standards

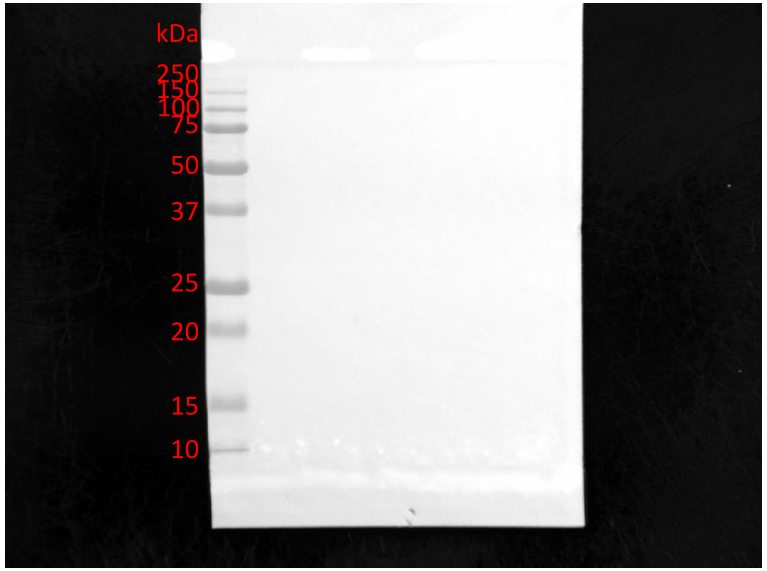

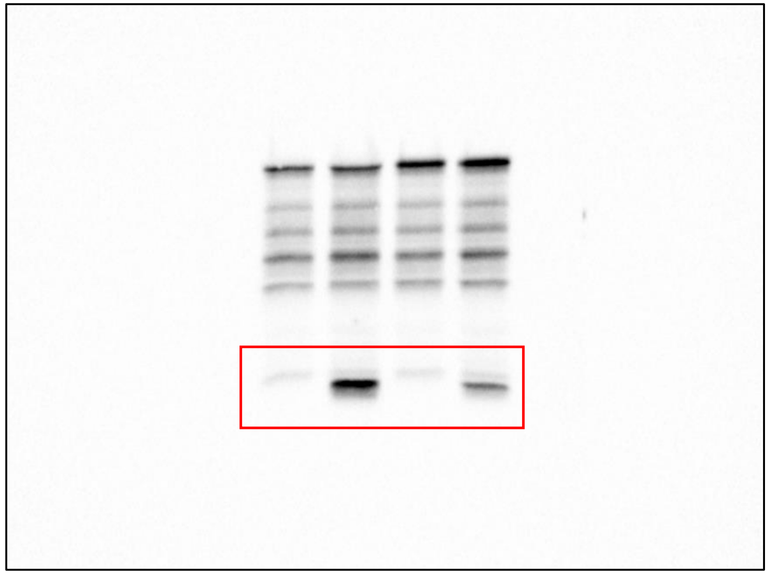

Precision Plus Protein™ All blue prestained protein Standards

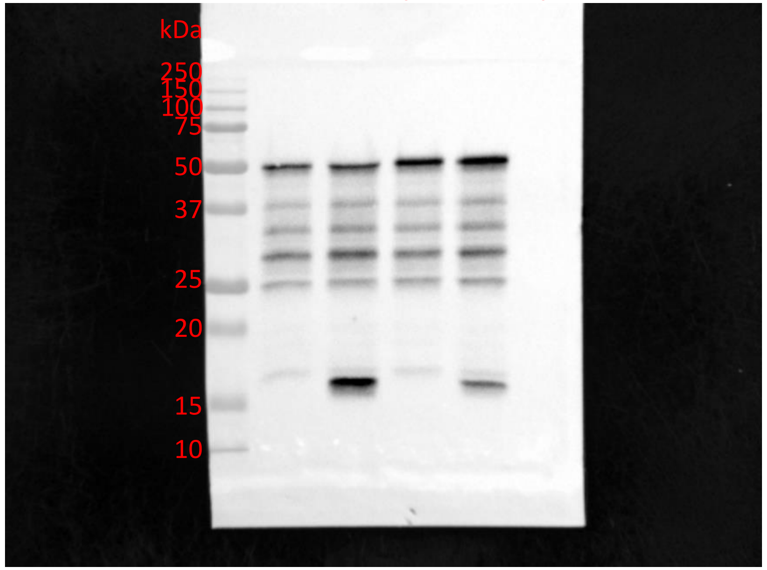

Figure 4D IP – Tom70 blot

Precision Plus Protein™ All blue prestained protein Standards

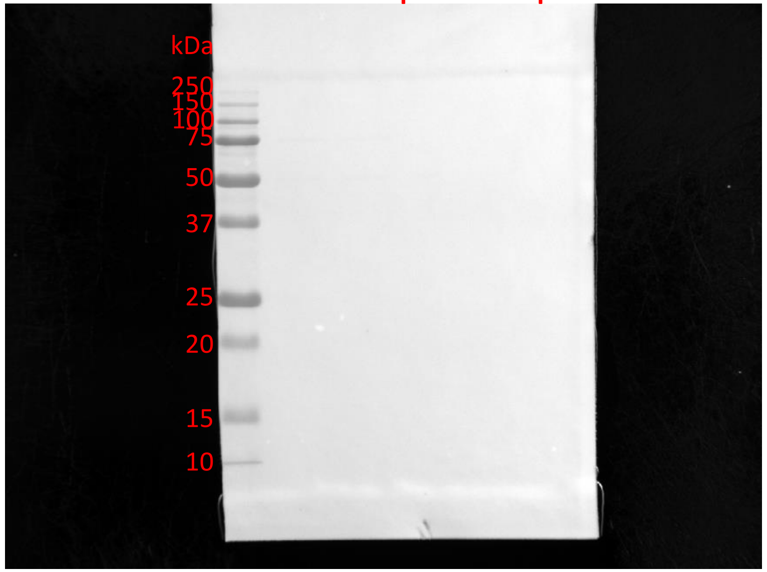

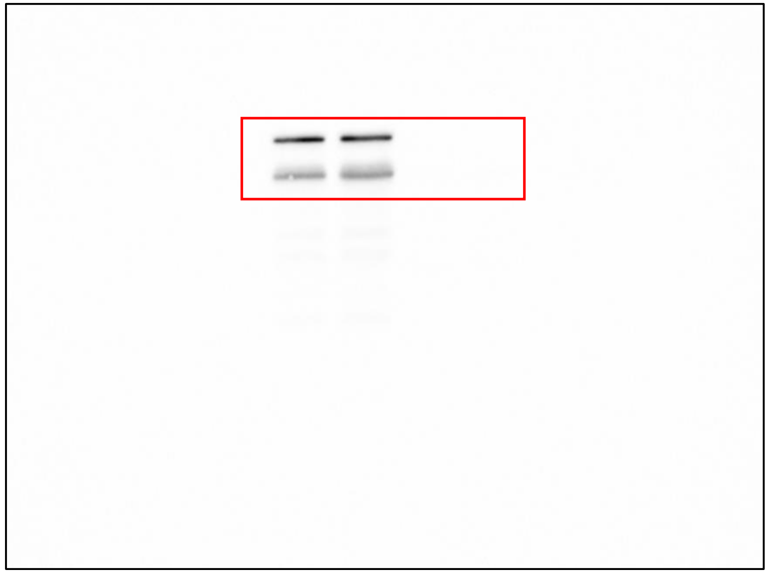

Precision Plus Protein™ All blue prestained protein Standards

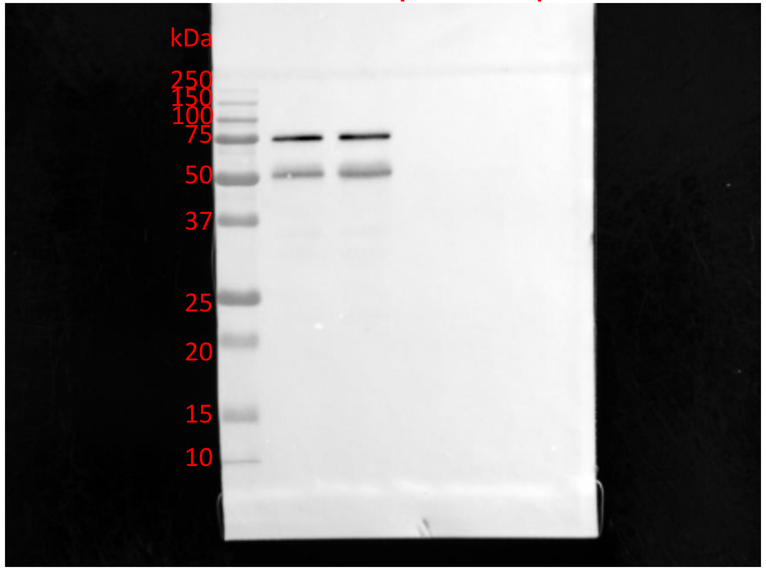

Precision Plus Protein™ All blue prestained protein Standards

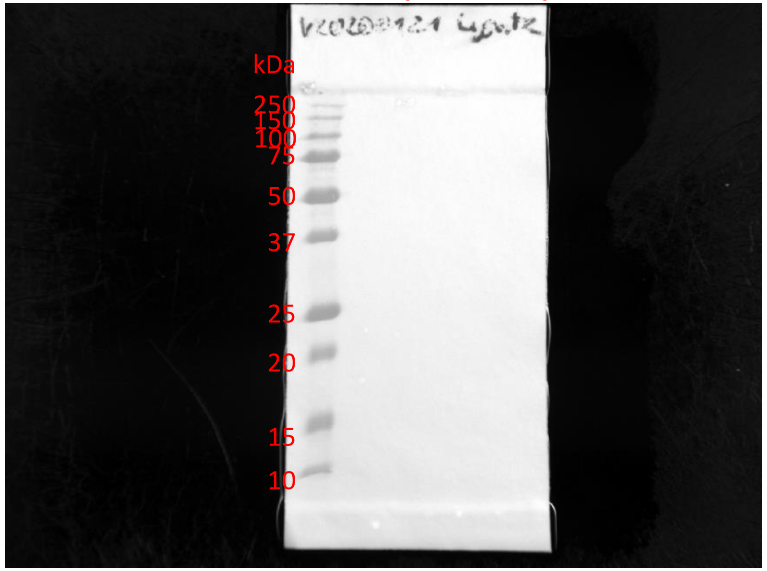

Figure 4E lysate – Tom70 blot

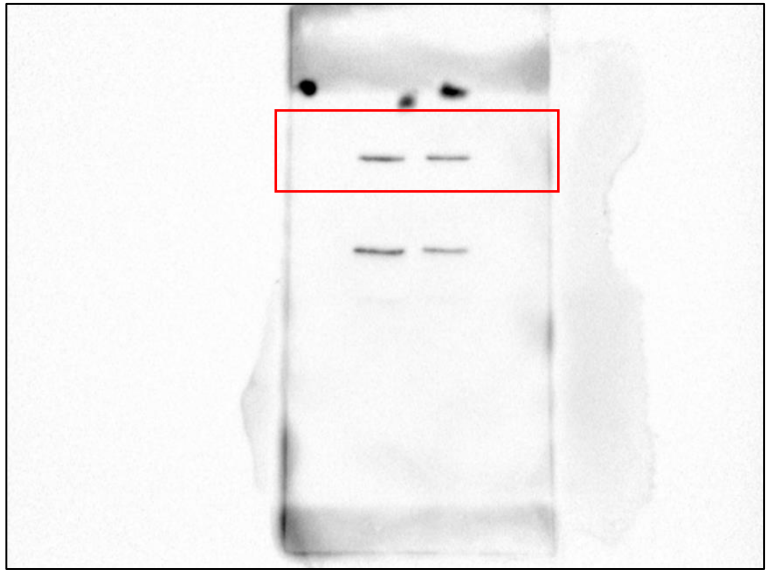

Precision Plus Protein™ All blue prestained protein Standards

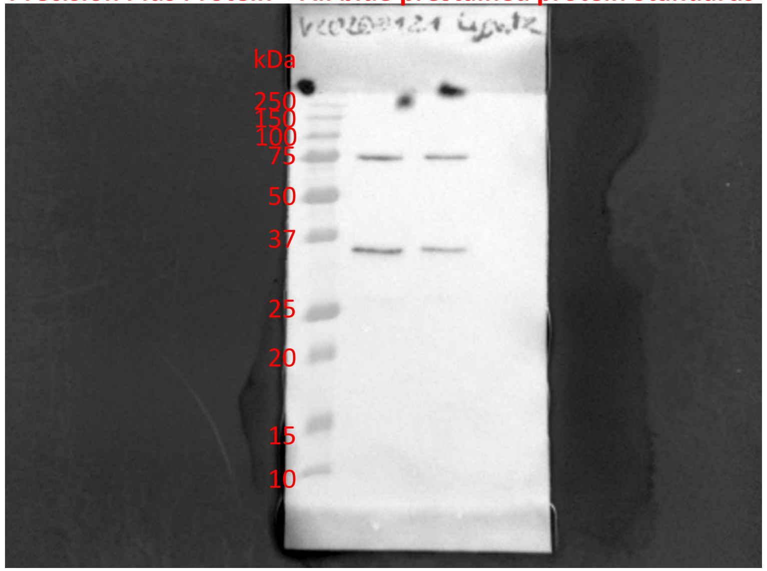

Precision Plus Protein™ All blue prestained protein Standards

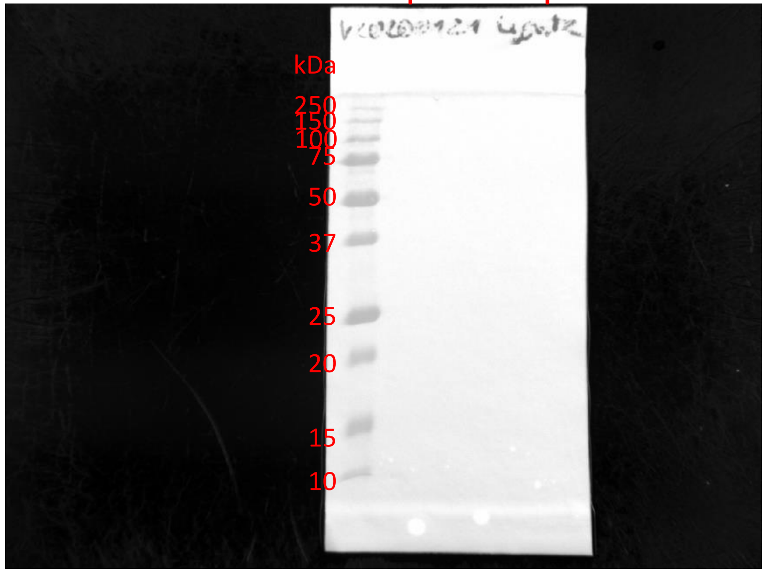

Figure 4E lysate – RL2 blot

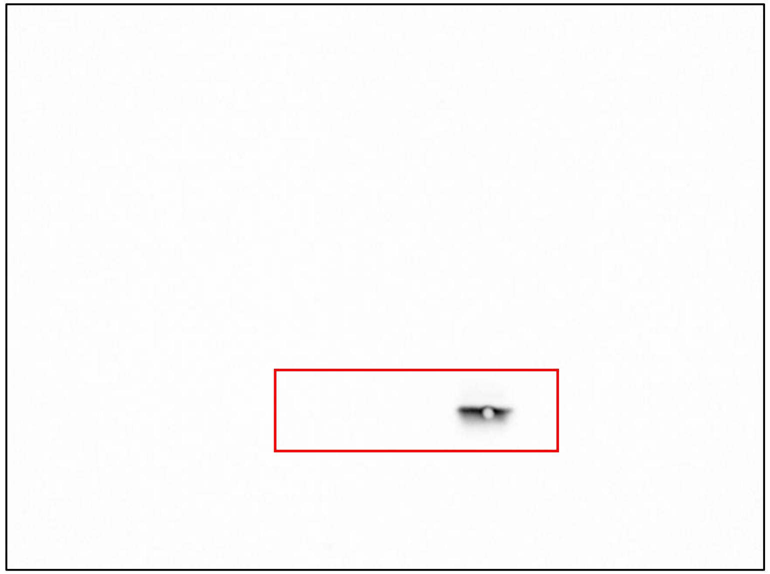

Precision Plus Protein™ All blue prestained protein Standards

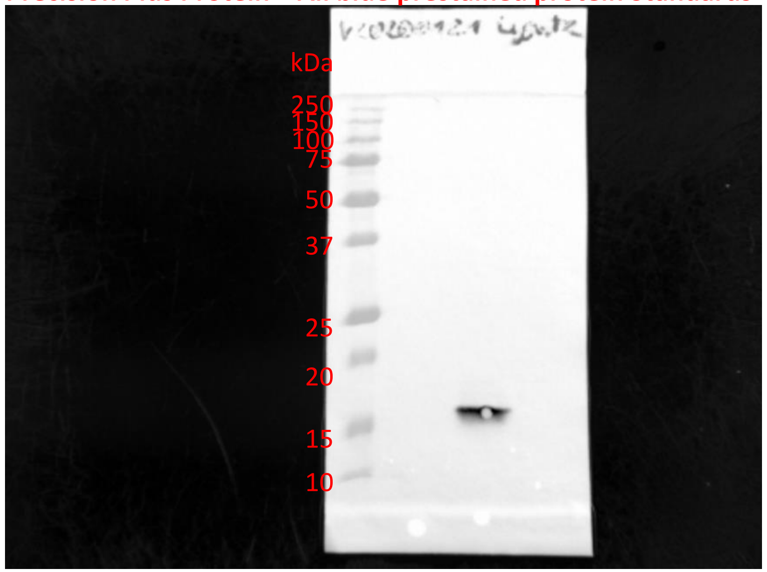

Precision Plus Protein™ All blue prestained protein Standards

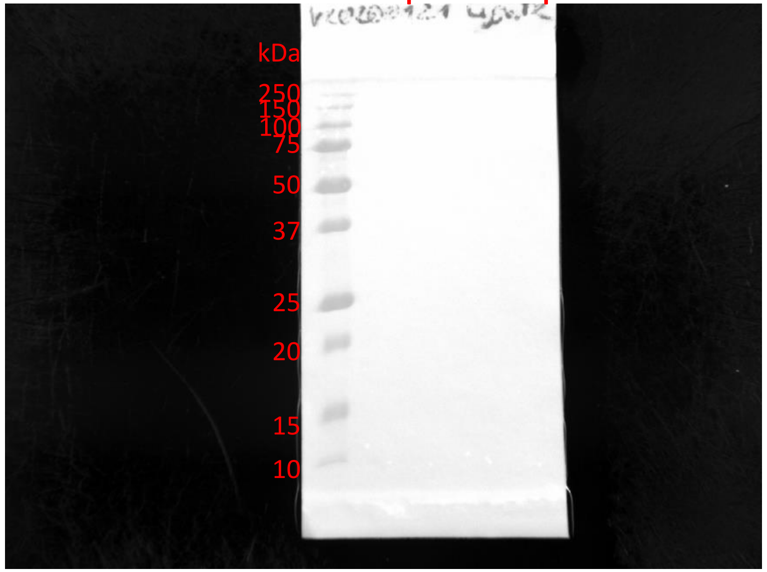

Figure 4E lysate – Actin blot

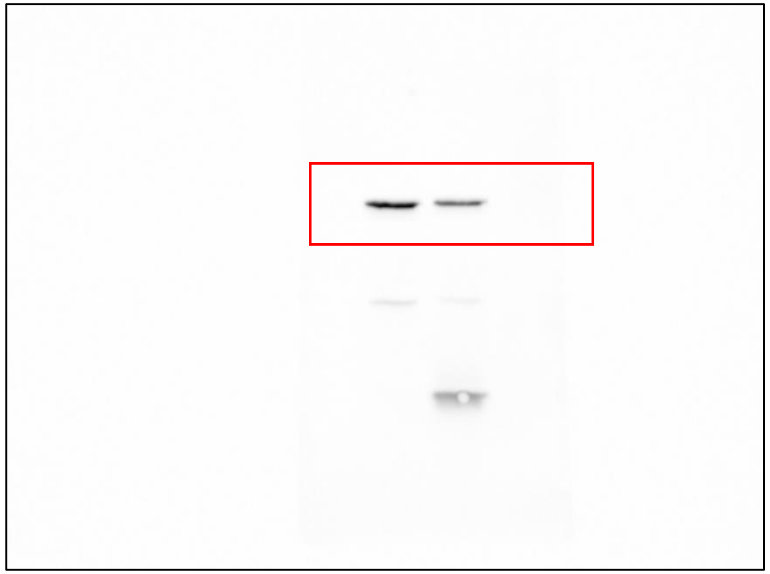

Precision Plus Protein™ All blue prestained protein Standards

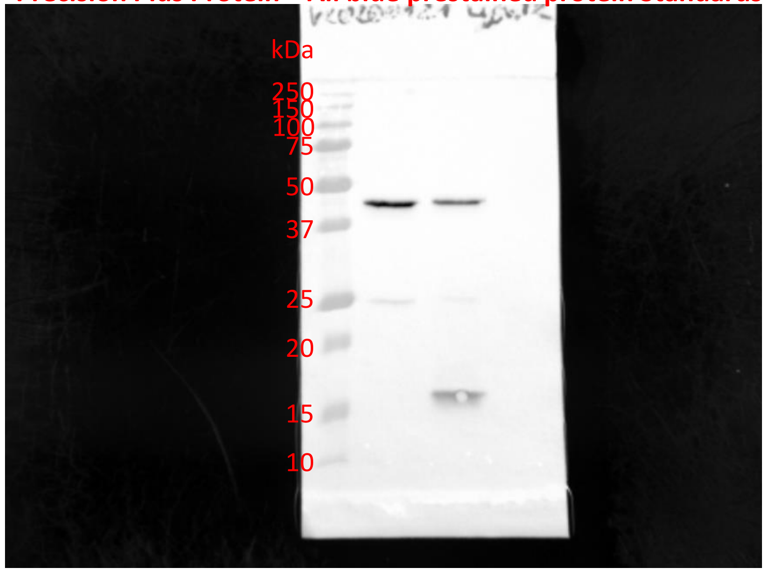

Precision Plus Protein™ All blue prestained protein Standards

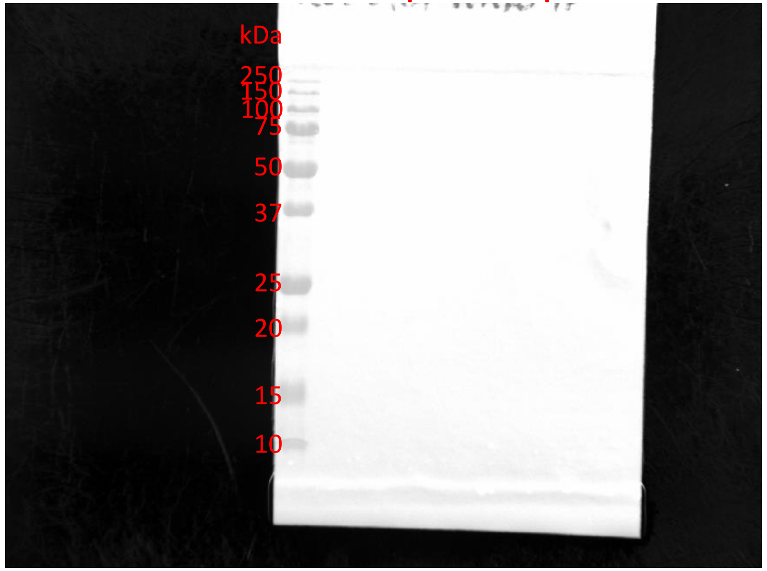

Figure 4E IP – RL2 blot

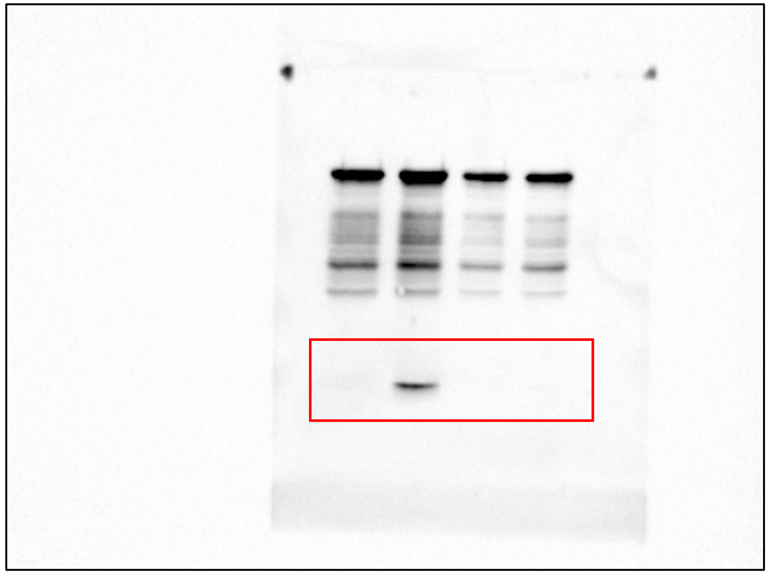

Precision Plus Protein™ All blue prestained protein Standards

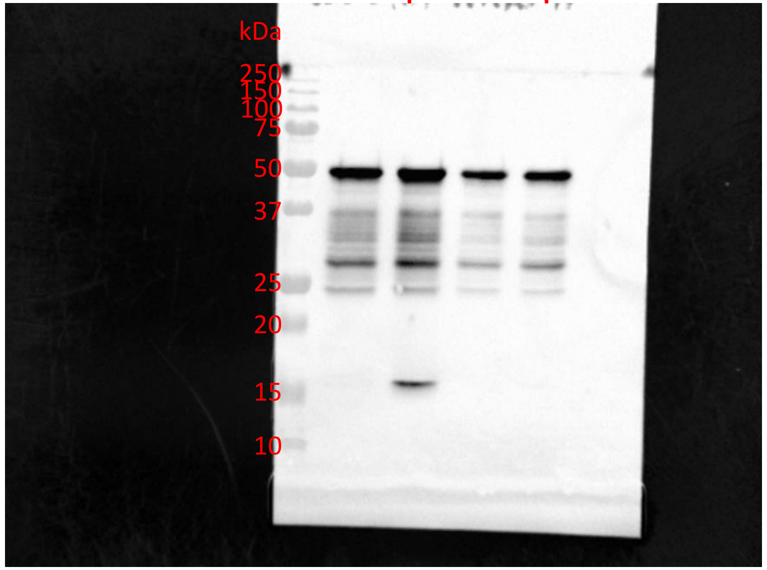

Figure 4E IP – Tom70 blot

Precision Plus Protein™ All blue prestained protein Standards

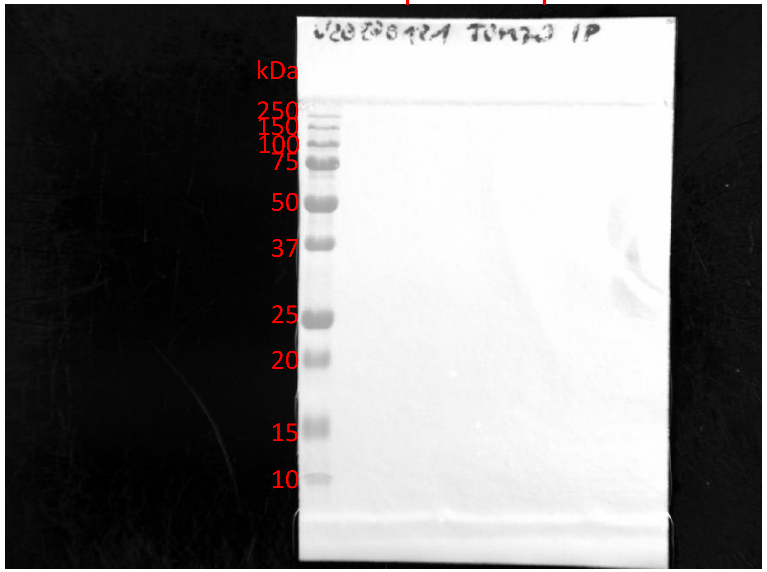

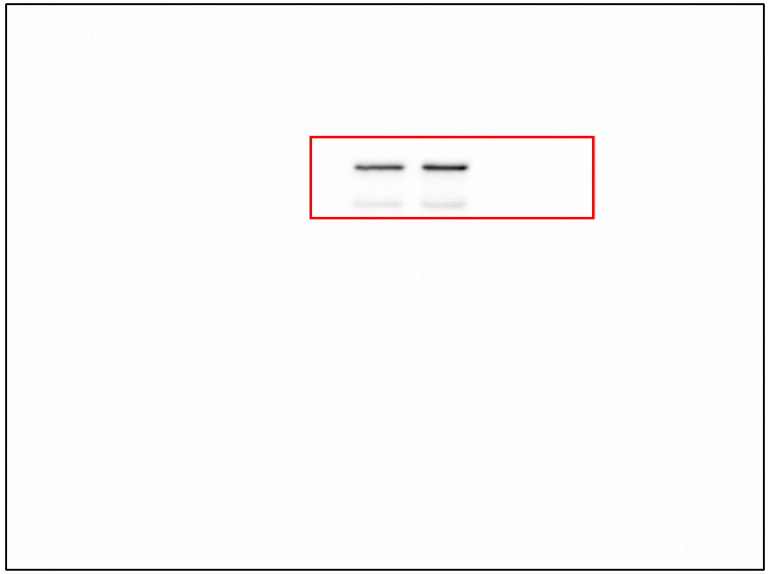

Precision Plus Protein™ All blue prestained protein Standards

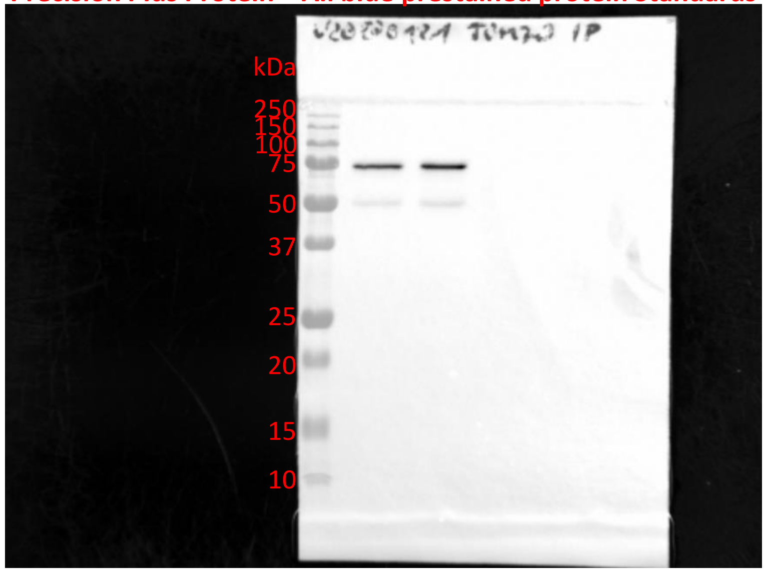

Figure 5 – SOD2 blot

Precision Plus Protein™ All blue prestained protein Standards

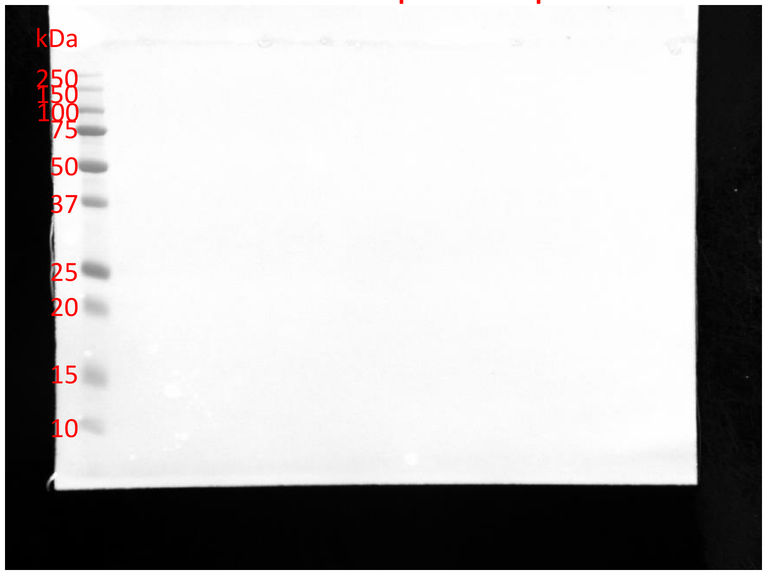

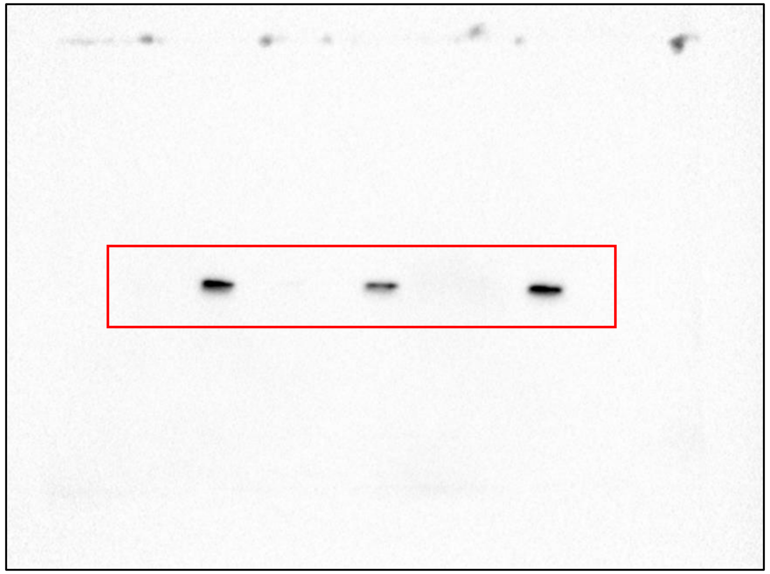

Precision Plus Protein™ All blue prestained protein Standards

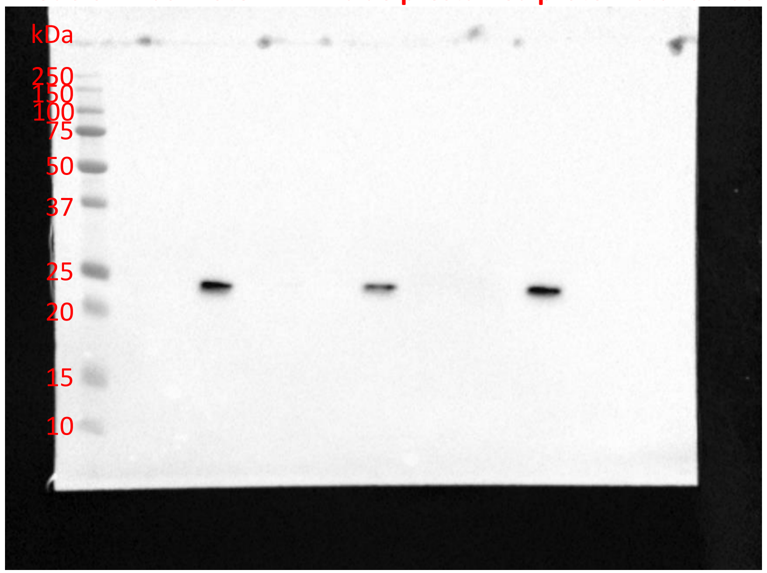

Figure 5 – Actin blot

Precision Plus Protein™ All blue prestained protein Standards

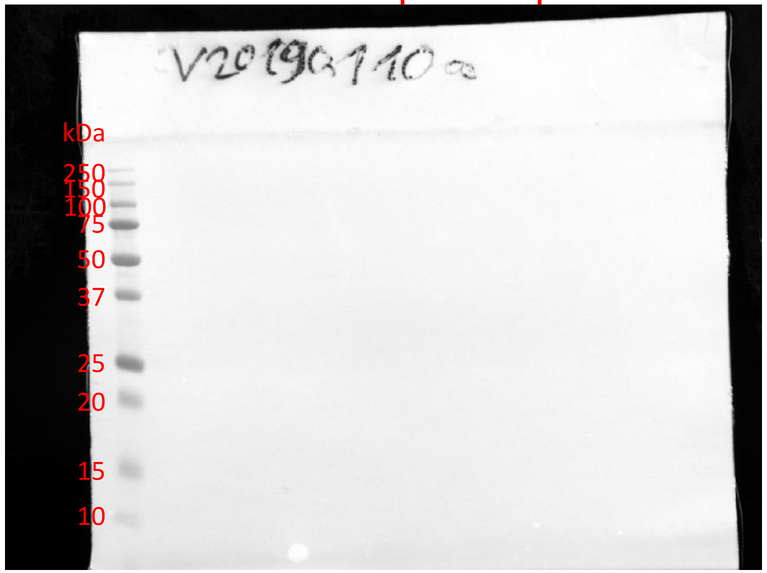

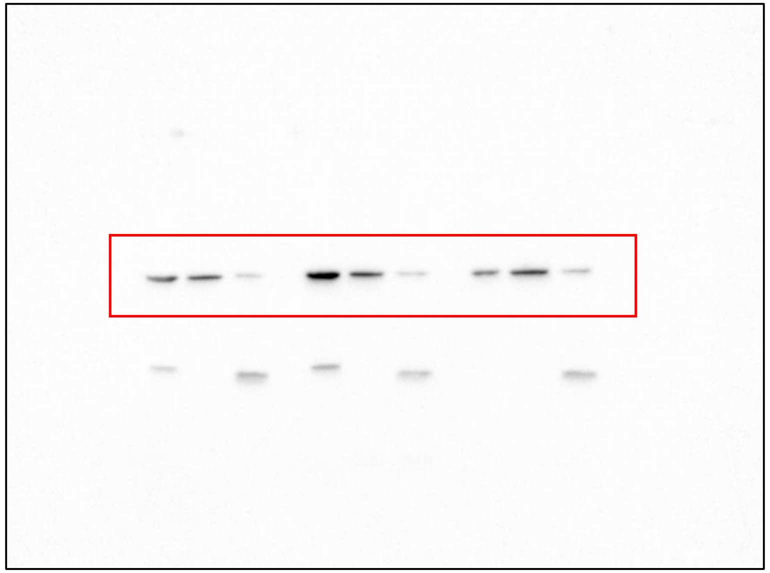

Precision Plus Protein™ All blue prestained protein Standards

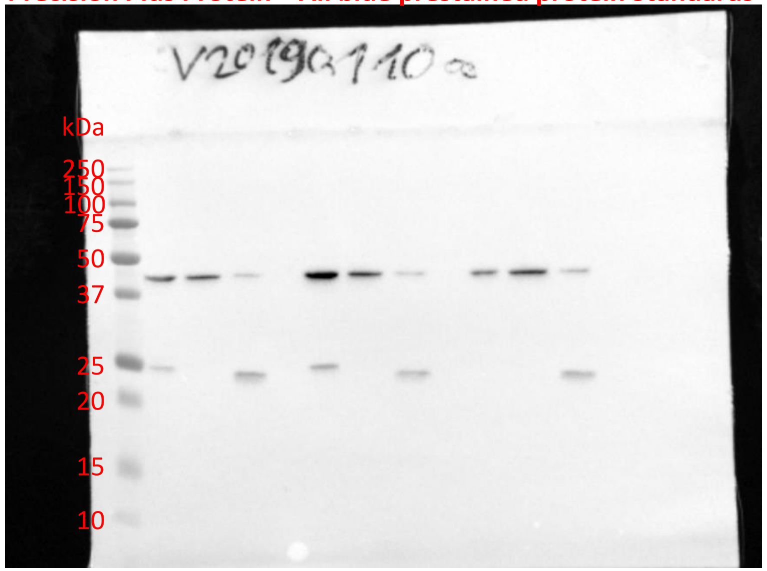

Figure 5 – PARP blot

Precision Plus Protein™ All blue prestained protein Standards

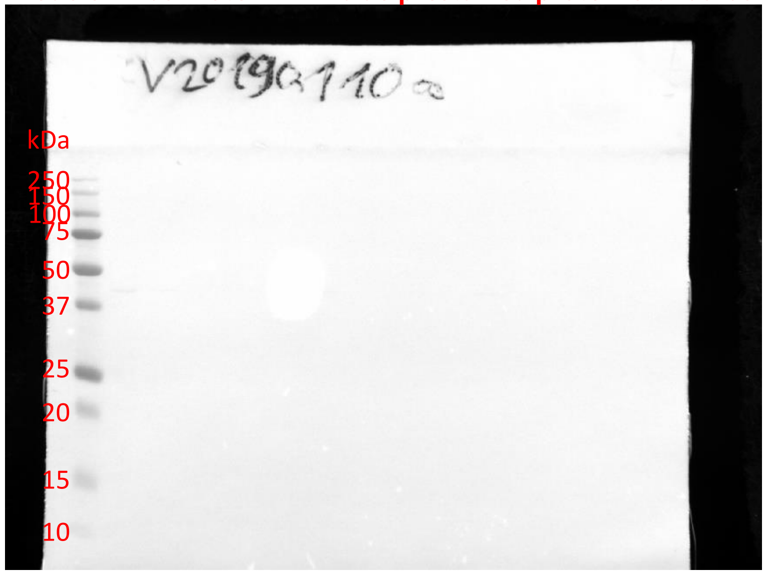

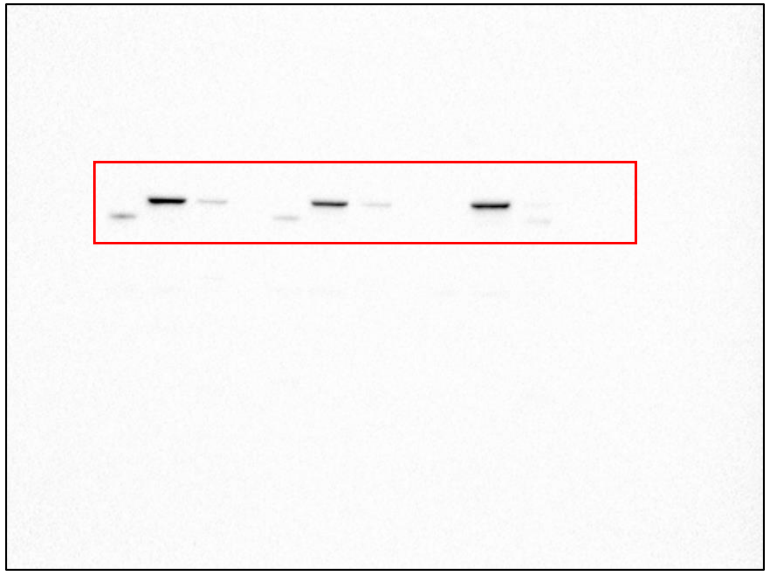

Precision Plus Protein™ All blue prestained protein Standards

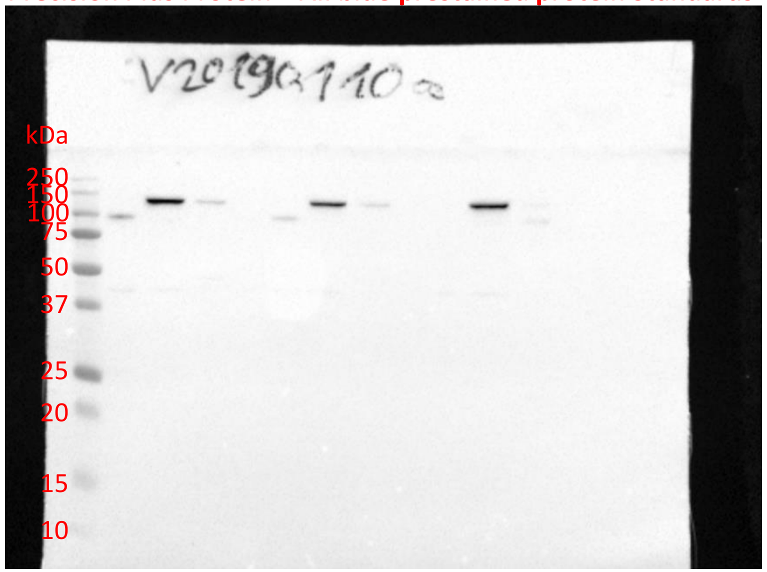

Precision Plus Protein™ All blue prestained protein Standards

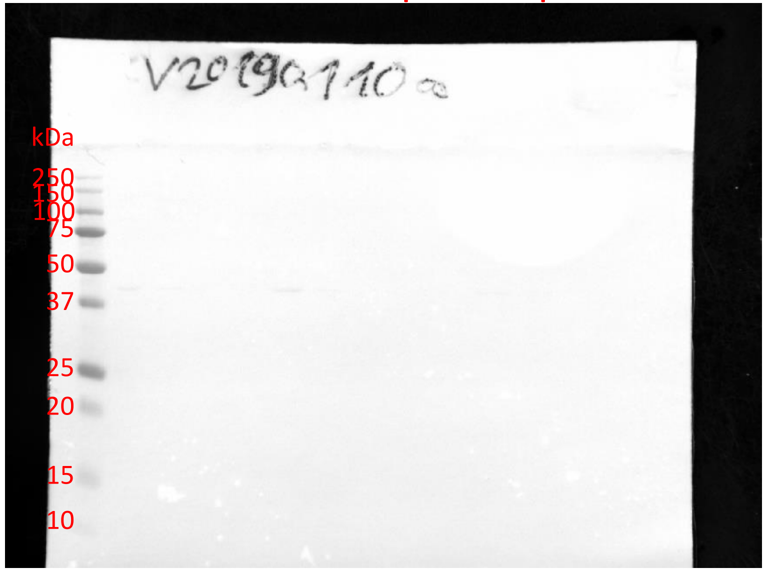

Figure 5 – Tom70 blot

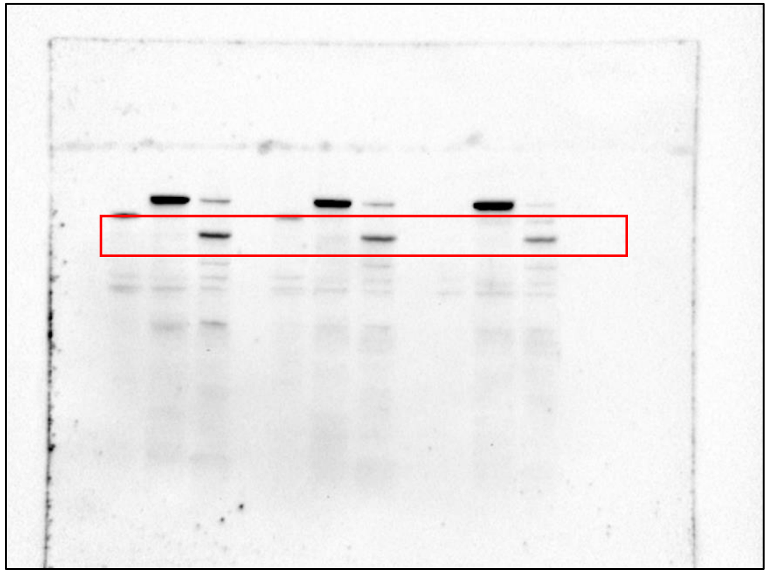

Precision Plus Protein™ All blue prestained protein Standards

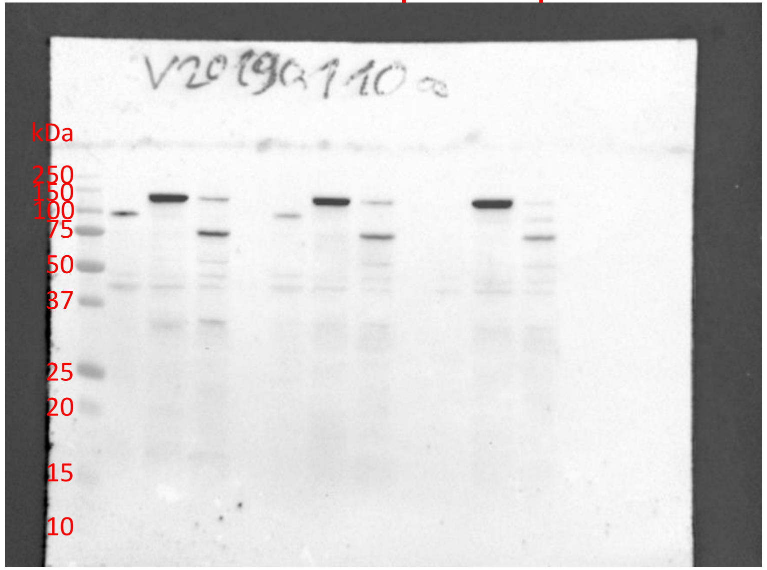

Precision Plus Protein™ All blue prestained protein Standards

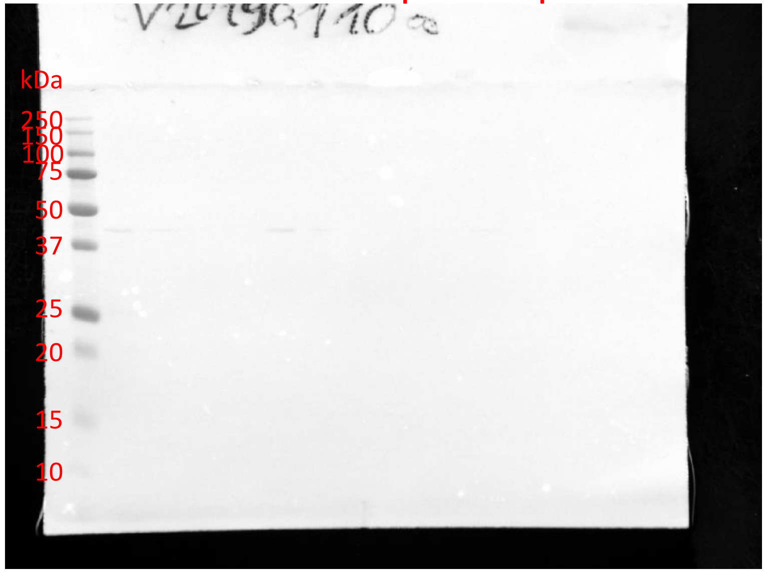

Figure 5 – RL2 blot

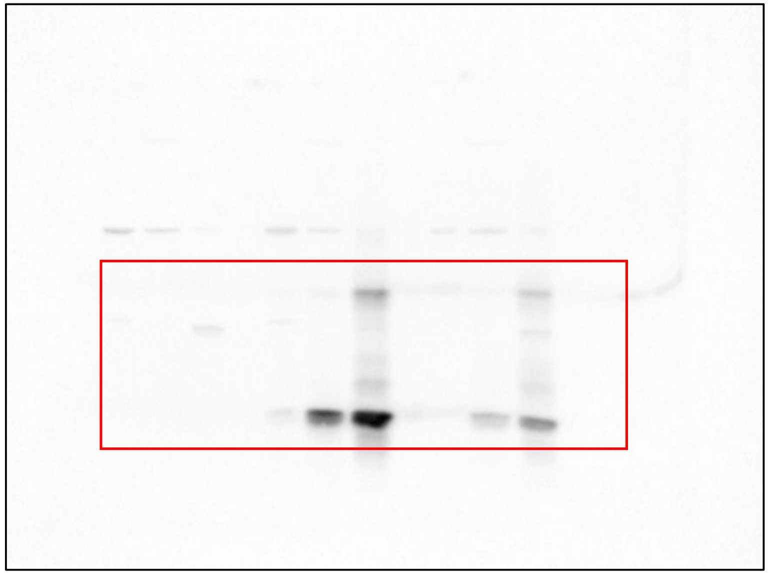

Precision Plus Protein™ All blue prestained protein Standards

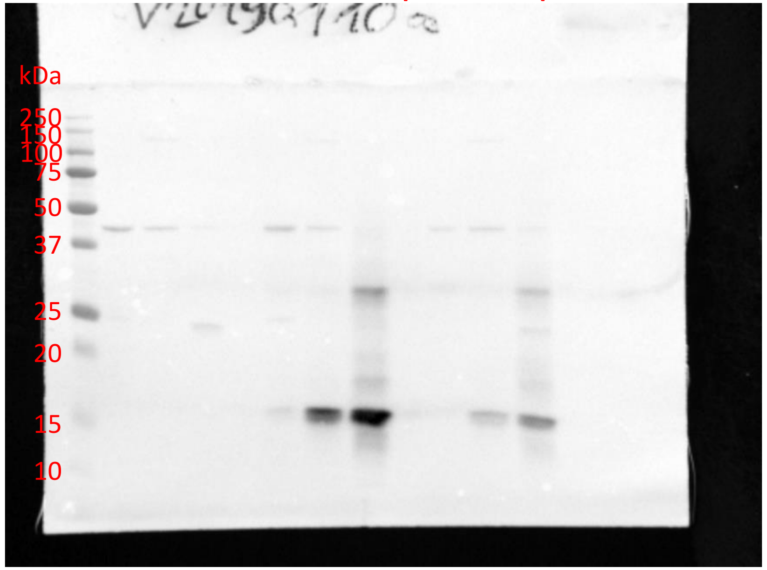

Figure 5 – GAPDH blot

Precision Plus Protein™ All blue prestained protein Standards

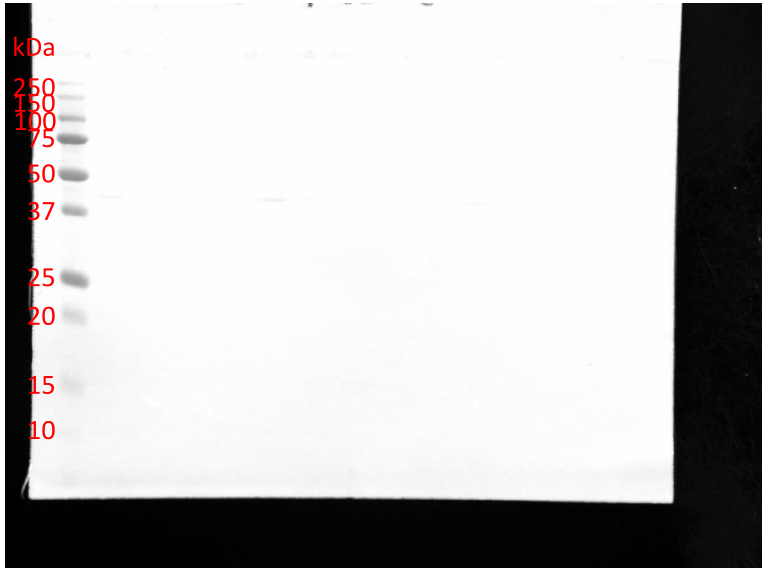

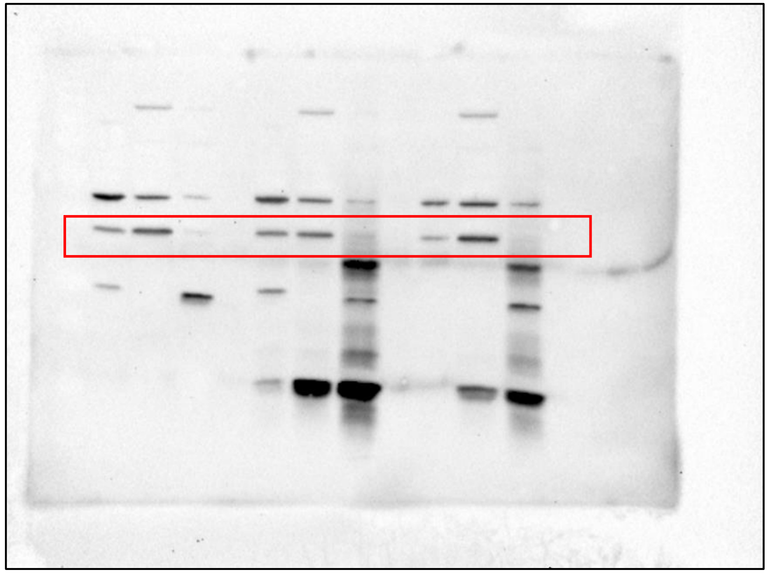

Precision Plus Protein™ All blue prestained protein Standards

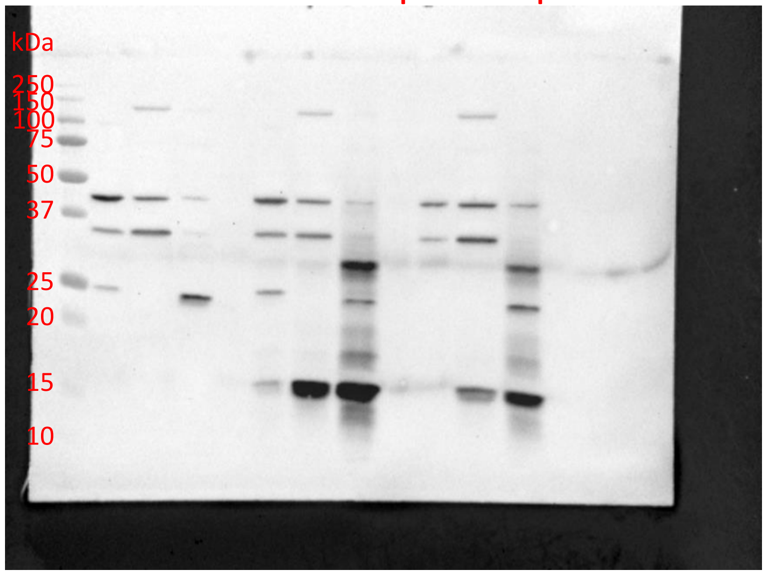

Figure 5 – AIF blot

Precision Plus Protein™ All blue prestained protein Standards

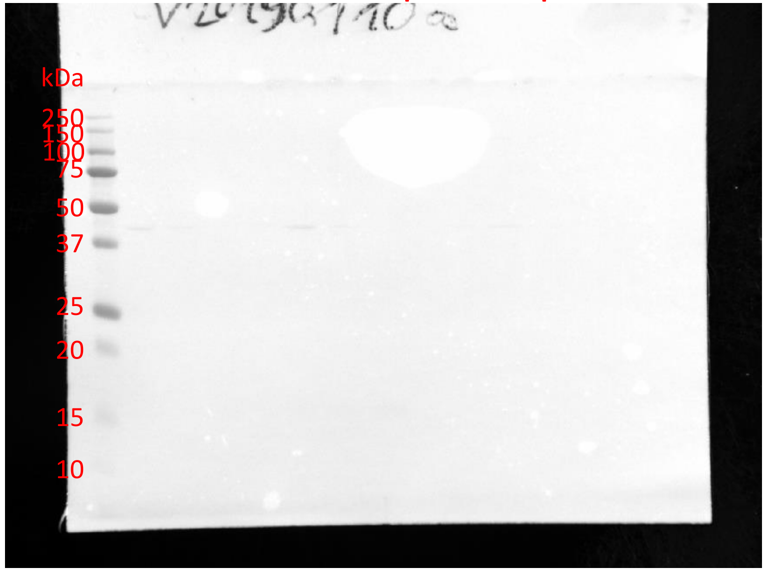

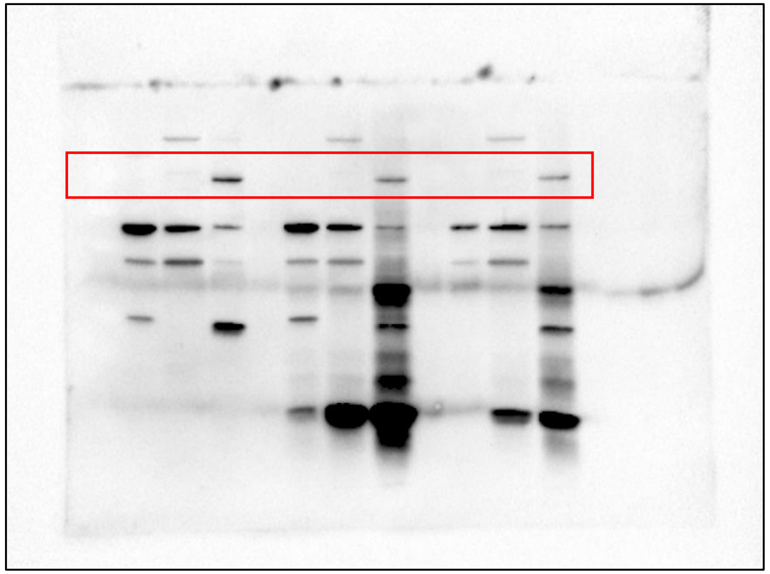

Precision Plus Protein™ All blue prestained protein Standards

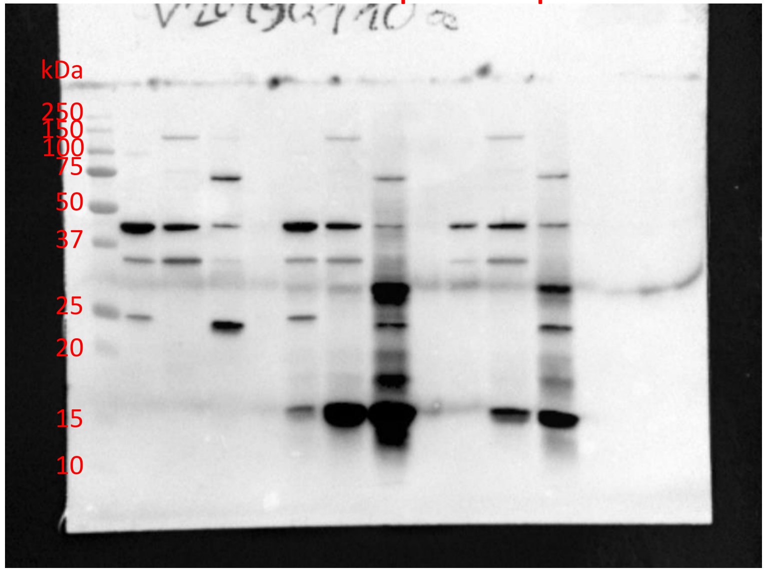

Figure 7C, MDA-MB-231 – Tom70 blot

Precision Plus Protein™ All blue prestained protein Standards

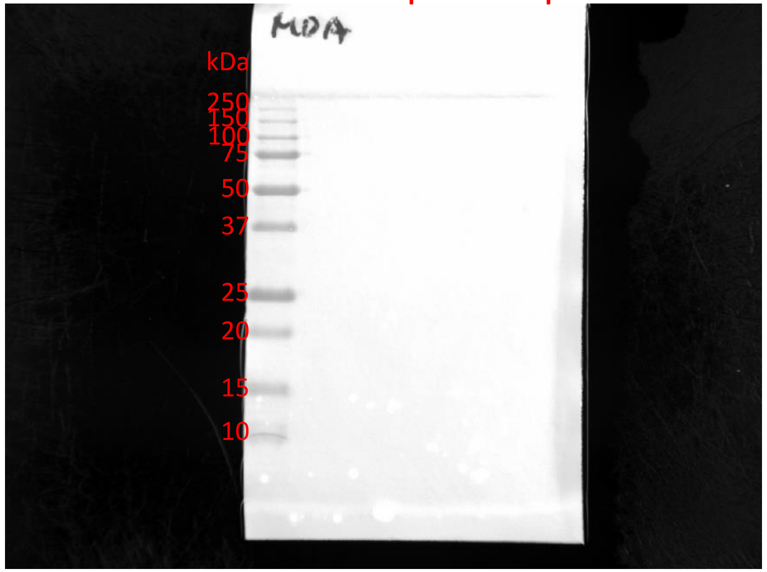

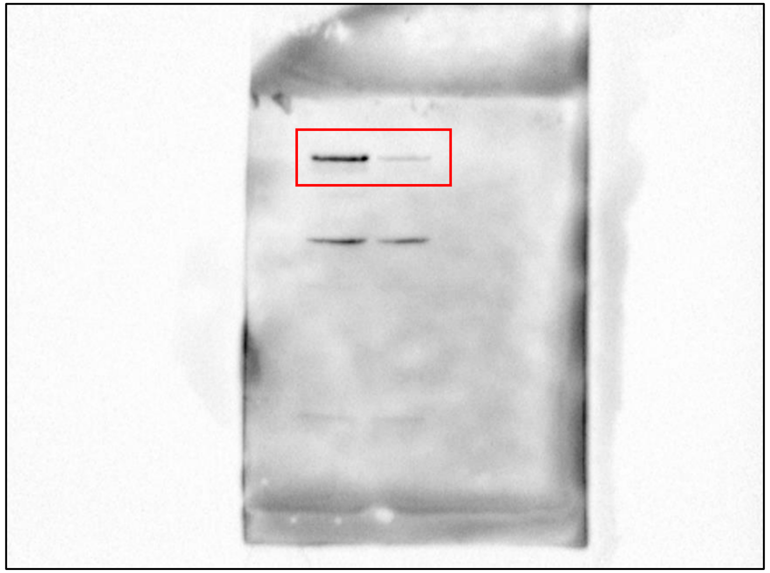

Precision Plus Protein™ All blue prestained protein Standards

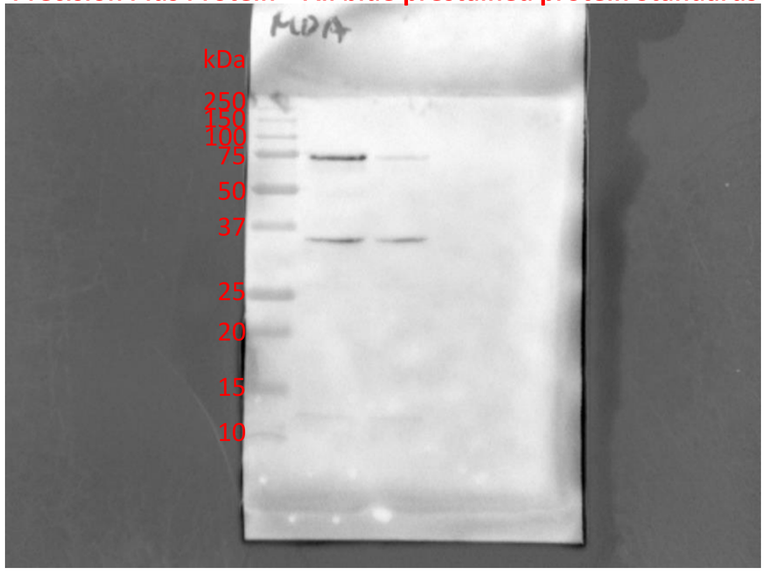

Figure 7C, MDA-MB-231 – Actin blot

Precision Plus Protein™ All blue prestained protein Standards

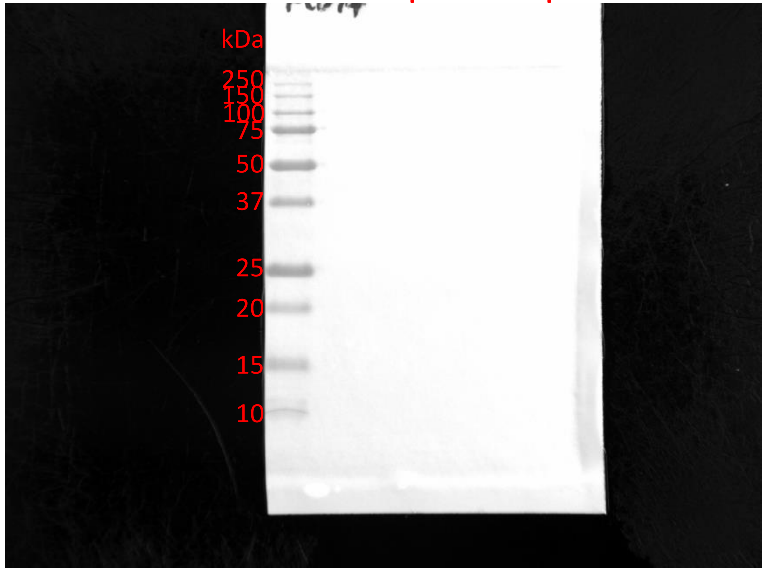

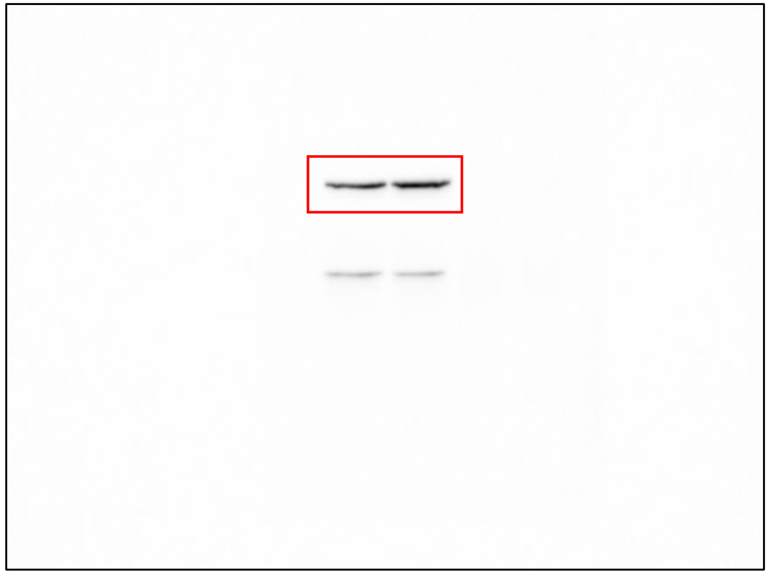

Precision Plus Protein™ All blue prestained protein Standards

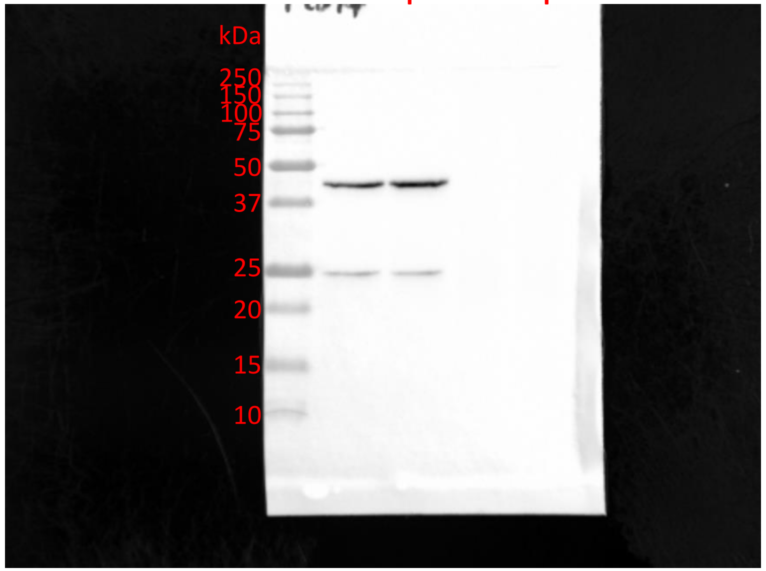

Figure 7C, MCF-7 – Tom70 blot

Precision Plus Protein™ All blue prestained protein Standards

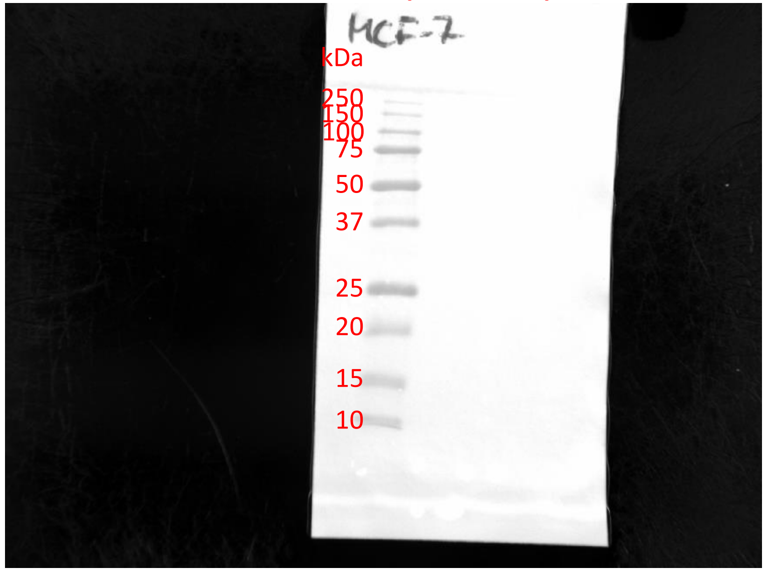

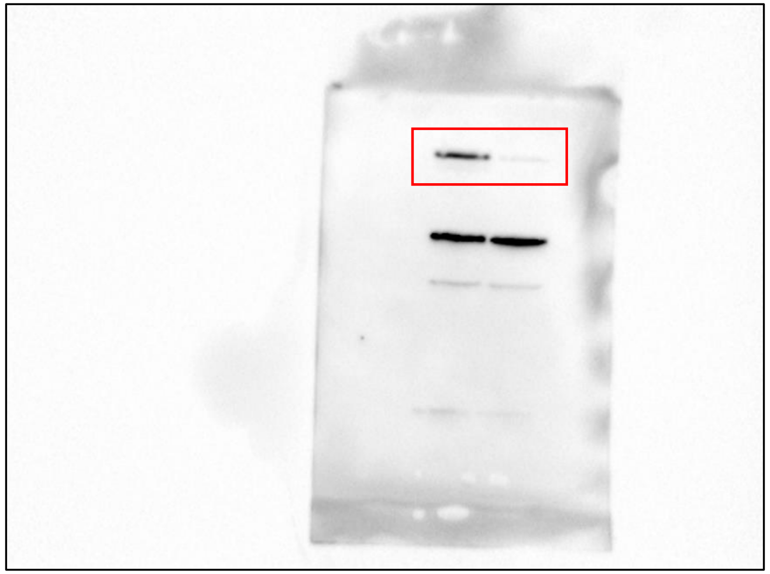

Precision Plus Protein™ All blue prestained protein Standards

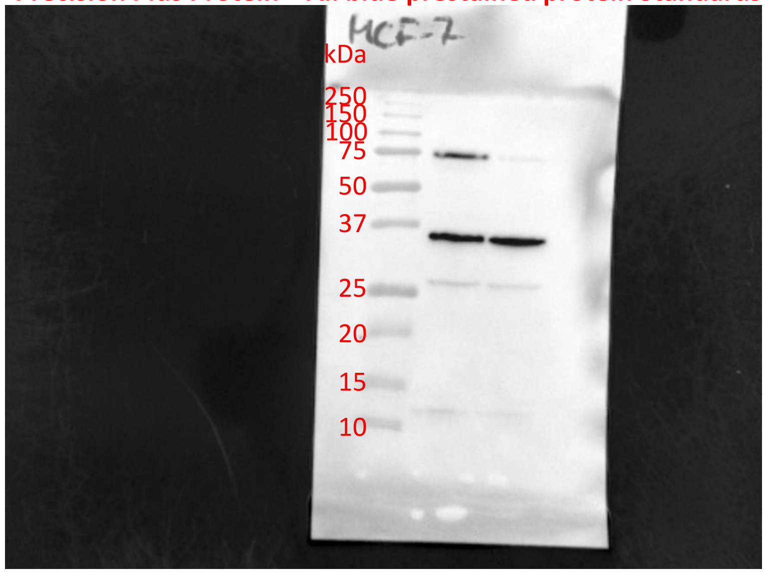

Figure 7C, MCF-7 – Actin blot

Precision Plus Protein™ All blue prestained protein Standards

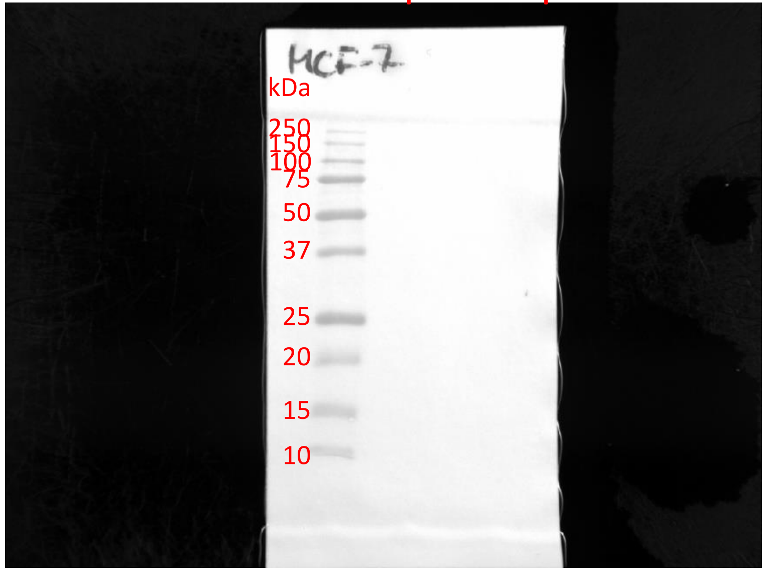

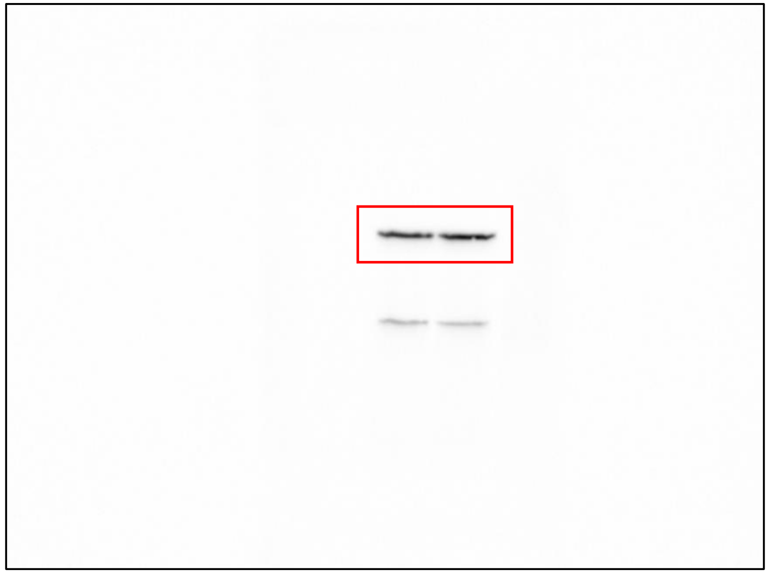

Precision Plus Protein™ All blue prestained protein Standards

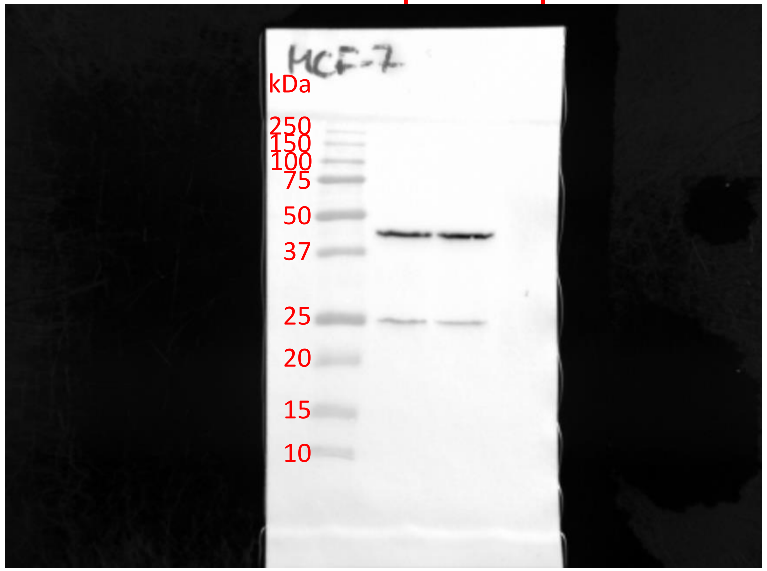

Supplement: Supplementary file 1 [file cancers-12-01427-s001.zip › cancers-806651-v1-suppl/cancers-806651-suppl-v1-the whole western blot.pdf]
