# Supplementary material for: The Recombinant Fragment of Human κ-Casein Induces Cell Death by Targeting the Proteins of Mitochondrial Import in Breast Cancer Cells
Source: Cancers (Basel). 2020 May 31;12(6):1427. doi: 10.3390/cancers12061427 (PMC7352597; doi:10.3390/cancers12061427)
Supplement: Supplementary file 1 [file cancers-12-01427-s001.zip › cancers-806651-v1-suppl/cancers-806651-suppl-v1.pdf]

The recombinant fragment of human κ-Casein induces cell death by targeting the proteins of mitochondrial import in breast cancer cells

Max Richter[1*], Fabian Wohlfromm[1*], Thilo Kähne[2], Hannes Bongartz[3], Kamil Seyrek[1], Yuriy Kit[4], Olga Chinak[5], Vladimir Richter[5#], Olga Koval[5#], Inna Lavrik[1#§]

[1]Department of Translational Inflammation Research, Medical Faculty, Otto von Guericke University, Magdeburg, Germany

[2]Institute of Experimental Internal Medicine, Medical Faculty, Otto von Guericke University, Magdeburg, Germany

[3]Institute of Biology, Systems Biology, Faculty of Natural Sciences, Otto von Guericke University, Magdeburg, Germany

[4]Institute of Cell Biology, L'viv, Ukraine

[5]Department of Biotechnology, Institute of Chemical Biology and Fundamental Medicine, SB RAS, Novosibirsk, Russia

[*-]equal contribution [#]- equal contribution [§-]Corresponding author

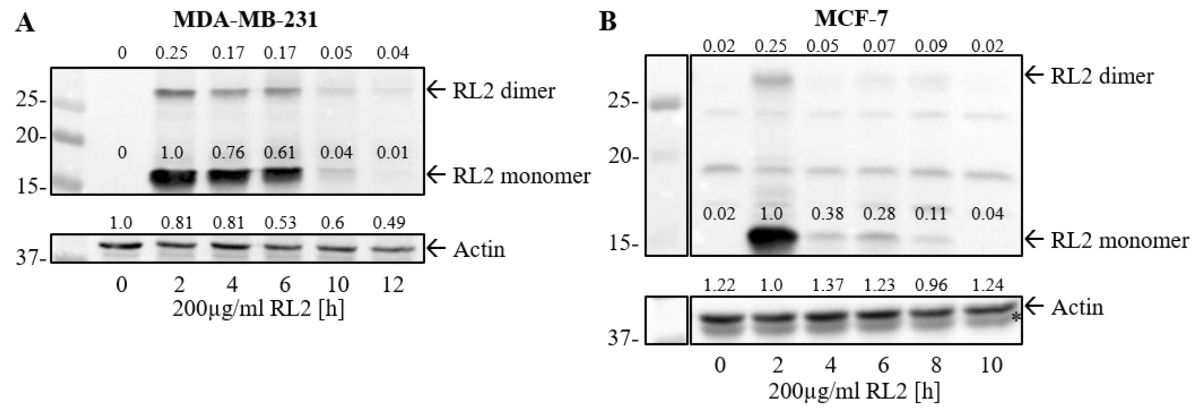

Supplementary Figure 1. Relative Western Blot quantifications of Figure 1B and 1C. Quantification of protein band intensities were performed for three independent experiments with ImageLab 5.1beta (Bio-Rad). One representative quantification is shown here. Probes with a value of 1.0 served as reference for that specific protein. For more information see Figure 1.

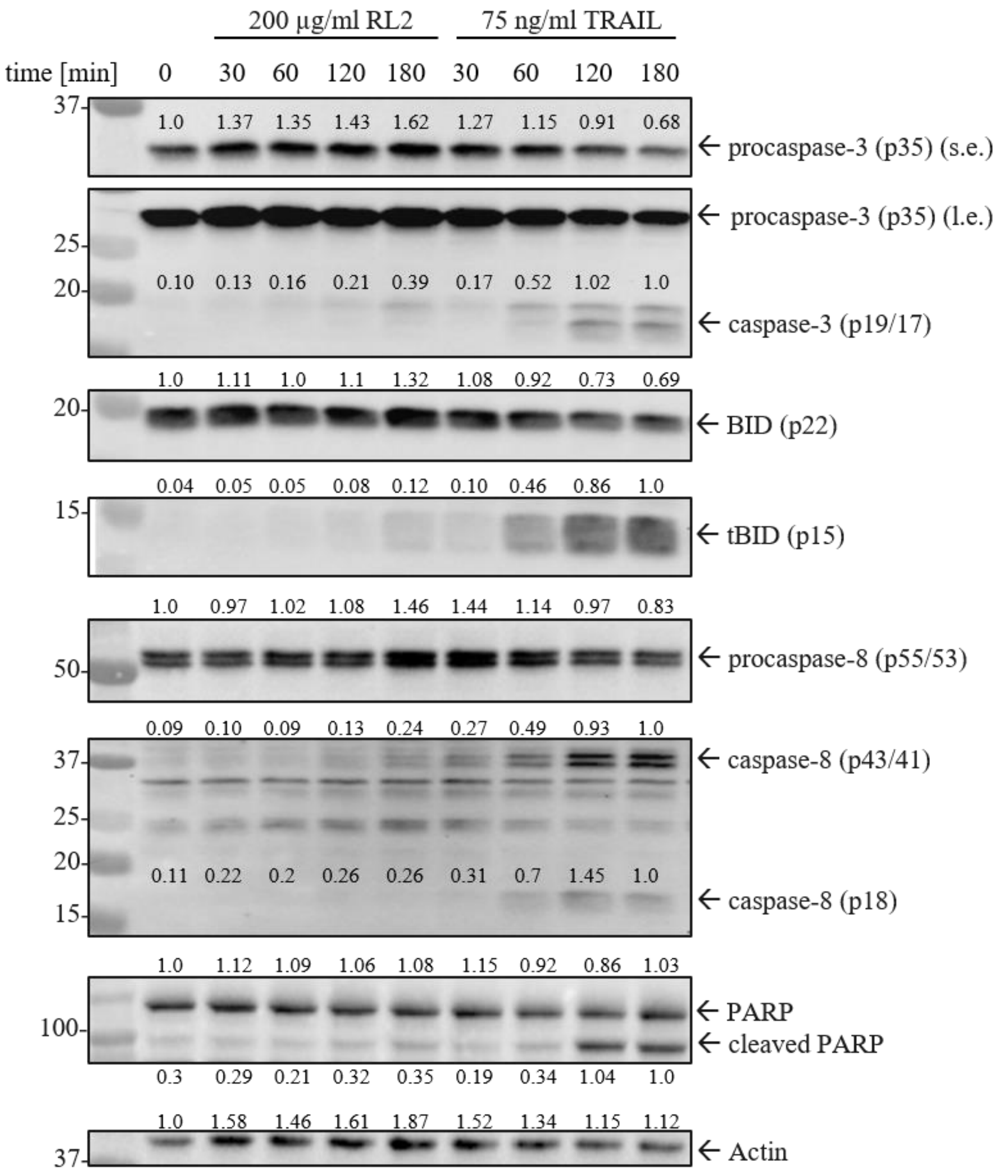

Supplementary Figure 2. Relative Western Blot quantifications of Figure 3.
Quantification of protein band intensities was performed with ImageLab 5.1beta (Bio-Rad). Probes with a value of 1.0 served as reference for that specific protein. For more information see Figure 3.

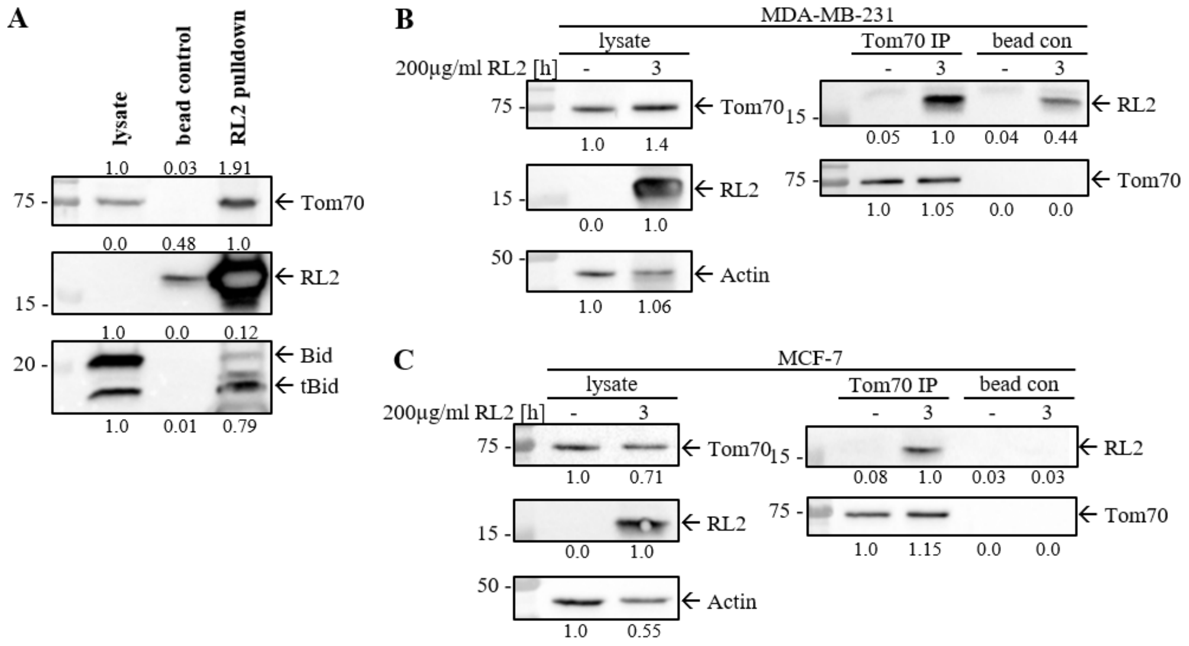

Supplementary Figure 3. Relative Western Blot quantifications of Figure 4A, 4B and 4C. Quantification of protein band intensities was performed with ImageLab 5.1beta (Bio-Rad). Probes with a value of 1.0 served as reference for that specific protein. For more information see Figure 4.

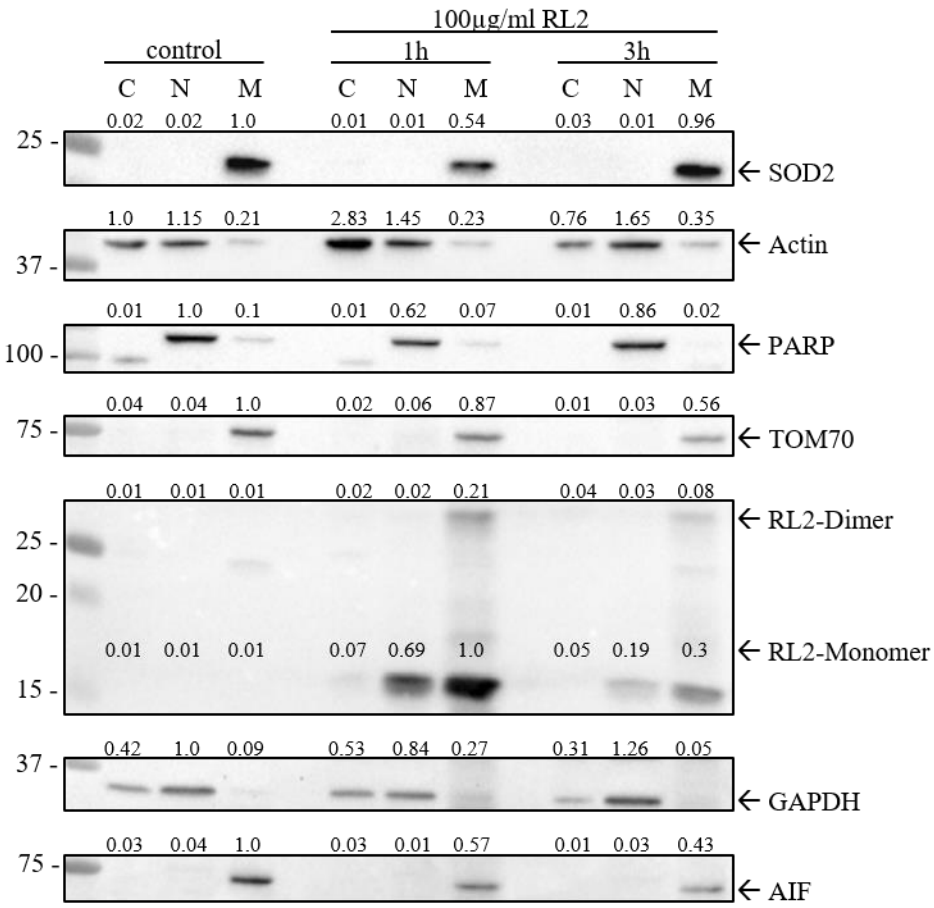

Supplementary Figure 4. Relative Western Blot quantifications of Figure 5.
Quantification of protein band intensities were performed for three independent experiments with ImageLab 5.1beta (Bio-Rad). One representative quantification is shown here. Probes with a value of 1.0 served as reference for that specific protein. For more information see Figure 5.

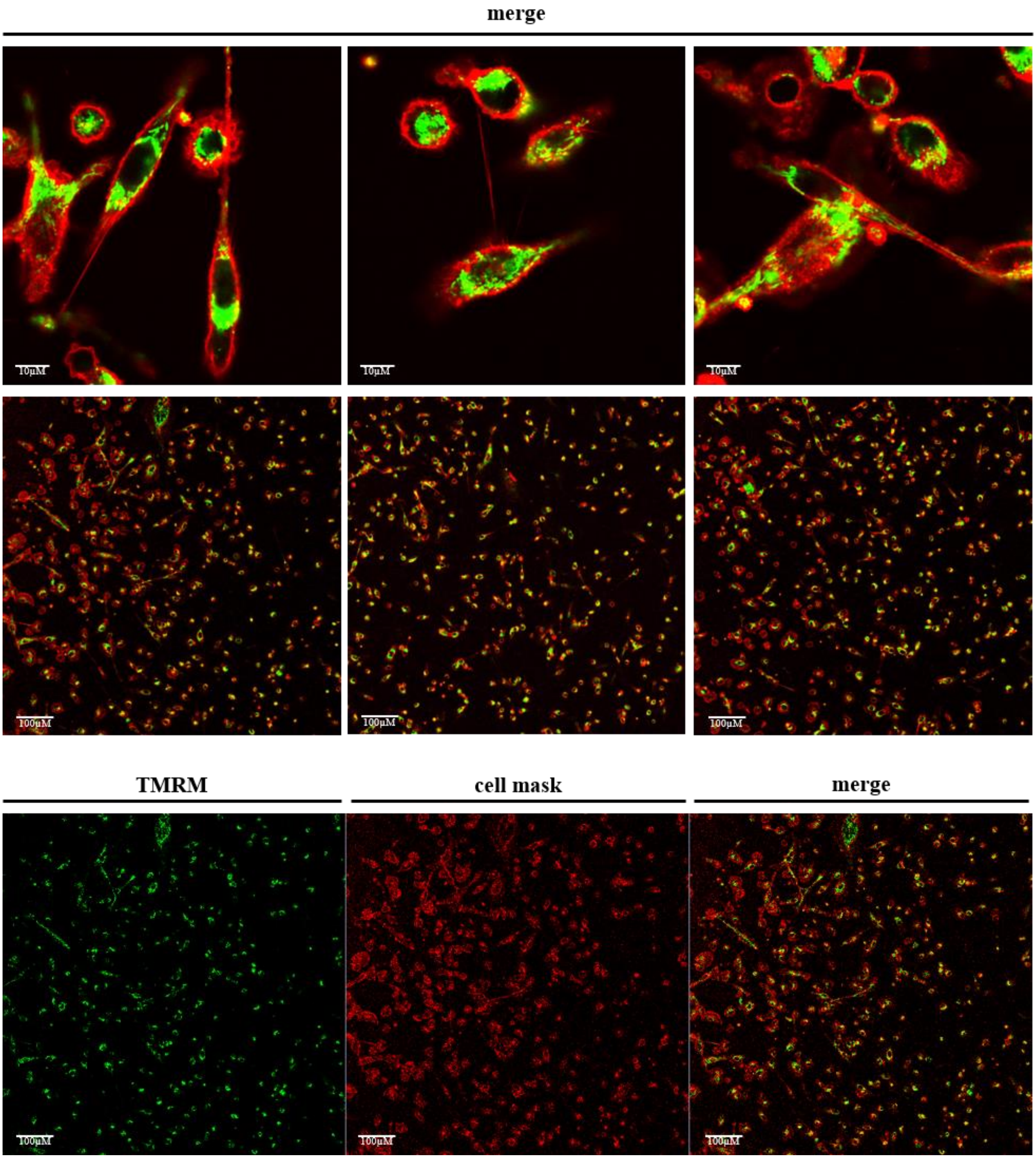

Supplementary Figure 5 A. TMRM-indicated mitochondrial membrane potential loss of MDA-MB-231 cells. MDA-MB-231 cells were stained with 20 µM TMRM (MitoProbe™ TMRM Kit for Flow Cytometry, M20036, Invitrogen) and 5mg/ml cell membrane stain (CellMask™ Deep Red Plasma membrane Stain, C10046, ThermoFisher). Loss of mitochondrial membrane potential was indicated by a reduced TMRM signal. Membrane- and TMRM-stained cells are shown in merge for single cells (top), population (middle) and for single channels (bottom). (A) Unstimulated MDA-MB-231 cells were used as negative control.

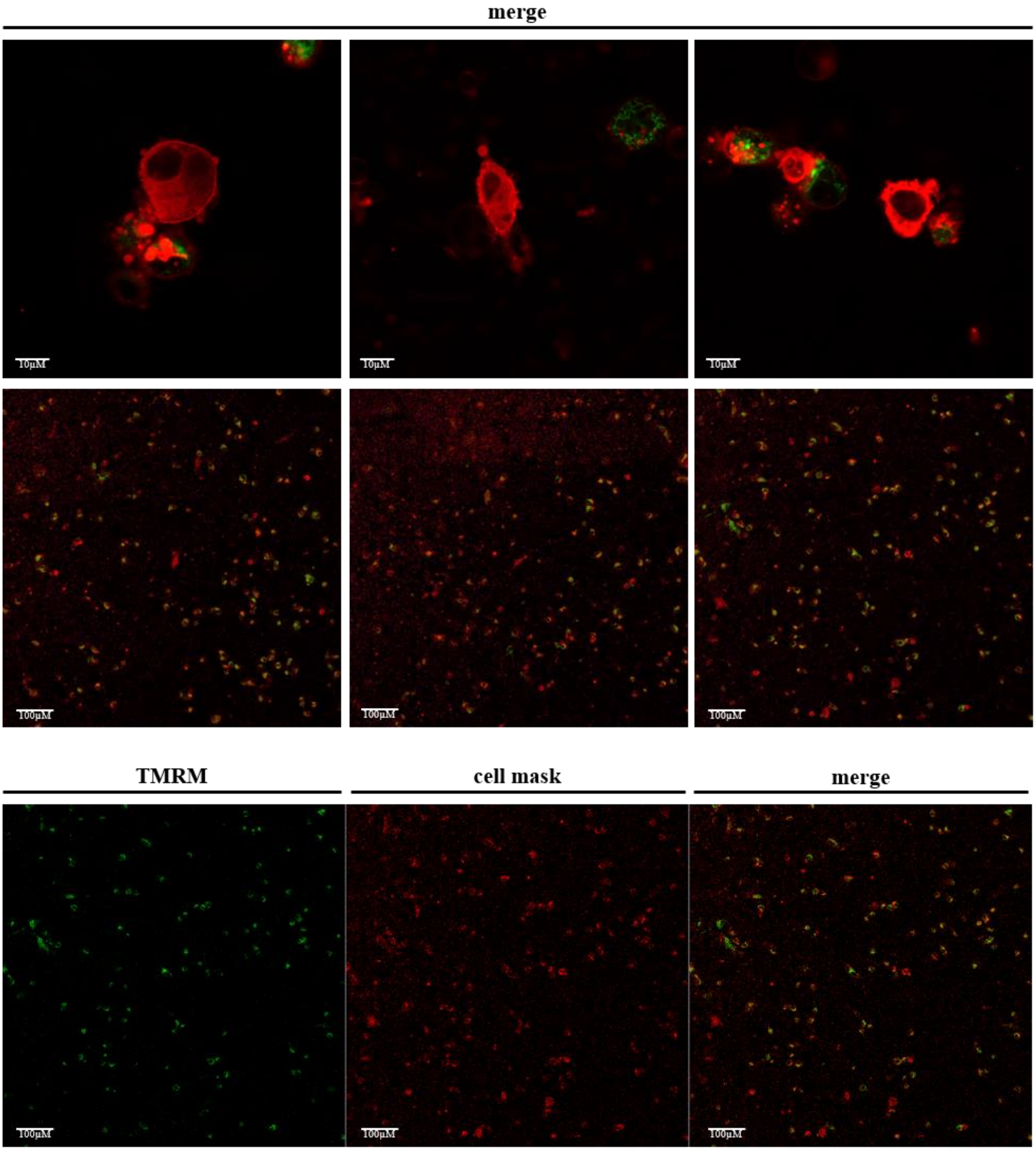

Supplementary Figure 5 B. TMRM-indicated mitochondrial membrane potential loss of MDA-MB-231 cells. MDA-MB-231 cells were stained with 20 µM TMRM (MitoProbe™ TMRM Kit for Flow Cytometry, M20036, Invitrogen) and 5mg/ml cell membrane stain (CellMask™ Deep Red Plasma membrane Stain, C10046, ThermoFisher). Loss of mitochondrial membrane potential was indicated by a reduced TMRM signal. Membrane- and TMRM-stained cells are shown in merge for single cells (top), population (middle) and for single channels (bottom). (B) MDA-MB-231 cells were prior stimulated for six hours with 300 µg/ml RL2.

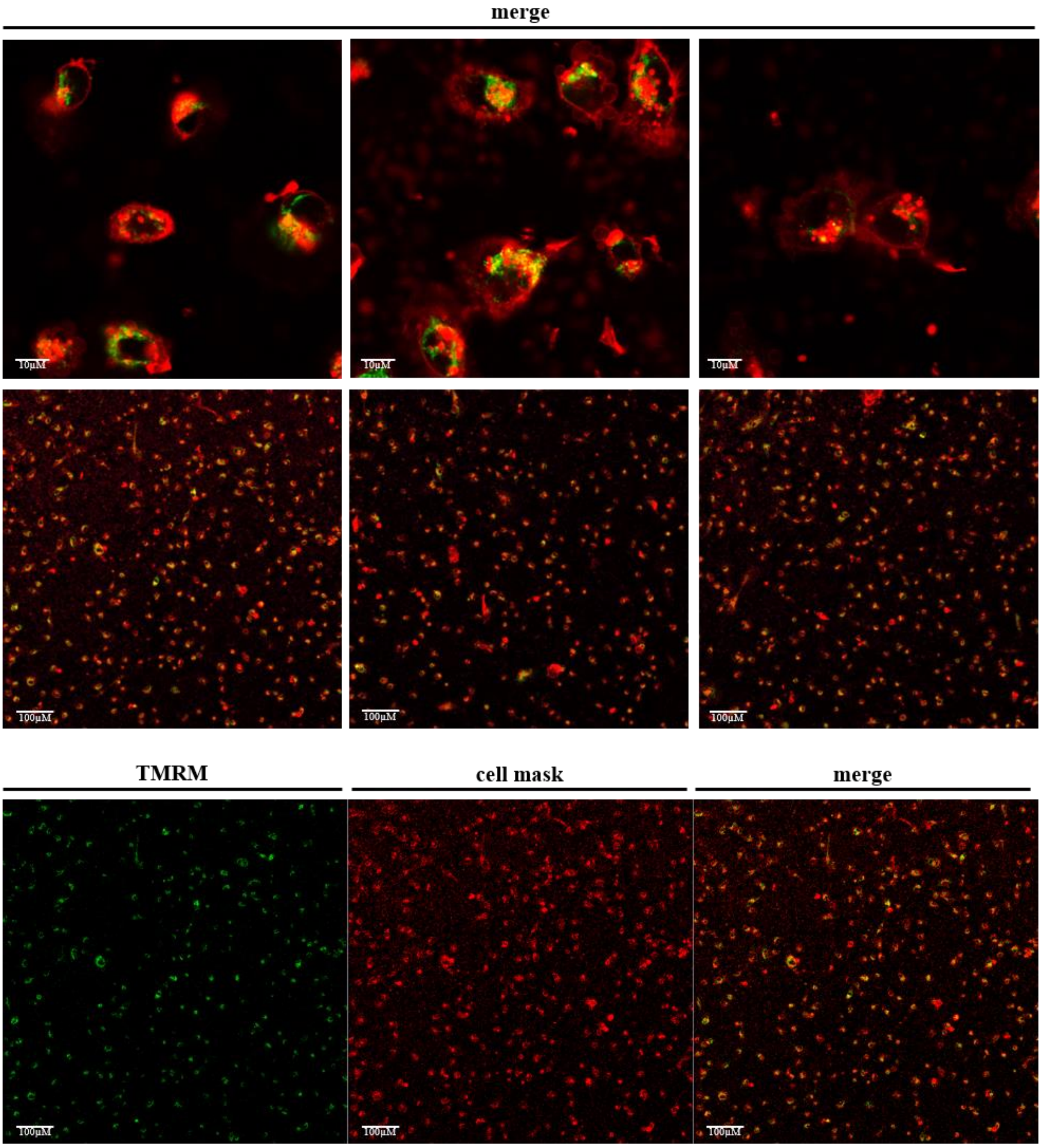

Supplementary Figure 5 C. TMRM-indicated mitochondrial membrane potential loss of MDA-MB-231 cells. MDA-MB-231 cells were stained with 20 µM TMRM (MitoProbe™ TMRM Kit for Flow Cytometry, M20036, Invitrogen) and 5mg/ml cell membrane stain (CellMask™ Deep Red Plasma membrane Stain, C10046, ThermoFisher). Loss of mitochondrial membrane potential was indicated by a reduced TMRM signal. Membrane- and TMRM-stained cells are shown in merge for single cells (top), population (middle) and for single channels (bottom). (C) MDA-MB-231 cells were prior stimulated for 24 hours with 300 µg/ml RL2.

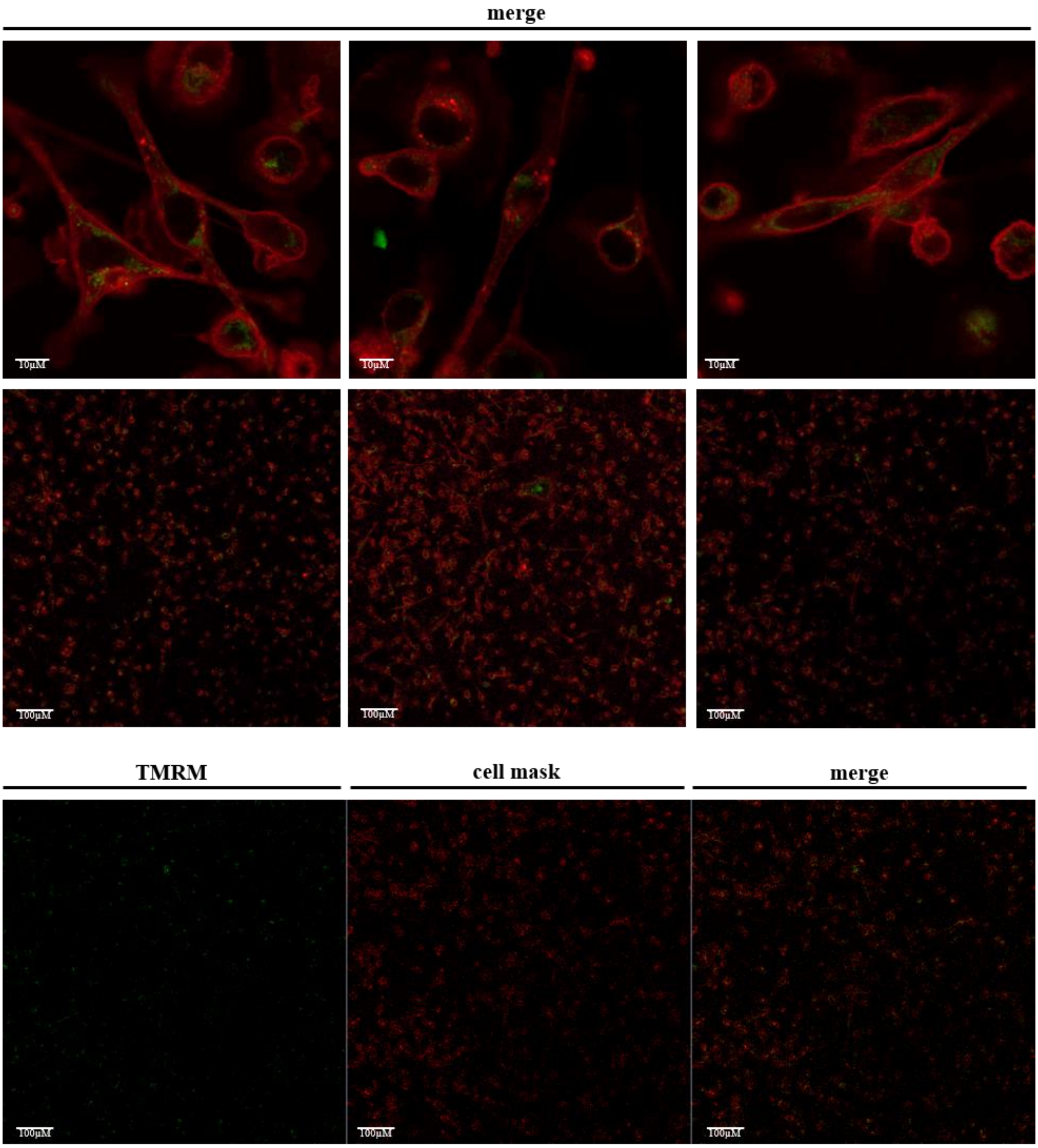

Supplementary Figure 5 D. TMRM-indicated mitochondrial membrane potential loss of MDA-MB-231 cells. MDA-MB-231 cells were stained with 20 µM TMRM (MitoProbe™ TMRM Kit for Flow Cytometry, M20036, Invitrogen) and 5mg/ml cell membrane stain (CellMask™ Deep Red Plasma membrane Stain, C10046, ThermoFisher). Loss of mitochondrial membrane potential was indicated by a reduced TMRM signal. Membrane- and TMRM-stained cells are shown in merge for single cells (top), population (middle) and for single channels (bottom). (D) MDA-MB-231 cells were prior stimulated for 5 min with CCCP.

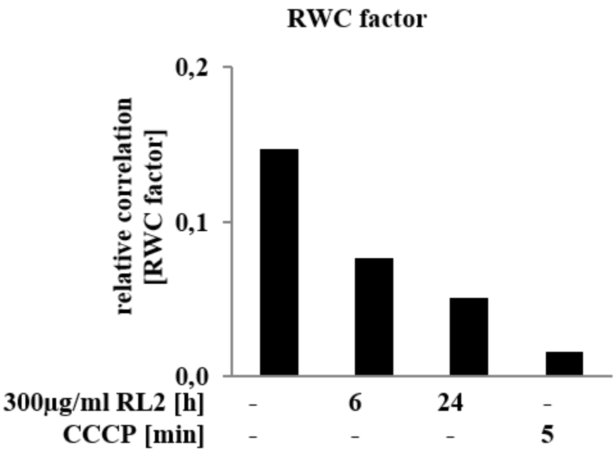

Supplementary Figure 5 E. Quantification of mitochondrial membrane potential loss. Three independent cell overview images for indicated stimulations were quantitatively analyzed for correlation of TMRM and red membrane stain positive cells with cell image analysis software (CellProfiler, Stable 3.1.8). Induced correlation is shown by Rank-Weighted Co-localization (RWC) factor.

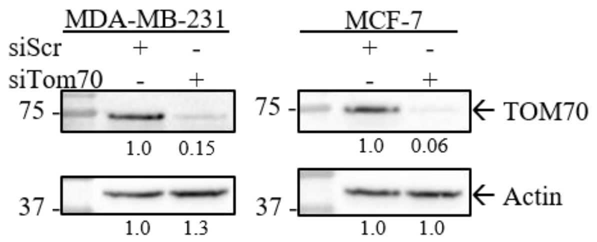

Supplementary Figure 6. Relative Western Blot quantifications of Figure 7. Quantification of protein band intensities were performed for three independent experiments with ImageLab 5.1beta (Bio-Rad). One representative quantification is shown here. Probes with a value of 1.0 served as reference for that specific protein. For more information see Figure 7.

Supplementary Table 1 - Absolute values of unique peptides and coverages of potential RL2-interacting proteins identified by mass spectrometry. The analysis of potential RL2-interacting proteins in Figure 4B, are based on the absolute values shown here. The abbreviations n_1, n_2 and n_3 indicate absolute values for every experimental replicate.

protein	ID	RL2-pulldown						control					
		unique peptides			coverage [%]			unique peptides			coverage [%]		
		n_1	n_2	n_3	n_1	n_2	n_3	n_1	n_2	n_3	n_1	n_2	n_3
2AAA	P30153	23	25	23	60	62	63	4	7	2	12	18	13
ACTN1	P12814	37	34	38	75	75	76	10	12	4	35	42	20
CASK	P07498	88	85	84	60	59	59	21	7	-	57	45	-
CH60	P10809	58	58	45	71	77	72	28	34	10	64	67	27
CXA1	P12074	2	-	-	43	-	-	-	-	-	-	-	-
EFTU	P49411	18	21	23	51	57	59	9	23	6	27	60	20
GRP75	P38646	23	22	22	46	43	44	15	19	4	37	37	15
HCD2	Q99714	9	5	7	62	41	54	1	9	3	10	58	25
HNRPF	P52597	20	21	18	62	60	62	12	7	4	52	34	22
HNRPL	P14866	20	14	17	60	47	54	6	4	3	29	17	17
MIF	P14174	3	3	3	65	36	36	1	8	2	8	70	43
MPPA	Q10713	12	13	17	43	39	49	-	-	-	-	-	-
MYL6	P60660	11	12	8	84	84	58	1	16	4	16	85	36
PPIA	P62937	11	12	11	67	67	67	3	19	3	19	67	26
PTBP1	P26599	16	18	16	66	69	66	10	9	8	44	37	37
PYR1	P27708	81	99	95	60	66	65	19	9	4	21	9	6
RLA1	P05386	2	3	3	67	88	88	4	3	2	88	82	52
RLA2	P05387	5	6	4	84	84	84	6	7	-	85	85	-
RS3	P23396	14	7	13	64	28	63	12	12	1	56	56	6
RT17	Q9Y2R5	4	3	4	52	36	54	1	-	-	15	-	-
SDHA	P31040	18	17	22	48	43	52	-	10	5	-	26	18
TBB5	P07437	7	7	10	92	92	92	7	5	3	89	91	53
TIM8A	O60220	4	4	4	68	67	64	-	-	-	-	-	-
TIM8B	Q9Y5J9	3	3	3	42	42	42	-	-	-	-	-	-
TOM70	O94826	15	17	18	41	47	50	-	-	-	-	-	-

Supplementary Table 2 - Absolute values of unique peptides and coverages of all mitochondrial proteins identified by mass spectrometry. Abbreviations n_1, n_2 and n_3 indicate absolute values for every experimental replicate.

ID	RL2-pulldown						control					
	unique peptides			coverage [%]			unique peptides			coverage [%]		
	n_1	n_2	n_3	n_1	n_2	n_3	n_1	n_2	n_3	n_1	n_2	n_3
P00505	-	-	-	-	-	-	-	3	-	-	12	-
Q9UKU7	-	-	2	-	-	5	-	-	-	-	-	-
P49748	-	-	-	-	-	-	-	-	1	-	-	4
Q99798	-	1	-	-	2	-	-	-	2	-	-	6
Q9Y305	-	5	12	-	14	31	-	9	3	-	24	10
O14561	2	3	3	19	22	22	-	3	-	-	24	-
Q9H6R3	-	-	-	-	-	-	-	1	1	-	2	2
Q53H12	3	-	-	12	-	-	7	-	-	27	-	-
O95831	-	1	5	-	2	16	-	-	-	-	-	-
Q92667	-	2	-	-	4	-	-	-	1	-	-	3
P30837	-	3	-	-	10	-	-	-	2	-	-	5
P05091	2	-	3	4	-	8	-	3	1	-	13	3
O75947	-	-	-	-	-	-	4	4	1	30	38	13
P56385	-	-	-	-	-	-	-	1	-	-	14	-
P18859	-	-	-	-	-	-	3	-	-	44	-	-
P25705	-	12	13	-	29	33	19	24	4	41	48	10
P06576	11	11	8	37	39	31	-	-	6	-	-	28
P30049	-	-	-	-	-	-	-	2	-	-	14	-
Q8N5M1	-	6	7	-	25	49	-	-	-	-	-	-
P36542	1	-	2	4	-	8	4	-	-	21	-	-
P56134	-	-	-	-	-	-	-	2	1	-	26	14
P48047	-	-	-	-	-	-	-	7	1	-	46	8
O14874	-	-	3	-	-	13	-	-	-	-	-	-
Q02338	1	1	2	3	3	10	-	-	1	-	-	8
O43684	-	6	-	-	23	-	-	-	-	-	-	-
Q07021	-	5	6	-	27	41	4	-	1	22	-	10
Q6UB35	1	-	1	2	-	2	-	-	-	-	-	-
P61604	-	2	1	-	19	12	-	6	-	-	60	-
P10809	58	-	-	71	-	-	28	34	10	64	67	27
O75390	-	-	-	-	-	-	-	5	-	-	18	-
O75153	27	14	-	29	16	-	4	-	-	5	-	-
Q9UJS0	9	8	10	24	22	18	-	-	-	-	-	-
P13073	3	4	4	20	26	26	4	2	-	26	14	-
P20674	4	7	6	41	47	47	-	2	-	-	23	-
P10606	-	6	5	-	40	39	-	-	3	-	-	24
O14548	-	1	45	-	29	72	-	-	-	-	-	-
P31327	-	-	-	-	-	1	-	-	-	-	-	-
P12074	2	-	-	43	-	-	-	-	-	-	-	-

P14406	2	2	2	28	28	28	-	-	-	-	-	-
P08574	4	2	2	18	10	10	-	-	1	-	-	7
Q9NR28	1	-	-	4	-	-	-	-	-	-	-	-
P00367	-	-	-	-	-	-	-	16	-	-	35	-
P49448	3	-	-	5	-	-	-	-	-	-	-	-
Q96HY7	-	-	1	-	-	1	-	-	-	-	-	-
Q9UBX3	-	2	6	-	12	24	-	-	1	-	-	8
P09622	-	-	-	-	-	-	-	1	1	-	2	3
Q96EY1	-	7	10	-	16	33	-	-	-	-	-	-
P33316	-	-	-	-	-	-	-	5	-	-	25	-
Q13011	-	-	-	-	-	-	-	3	-	-	10	-
P40939	4	2	1	12	6	4	-	8	6	-	20	16
P55084	-	-	-	-	-	-	-	2	-	-	8	-
P42126	-	-	-	-	-	-	-	2	1	-	9	4
O75521	-	-	-	-	-	-	-	7	2	-	26	15
P49411	18	21	23	51	57	59	9	23	6	27	60	20
O75616	8	6	-	31	24	-	-	-	-	-	-	-
P13804	-	-	-	-	-	-	-	3	2	-	16	13
Q9NYY8	-	-	1	-	-	2	-	-	-	-	-	-
P07954	-	-	-	-	-	-	-	-	8	-	-	28
Q92947	2	-	-	8	-	-	-	-	1	-	-	6
Q9H936	-	1	2	-	2	7	-	-	-	-	-	-
Q16775	-	-	-	-	-	-	-	2	-	-	8	-
P34897	-	-	-	-	-	-	-	9	1	-	29	8
P43304	-	-	-	-	-	-	-	-	3	-	-	5
P38646	23	22	22	46	43	44	15	19	4	37	37	15
Q9HAV7	-	-	-	-	-	-	-	1	-	-	5	-
P00390	-	-	-	-	-	-	-	2	-	-	8	-
Q16836	-	-	-	-	-	-	-	4	1	-	27	7
P36551	-	-	2	-	-	6	-	-	4	-	-	13
O43464	2	1	2	8	3	11	-	-	-	-	-	-
P50213	-	-	-	-	-	-	-	2	-	-	9	-
Q9H2U2	-	-	-	-	-	-	-	12	3	-	51	15
Q9H1K1	-	-	-	-	-	-	-	-	1	-	-	5
P54819	-	-	-	-	-	-	-	3	1	-	14	4
Q9UIJ7	-	-	-	-	-	-	-	2	-	-	8	-
O00142	-	1	-	-	3	-	-	-	-	-	-	-
P83111	-	3	-	-	7	-	5	4	1	14	12	3
P36776	-	14	12	-	22	20	-	2	1	-	6	4
P42704	26	31	-	28	35	-	3	-	5	2	-	7
Q02978	7	6	4	26	23	12	-	4	-	-	14	-
O95819	-	-	1	-	-	3	-	-	-	-	-	-
P23368	-	-	-	-	-	-	-	-	1	-	-	7
Q9HCC0	-	-	2	-	-	7	-	-	-	-	-	-
P40926	-	-	-	-	-	-	-	13	2	-	53	11

Q9BQP7	-	-	-	-	-	-	-	1	1	-	4	8
Q9Y3D0	-	-	2	-	-	26	-	-	-	-	-	-
Q02750	-	-	-	-	-	-	-	4	1	-	12	4
Q00325	-	-	-	-	-	-	-	9	-	-	23	-
Q10713	12	13	17	43	39	49	-	-	-	-	-	-
O75439	-	12	-	-	37	-	-	-	-	-	-	-
O75439	-	-	14	-	-	42	-	-	2	-	-	10
Q7L0Y3	-	-	-	-	-	-	-	1	1	-	4	4
Q96E29	-	1	4	-	5	12	-	-	-	-	-	-
Q16795	-	-	5	-	-	17	-	-	-	-	-	-
O95299	-	9	8	-	31	32	-	1	1	-	6	6
Q5TEU4	1	-	1	5	-	5	-	-	-	-	-	-
P28331	-	-	2	-	-	3	-	1	-	-	3	-
O75306	-	5	-	-	14	-	-	2	-	-	8	-
O75489	9	7	-	42	36	-	-	4	1	-	22	6
O75251	2	2	2	11	11	11	-	-	-	-	-	-
O00217	3	2	2	24	15	10	-	-	-	-	-	-
P19404	-	-	-	-	-	-	-	-	1	-	-	4
Q13423	-	-	-	-	-	-	-	2	-	-	2	-
P04181	-	1	1	-	5	6	-	6	4	-	30	24
P12694	1	-	-	3	-	-	-	-	-	-	-	-
P21953	-	-	1	-	-	5	-	-	-	-	-	-
Q02218	-	-	-	-	-	-	-	-	1	-	-	1
P36957	-	-	-	-	-	-	-	-	2	-	-	6
P10515	-	-	-	-	-	-	-	1	2	-	2	7
P11177	-	3	6	-	10	19	-	-	-	-	-	-
O60313	-	-	4	-	-	8	-	15	5	-	22	10
P32322	-	3	6	-	21	30	-	-	1	-	-	4
Q9P0J1	10	-	-	25	-	-	-	-	-	-	-	-
Q8NCN5	6	-	-	12	-	-	-	-	-	-	-	-
Q8NCN	-	5	-	-	10	-	-	-	-	-	-	-
Q96HS1	-	-	2	-	-	9	3	-	-	11	-	-
P30405	-	-	-	-	-	-	-	-	4	-	-	35
P30048	5	4	4	30	18	18	-	-	-	-	-	-
P30044	-	1	1	-	8	8	-	3	1	-	17	8
O75127	3	2	2	5	3	3	-	-	-	-	-	-
Q96EY7	13	4	8	29	11	16	-	-	-	-	-	-
P11498	2	-	10	4	-	16	-	2	2	-	3	5
P31930	-	15	17	-	49	52	-	6	5	-	26	25
P22695	14	14	-	54	50	-	-	8	1	-	32	8
P07919	-	-	-	-	-	-	-	-	1	-	-	20
Q9BYD6	-	2	2	-	10	10	-	-	-	-	-	-
P09001	-	-	2	-	-	8	-	-	-	-	-	-
Q9BYD3	4	-	3	26	-	23	-	-	-	-	-	-
Q9BYD2	2	-	1	10	-	6	-	-	-	-	-	-

P52815	-	2	4	-	11	30	-	1	-	-	17	-
Q6P1L8	-	1	-	-	7	-	-	-	-	-	-	-
Q9P015	-	1	1	-	3	3	-	-	-	-	-	-
Q9NRX2	-	1	1	-	18	4	-	-	-	-	-	-
P49406	2	-	2	6	-	6	-	-	-	-	-	-
Q16540	-	-	2	-	-	37	-	-	-	-	-	-
Q13084	-	-	-	-	-	-	-	-	1	-	-	9
Q9NYK5	2	2	3	8	8	11	-	-	-	-	-	-
Q8IXM3	-	1	1	-	24	24	-	-	-	-	-	-
Q8N983	-	1	-	-	5	-	-	-	-	-	-	-
Q9H9J2	-	1	-	-	3	-	-	-	-	-	-	-
Q13405	2	2	2	15	15	15	-	1	1	-	7	7
Q8N5N7	-	1	2	-	13	25	-	-	-	-	-	-
Q9Y399	-	2	5	-	6	30	-	-	-	-	-	-
P82675	7	8	5	19	26	16	-	-	-	-	-	-
Q9Y2R9	-	3	8	-	14	27	-	-	-	-	-	-
P82933	-	5	5	-	15	16	-	-	-	-	-	-
P82664	-	-	4	-	-	33	-	-	-	-	-	-
P82912	-	1	-	-	15	-	-	-	-	-	-	-
O60783	-	1	2	-	12	22	-	-	-	-	-	-
P82914	-	1	3	-	4	10	-	-	-	-	-	-
Q9Y3D3	-	3	-	-	28	-	-	-	-	-	-	-
Q9Y2R5	4	3	4	52	36	52	-	-	-	-	-	-
Q9Y676	-	4	4	-	33	33	-	-	-	-	-	-
Q9Y3D5	1	1	2	18	13	18	-	-	-	-	-	-
P82921	1	-	1	14	-	14	-	-	-	-	-	-
P82650	-	11	-	-	39	-	-	-	-	-	-	-
Q9Y3D9	6	-	7	41	-	49	-	-	-	-	-	-
Q96EL2	2	-	-	16	-	-	-	-	-	-	-	-
Q9BYN8	-	2	1	-	9	5	-	-	-	-	-	-
Q92552	9	8	7	26	23	18	-	-	-	-	-	-
Q9Y2Q9	4	4	4	34	24	24	-	-	-	-	-	-
P51398	-	8	-	-	28	-	-	-	-	-	-	-
P82930	-	3	4	-	25	28	-	1	-	-	6	-
P82673	6	-	4	32	-	22	-	-	-	-	-	-
Q6NUK1	-	-	-	-	-	-	3	1	-	6	3	-
P31040	18	-	-	48	-	-	-	10	5	-	26	18
P21912	-	4	5	-	16	22	-	5	2	-	20	15
Q9NTG7	2	1	2	8	6	8	-	-	-	-	-	-
Q9GZT3	-	2	3	-	22	35	-	-	-	-	-	-
P04179	-	-	-	-	-	-	-	2	1	-	13	6
O15269	-	1	3	-	4	12	-	-	-	-	-	-
Q9Y6N5	-	-	-	-	-	-	-	1	-	-	3	-
Q9UJZ1	-	1	1	-	4	4	-	-	1	-	-	4
Q96I99	-	-	-	-	-	-	-	2	-	-	6	-

Q8IYB8	-	-	1	-	-	2	-	-	-	-	-	-
O95363	-	-	4	-	-	10	-	-	-	-	-	-
P49590	-	-	-	-	-	-	-	2	-	-	4	-
Q9NSE4	-	-	-	-	-	-	-	1	-	-	1	-
Q7L3T8	-	1	1	-	5	5	-	-	-	-	-	-
Q9NP81	-	-	1	-	-	3	-	-	-	-	-	-
Q5ST30	-	-	-	-	-	-	-	-	-	-	1	-
Q00059	-	-	1	-	-	4	-	-	-	-	-	-
P24752	-	3	5	-	8	15	-	12	2	-	38	10
P42765	-	-	-	-	-	-	-	-	1	-	-	7
Q5SRD1	-	1	1	-	4	4	-	-	-	-	-	-
P62072	1	-	2	14	-	26	-	-	-	-	-	-
Q9Y5L4	2	-	3	26	-	43	-	-	-	-	-	-
Q9Y3D7	-	-	-	-	-	-	3	1	-	33	18	-
O14925	-	1	1	-	5	5	-	-	-	-	-	-
Q9BSF4	-	2	5	-	15	30	-	-	-	-	-	-
Q3ZCQ8	-	-	-	-	-	-	-	1	1	-	3	3
O60220	4	-	4	68	-	64	-	-	-	-	-	-
Q9Y5J9	-	3	3	-	42	42	-	-	-	-	-	-
Q9Y5J7	2	2	2	29	29	29	-	-	-	-	-	-
O60784	1	-	-	3	-	-	-	-	-	-	-	-
Q15388	1	1	1	9	9	9	-	-	-	-	-	-
Q9NS69	-	-	-	-	-	-	-	1	-	-	18	-
Q15785	-	1	-	-	5	-	-	5	1	-	24	5
O94826	15	17	18	41	46	50	-	-	-	-	-	-
Q12931	-	-	1	-	2	4	-	3	-	-	11	-
Q6DKK2	-	6	3	-	25	19	-	-	-	-	-	-
Q6IBS0	-	-	-	-	-	-	-	1	-	-	5	-
P53007	2	-	2	8	-	8	-	-	-	-	-	-
P47985	-	1	-	-	9	-	-	2	-	-	8	-
